# Adult Asylum Seekers from the Middle East Including Syria in Central Europe: What Are Their Health Care Problems?

**DOI:** 10.1371/journal.pone.0148196

**Published:** 2016-02-10

**Authors:** Carmen Andrea Pfortmueller, Miriam Schwetlick, Thomas Mueller, Beat Lehmann, Aristomenis Konstantinos Exadaktylos

**Affiliations:** 1 Department of General Anaesthesiology, Intensive Care and Pain Management, Medical University of Vienna, Vienna, Austria; 2 University Department of Emergency Medicine, University Hospital and University of Bern, Bern, Switzerland; 3 University Hospital of Psychiatry and University of Bern, Bern, Switzerland; Queensland University of Technology, AUSTRALIA

## Abstract

**Background:**

Forced displacement related to persecution and violent conflict has reached a new peak in recent years. The primary aim of this study is to provide an initial overview of the acute and chronic health care problems of asylum seekers from the Middle East, with special emphasis on asylum seekers from Syria.

**Methods:**

Our retrospective data analysis comprised adult patients presenting to our emergency department between 01.11.2011 and 30.06.2014 with the official resident status of an “asylum seeker” or “refugee” from the Middle East.

**Results:**

In total, 880 patients were included in the study. Of these, 625 (71.0%) were male and 255 (29.0%) female. The median age was 34 (range 16–84). 222 (25.2%) of our patients were from Syria. The most common reason for presentation was surgical (381, 43.3%), followed by medical (321, 36.5%) and psychiatric (137, 15.6%). In patients with surgical presentations, trauma-related problems were most common (n = 196, 50.6%). Within the group of patients with medical presentation, acute infectious diseases were most common (n = 141, 43.9%), followed by neurological problems (n = 70, 21.8%) and gastrointestinal problems (n = 47, 14.6%). There were no differences between Syrian and non-Syrian refugees concerning surgical or medical admissions. The most common chronic disorder of unclear significance was chronic gastrointestinal problems (n = 132, 15%), followed by chronic musculoskeletal problems (n = 108, 12.3%) and chronic headaches (n = 78, 8.9%). Patients from Syria were significantly younger and more often suffered from a post-traumatic stress disorder than patients of other nationalities (p<0.0001, and p = 0.05, respectively).

**Conclusion:**

Overall a remarkable number of our very young group of patients suffered from psychiatric disorders and unspecified somatic symptoms. Asylum seekers should be carefully evaluated when presenting to a medical facility and physicians should be aware of the high incidence of unspecified somatic symptoms in this patient population.In general, there is no major difference between asylum seekers from Syria when compared to other nationalities of asylum seekers from the Middle East.

## Introduction

Forced displacement related to persecution and violent conflict has reached a new peak, with more than 43.3 million people worldwide fleeing from war, war-like conditions or political or ethnic oppression [1, 2]. The total number of annual applications for asylum in the European Union has increased from 15,000 to more than 300,000 over the last two decades [3]. As asylum seekers come from countries with violent conflicts and travel a hazardous journey to the country of asylum, their disease profile is strikingly different from that of the native population in the country of application [4, 5].

The Syrian civil war has become one of the worst humanitarian disasters of this century, with 2.9 million Syrian refugees [6]. According to Elizabeth Hoff, the World Health Organization’s representative to Syria for the past year, it has claimed 115,000 lives and injured more than 575,000 people [7]. Humanitarian organisations have reported the lack of nutrition, disastrous hygienic conditions and insufficient medical care in refugee camps [6, 8]. Currently not much is known about the health problems of Syrian refugees in first world countries and most of the existing studies were conducted in refugee camps in either Jordan or Turkey [8].

In Switzerland, there were 40,670 people in the asylum process in 2011 [9]. According to the Federal Bureau for Immigration, annual new applications increased from 19,750 in 2000 to 24,667 in 2012 [9]. For the duration of the asylum process, the Swiss government provides mandatory basic healthcare coverage [1]. All asylum seekers may register with a general practitioner free of charge [10]. Routine health care assessments are not performed.

There has been much international concern from health care professionals about the health of refugees in the war-torn Middle East. Nevertheless, physicians in Western countries do not have much of an understanding of the health care concerns of Middle Eastern asylum seekers. The primary aim of this study is to provide an initial overview of the acute and chronic health care problems of asylum seekers from the Middle East, with special emphasis on asylum seekers from Syria.

## Material and Methods

### Setting

Our emergency department (ED) is the Level I ED in central Switzerland, serving about 1.8 million people and treating more than 40,000 cases per year; 2.25% of our patients are asylum seekers.

### Data collection and retrospective survey

Our retrospective data analysis comprised adult patients (≥16 years) presenting to our emergency department (ED) between 1 January 2011 and 30 June 2014 with the official resident status of an “asylum seeker” or “refugee” from the Middle East. Resident status is routinely assessed by our hospital administration. Patients were identified using the appropriate search string in the patient’s demographic field of our computerised patient database (Qualicare Office, Medical Database Software, Qualidoc AG, Bern, Switzerland). In addition, all records were matched with our hospital's central patients database, where all past medical records are stored. Therefore the medical records we reviewed for the present study contained information about all diagnoses a patient had ever received in our hospital and not only from the current visit to the emergency department visit. The matching does not depend on the out- or inpatient status of a visit.

The following clinical data were extracted from medical records: reason for presentation, number of diseases, psychiatric disease (if/type) and chronic infectious diseases (if/type) were evaluated. Demographic data, such as gender and age, were evaluated. The patients classified to the trauma category suffered from acute trauma; older injuries that, for example, were attributed to the refuge journey were not counted as a reason for acute traumatic presentation. After reviewing the literature, we found no consistent definition or approach to assess multimorbidity [11]. Others have assessed multimorbidity from 7 [12] to 46 different diseases [13]. We therefore derived a new list of diseases based on the largest study by Higashi *et al* [14] and the Charlson Comorbidity Index [15]. In addition, we included psychiatric conditions (e.g. schizophrenia) as an important type of disease [16], based on a consensus of the above mentioned references and between the authors. The final list contains 18 important diseases for outpatient medicine; see Table 1.

**Table 1:** List of important diseases for outpatient medicine.

Myocardial Infarction
Heart Failure
Peripheral Vascular Disease
Cerebrovascular Disease
Dementia
COPD
Connective Tissue Disease
Peptic Ulcer Disease
Diabetes Mellitus
Moderate to Severe Chronic Kidney Disease
Malignant Tumour (solid or non-solid)
Liver Disease
Psychiatric Disease (any type)
Osteoporosis
Hypertension
Dyslipidaemia
Asthma
Chronic Infectious Disease

The severity of multimorbidity was assessed by the Charlson Comorbidity Index [15]. The Charlson Comorbidity Index is calculated based on points given to predefined medical conditions such as a history of myocardial infarction, active neoplastic disease etc. [15]. Additional points are given for the age of the patient [15]. Chronic medical symptom complexes of unclear origin were defined as self-reported somatic health problems of more than one month duration for which no somatic diagnosis could be found, despite state-of-the-art medical evaluation (e.g. endoscopy to rule out peptic ulcer disease, cerebral imaging in patients with headaches). These included: history of anorexia, history of sleep disorder, history of chronic dizziness, chronic musculoskeletal pain, chronic gastrointestinal problems (e.g. pain, indigestion) and chronic headaches. Acute infectious diseases were defined as any transient type of infection (e.g. otorhinolaryngeal, gastrointestinal, urinary) leading to ED admission. Chronic infectious diseases were defined as chronic underlying infections, such as human retrovirus infection (HIV), hepatitis B or C, syphilis, parasitic infections and tuberculosis. All medical records were reviewed by two specialists in internal medicine and a specialist in emergency medicine. Records of patients with psychiatric disorders were discussed with a psychiatrist. Re-presentations excluded follow-up admission for further investigations at our hospital and were only counted if the reason for representation was different and unrelated to the initial presentation with an interval between presentations of at least one month.

The geographical definition of Middle East varies [17]; we decided to use the traditional definition of the Middle East, including patients from the following countries: Bahrain, Iran, Iraq, Israel, Jordan, Kuwait, Lebanon, Oman, Qatar, Saudi Arabia, Turkey, United Arab Emirates, Yemen and Syria.

Patients (n = 58) with incomplete medical records (rudimentary history, lack of diagnosis and systemic history), duplicated records and recurrent presentations, were excluded from the analysis.

### Statistical Analysis

All statistical analyses were performed with the SPSS 20.0 Statistical Analysis program (SPSS Inc; Chicago, IL). The data were summarised using descriptive statistics (means and standard deviation or medians and range as appropriate, counts and percentages). Differences in characteristics and regional origin between patients were tested using chi-squared test for categorical variables and Kruskal-Wallis ANOVA for interval and ordinal variables. Post-hoc testing was performed using the Mann-Whitney U test. All p values were two tailed and at a level of significance of 0.05.

### Ethical Considerations

The study was approved by the Ethics Committee of the Canton of Bern, Switzerland. Individual patient consent was not obtained, but was waived by the Ethics Committee. Patient records/information was anonymised and de-identified prior to analysis.

## Results

**Fig 1:**
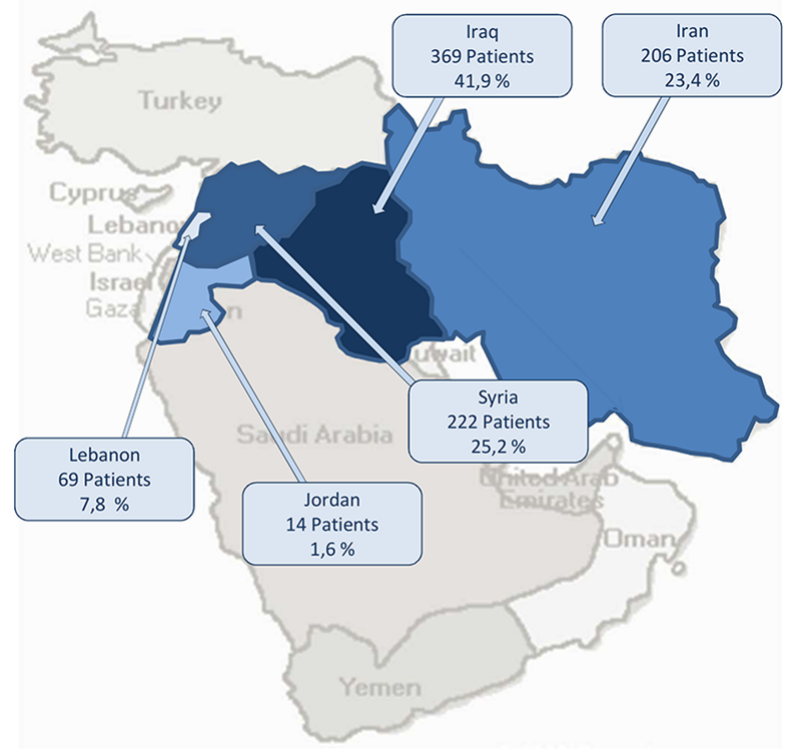
Overview of patients' origins.

**Fig 2:**
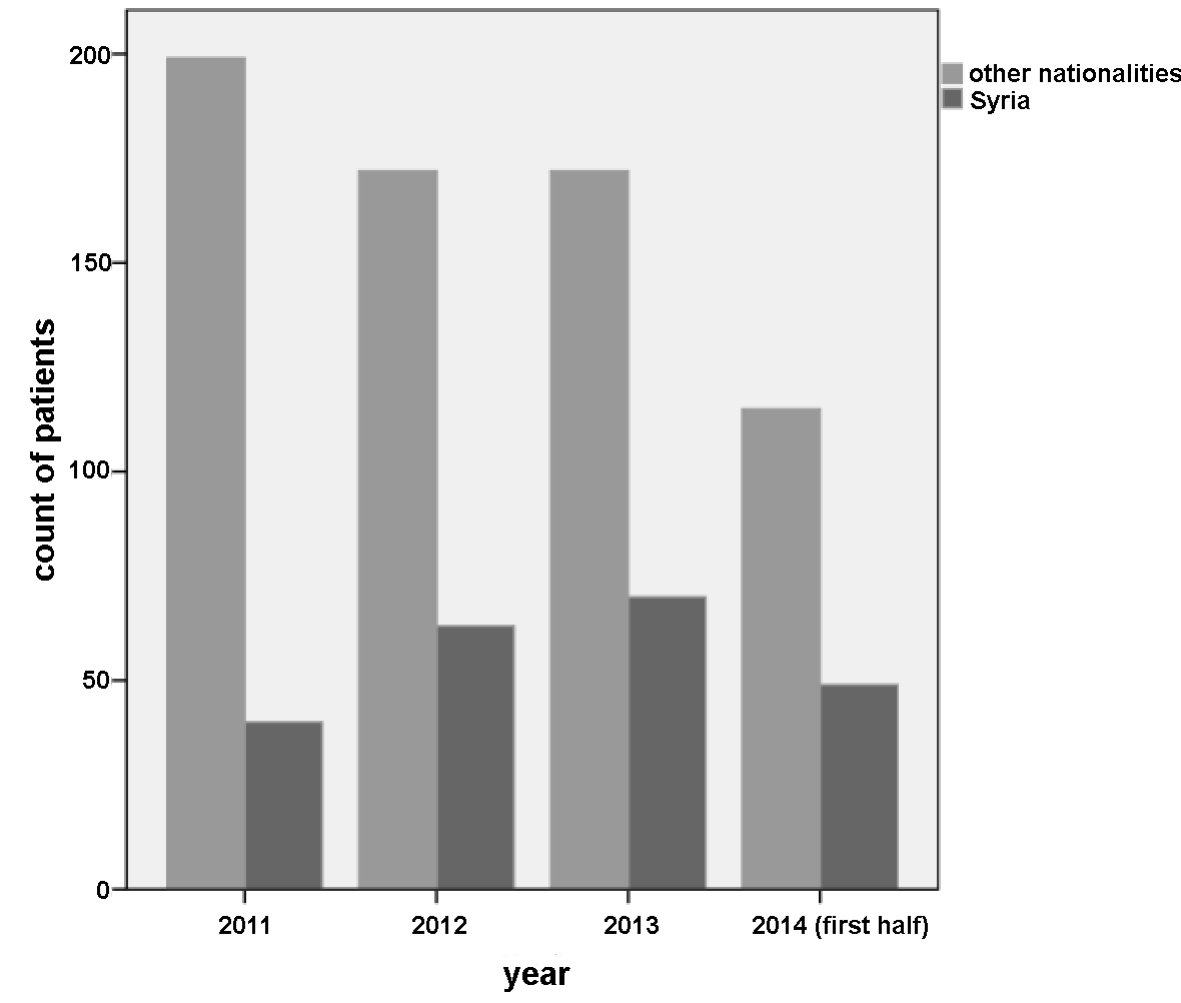
Number of patients presenting to emergency department by year and country of origin.

In total, 880 patients were included in the study. Six hundred and twenty-five (625; 71.0%) of the patients were male, 255 (29.0%) female. The median age was 34 (range 16–84). Two hundred and twenty-two (222, 25.2%) of our patients were from Syria. For an overview of the origins of our patients, see Fig 1. Seventy-four (74, 8.4%) of our patients were hospitalised and 472 (53.8%) came to our emergency department within the study period. Walk-in patients accounted for 95% percent (836) of our patients. For an overview of patient characteristics, see Table 2. From 2011 to 2014, there was an increase in patients from Syria presenting to your emergency department (p = 0.004); see Fig 2.

**Table 2:** Patient characteristics.

	Overall N	(%)	Syrian N (%)	non-Syrian N (%)	p value
	880	(100)	222 (25.2)	658 (74.8)	
male	625	(71.0)	156 (70.0)	469 (71.2)	0.77
female	255	(29.0)	66 (30.0)	189 (28.9)	
age (median, range, 95% CI)	34 (16–84)		33 (12.94, 31.7–35.8)	37 (11.59, 35.9–37.7)	0.0001
**Country of Origin**					
Iraq	369	(41.9)			
Iran	206	(23.4)			
Jordan	14	(1.6)			
Lebanon	69	(7.8)			
Syria	222	(25.2)			
**Reason for ED Presentation**					0.28
medical	321	(36.5)	73 (32.8)	248 (37.6)	
surgical	381	(43.3)	106 (47.7)	275 (41.7)	
psychiatric	137	(15.6)	35 (15.7)	102 (15.5)	
other (ENT, gynaecological, dermatological)	41	(4.6)	8 (3.6)	41 (6.2)	
**Mean count of Comorbidities (SD, range)**	1.07 (1.91, 0–9)				
Total Count of Patients with Comorbidities	322	(36.7)			
***Type of Comorbidity***					0.37
chronic medical condition of unclear significance	89	(10.2)	25 (11.2)	64 (9.7)	
psychiatric	211	(24.0)	45 (20.2)	166 (25.2)	
infectious	22	(2.5)	9 (4.1)	15 (2.3)	
**Charlson Co-Morbidity Index (mean, SD, 95% CI)**	0.18 (0.68)		0.2 (0.95, 0.08–0.33)	0.18 (0.55, 0.13–0.22)	
**Hospitalisation rate**	74	(8.4)	19 (8.6)	55 (8.4)	0.69
**Readmission to ED during study period**	472	(53.8)	119 (53.6)	354 (53.7)	0.92

### Reasons for presentation

The most common reason for presentation was surgical (381, 43.3%), followed by medical (321, 36.5%) and psychiatric (137, 15.6%). In patients with surgical presentations, trauma-related problems were most common (n = 196, 50.6%). Thirty-seven (37.0%) percent of the traumas were a consequence of violence. Within the group of patients with medical presentation, acute infectious diseases were most common (n = 141, 43.9%), followed by neurological problems (n = 70, 21.8%) and gastrointestinal problems (n = 47, 14.6%).

### Co-morbidities

**Fig 3:**
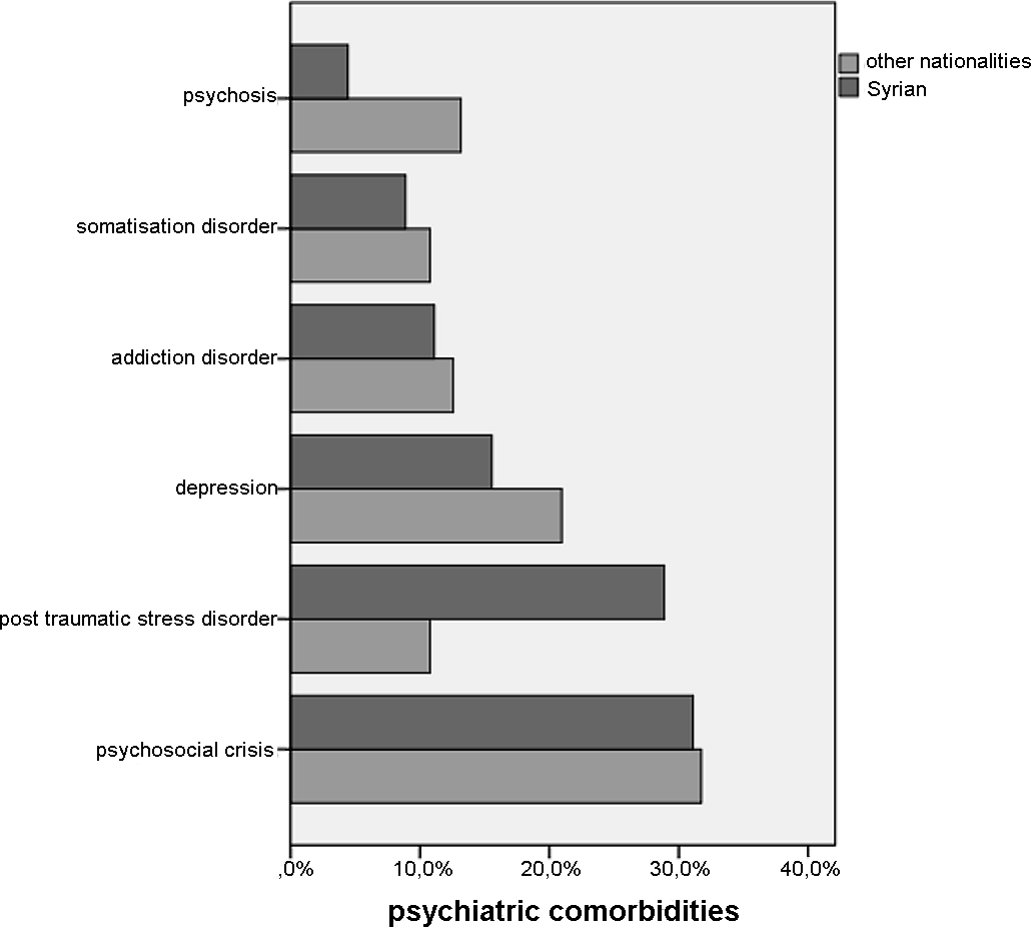
Overview of psychiatric co-morbidities by country of origin.

Overall 322 (36.7%) of our patients had a comorbidity, with a mean comorbidity count of 1.07 (SD 1.91) and Charlson Co-Morbidity Index of 0.18 (SD 0.68). Two hundred and eleven (24.0%) of our patients suffered from a psychiatric comorbidity. The most common diagnosis was acute psychosocial crisis (31.6%), followed by depression (19.9%) and post-traumatic stress disorder (14.6%). For an overview of psychiatric co-morbidities see Fig 3.

Patients from Syria significantly more often suffered from a post-traumatic stress disorder (p = 0.05) than others. Alcohol abuse was more common in patients from Syria than elsewhere (p = 0.014).

The most common chronic disorder of unclear significance was chronic gastrointestinal problems (n = 132, 15.0%), followed by chronic musculoskeletal problems (n = 108, 12.3%) and chronic headaches (n = 78, 8.9%). There were no significant differences in chronic medical conditions of unclear origin between patients from Syria and other nationalities.

### Syrian versus non-Syrian patients

Patients from Syria were significantly younger than patients from other nationalities (p<0.0001). There was no difference in sex (p = 0.77), overall reason for admission or count of comorbidities (p = 0.62). There were no differences between Syrian and non-Syrian refugees with respect to the proportion of walk-in patients, or in hospitalisation rates (p = 0.69 or 0.92, respectively). There was no difference between Syrian and non-Syrian patients in the frequency of trauma-related admissions or underlying violence (p = 0.27 or p = 0.09, respectively). There were no significant differences in chronic medical conditions of unclear origin between patients from Syria and other nationalities. For an overview of differences between Syrian and non-Syrian refugees, see Table 2.

## Discussion

A total of 880 asylum seekers were eligible for the study. Most of our patients were young males in their second or third decade of life. This age distribution is typical for asylum seekers, especially Middle Eastern and African asylum seekers, as a previous study by our group showed [2, 18, 19]. There is no established explanation for this phenomenon. Firstly, It may be speculated that young men are more likely than young women to have the strength to make the hazardous journey to the country of application for asylum. Secondly, the young men may be more likely to seek asylum first in order to seek protection for their family later on. Thirdly, their religious background may not allow women to be independent and travel alone.

In our study, surgical reasons for presentation to the ED, especially trauma, were most common, followed by medical and psychiatric problems. Exact figures on the types of diseases and reasons for presentation are scarce. According to Buhmann et al, 36% of asylum seekers from the Middle East (mainly Iran, Iraq and Lebanon) suffered from somatic problems [18]. These were most commonly neurological, especially headaches and gastrointestinal problems [18, 19]. This figure is quite comparable to our findings. Nonetheless caution is required when comparing our findings with those from other studies on the health care needs of adult asylum seekers, as these have been conducted in a wide variety of settings (hospital, refugee camp, asylum home etc.) and have concentrated on different issues (hygiene, living conditions, acute health issues, chronic health issues etc.).For example the study by Buhmann et al was conducted in a refugee camp, whereas our study was conducted in a public emergency department. Therefore it is possible that acute health care problems such as respiratory infections and acute trauma are not as common as in a refugee camp, whereas nutritional and hygienic problems are certainly more common in refugee camps.

In the present study we have a very low mean count of comorbidity and a very low Charleson Co-Morbidity Index. This finding is consistent with earlier work [5]. We attribute this to the relative youth of the asylum seeker population, as they are at low risk of cardiovascular or oncological problems. Nonetheless, more than 36% of our young patient group suffered from at least one comorbidity, of which psychiatric comorbidities are the most common.

Overall 24% of our patients suffered from a psychiatric comorbidity. Psychosocial crisis and post-traumatic stress disorder were the most common types of psychiatric disease. Asylum seekers from the Middle East may have lived in war and war-like conditions most of their lives and are therefore especially prone to develop post-traumatic stress syndrome [19, 20]. The reason for the high prevalence of post-traumatic stress disorder is three-fold: Firstly, traumatised refugees experience accumulated and severe trauma, such as torture, imprisonment, loosing loved ones etc. [19]. Secondly, they are a consequence of the asylum process itself. The latter is connected to a long phase of waiting in uncertainty and under unfavourable living conditions [5, 19, 21, 22]. Thirdly, according to a recent Australian multicentre study on refugees and post-traumatic stress disorder, the stress of settling into a new culture, living in isolation, poverty and meeting intolerance and racism further traumatises asylum seekers and can further aggravate post-traumatic stress symptoms [23].

Some of our study population suffered from “unexplained” bodily symptoms despite a thorough medical work-up. Buhmann et al introduced a new concept for unexplained somatic symptoms in the asylum seeker population—the so called “Bodily Distress Syndrome” [19, 24, 25]. According to Fink et al, the diagnosis itself requires three or more symptoms from at least three of the following categories: musculoskeletal (muscle and joint pain, numbness and localised weakness), gastrointestinal (constipation, diarrhoea, abdominal pain, regurgitation, nausea and vomiting), cardiovascular (palpitations, breathlessness, hot and cold sweats, dry mouth, flushing and trembling) or general symptoms (dizziness, headache, fatigue, memory impairment and concentration difficulties). The symptoms should not be explicable by other somatic disease [24].The causes of the syndrome is thought to be either dysfunction of the hypothalamic-pituitary-adrenal axis or autonomic regulation of physiological arousal [24].

Even though we did not specifically screen for Bodily Distress Syndrome it is possible that this concept may also apply to our patient group. Nevertheless, the term “Bodily Distress Syndrome” should be used with caution and it must always be carefully evaluated. whether the patient's complaints have a somatic background Despite the interest in finding a diagnosis for the “unexplained” complaints by asylum seekers, the concept of the Bodily Distress Syndrome must be thoroughly scientifically evaluated before being generally adopted.

To the best of our knowledge, the present study is the first focusing on health care problems of Middle Eastern refugees with special emphasis on Syrian refugees. There is literally no study available on general health care problems of Syrian refugees. Almost all the studies on Syrian refugees were conducted in refugee camps and moreover they mostly focus on one distinct health care issue, such as pulmonary conditions [26, 27], obstetric issues and paediatric care. Overall there was no major difference between asylum seekers from Syria and from other countries of the Middle East, although patients from Syria were slightly younger and were more often diagnosed with post-traumatic stress disorder. Moreover, they were more often addicted to alcohol.

As a conclusion, it can be said that there is probably no difference between asylum seekers from Syria when compared to other nationalities of asylum seekers from the Middle East. We consider it probable that the acute and chronic health care problems of asylum seekers are mostly related to the asylum seeker status (e.g. living conditions, psychological stress of detention-like environment) and to their cultural backgrounds rather than to their actual nationality.

### Limitations

This study has to be interpreted with some caution as it has several limitations. This was a retrospective study: As no standardised general and systemic medical history was taken, it is possible that our numbers may actually underestimate the prevalence of certain diseases or symptoms. Due to the retrospective design, chronic medical conditions may not have been detected, as their detection relies on patients' self-reporting and if patients had been treated in another hospital or by a primary care physician for those problems they might have been missed. This study provides an initial overview of the health care problems of Middle Eastern asylum seekers, including patients from Syria; further prospective studies should be conducted. In addition, an emergency department—where patients are often treated with limited time and personal resources—may not be the most favourable place to study multimorbidity in asylum seekers. Therefore our figures may underestimate the actual multimorbidity in this population. Moreover, multimorbidity may be assessed in different ways. [12, 28].The Charlson Multimorbidity Index that we used may not be the ideal tool to detect multimorbidity, as it is based on the mere absence or presence of diseases, in contrast to the Adjusted Clinical Groups (ACG) System [28]. The latter is the only commonly used method that is based on combinations of different types of diagnoses over time, rather than the presence or absence of particular conditions or numbers of conditions [28]. It is therefore possible that our results were biased by the methods we used to assess multimorbidity.

False assessment of nationality and geographic region cannot be excluded. We do not have any further information on the true origins of the Syrian refugees in our study population. All patients whose nationality was confirmed as “Syrian” by the Swiss Immigrants office and given the status “asylum seeker” are included in this study. We are therefore not able to differentiate whether people where born of Syrian parents or former refugees from Iraq and we do not have any information as to how long our patients travelled to Switzerland and how long they had been recognised asylum seekers before presentation to our emergency department. Furthermore, as this is a single centre study, external validity is not given. Additionally, we only assessed patients with the status of “asylum seeker/refugee”. We did not assess patients with a residence permit but from outside Switzerland. Therefore our data does not represent an analysis of the health care problems of persons of a specific nationality. Moreover, we cannot provide any information on the medical conditions of children or pregnant females, as those are treated in a different ED within our hospital.

## Conclusion

Our study is the first to present data on the health care problems of Middle Eastern refugees and asylum seekers in a European country with a special emphasis on refugees from Syria. Overall a remarkable number of our very young patients collective suffered from psychiatric disorders and unspecified somatic symptoms. The later may be an indication of “Bodily Distress Syndrome” in this population, but further prospective studies on the topic are certainly needed. Asylum seekers should be carefully evaluated when presenting to a medical facility and physicians should be aware of the high incidence of unspecified somatic symptoms in this patient population.

In general, there is no difference between asylum seekers from Syria when compared to other nationalities of asylum seekers from the Middle East. We consider that the acute and chronic health care problems of asylum seekers are probably mostly related to the asylum seeker status (e.g. living conditions, psychological stress of detention-like environment) and to cultural differences rather than to the actual nationality.

## Supporting Information

S1 Table (PDF)
