# Supplementary material for: Adult Asylum Seekers from the Middle East Including Syria in Central Europe: What Are Their Health Care Problems?
Source: PLoS One. 2016 Feb 10;11(2):e0148196. doi: 10.1371/journal.pone.0148196 (PMC4749343; doi:10.1371/journal.pone.0148196)
Supplement: S1 Table — (PDF) [file pone.0148196.s001.pdf]

sex	Alter	nationality	SYRIEN	Jahr	Wiedervorstellung	AnzahlWiedervorstellungen
2	26.6082	4	0	2012	1.00	0
2	44.2493	3	0	2012	1.00	0
3	42.1562	2	0	2012	2.00	3
3	28.7096	3	0	2012	1.00	0
2	31.8849	3	0	2012	2.00	3
2	20.0493	6	1	2012	2.00	15
2	36.5315	5	0	2012	2.00	2
3	25.8110	2	0	2012	1.00	0
2	39.8685	2	0	2012	1.00	0
3	37.9589	2	0	2012	2.00	2
3	58.9041	6	1	2012	1.00	0
3	35.8767	2	0	2012	1.00	0
3	17.5014	2	0	2012	2.00	2
3	27.4438	2	0	2012	2.00	2
2	26.7370	4	0	2012	2.00	2
2	50.6137	6	1	2012	2.00	2
2	40.4877	3	0	2012	2.00	4
2	25.7918	6	1	2012	1.00	0
2	39.5616	3	0	2012	2.00	4
2	29.7151	3	0	2012	1.00	0
2	24.6411	2	0	2012	2.00	2
2	36.5041	5	0	2012	1.00	0
3	31.8438	3	0	2012	1.00	0
2	24.5644	2	0	2012	2.00	4
2	40.1644	3	0	2012	2.00	4
2	36.4137	2	0	2012	2.00	4
2	31.2384	2	0	2012	1.00	0
2	41.4904	5	0	2012	2.00	7
2	54.9370	3	0	2012	2.00	3
2	42.7397	3	0	2012	2.00	2
2	41.5096	2	0	2012	2.00	6
2	38.6164	2	0	2012	1.00	0
2	45.0767	2	0	2012	1.00	0
2	36.0849	4	0	2012	2.00	3
2	54.7781	2	0	2012	2.00	3
2	72.4603	2	0	2012	1.00	0
2	40.0630	3	0	2012	1.00	0
2	51.9342	3	0	2012	2.00	24
2	50.7726	3	0	2012	2.00	2
3	26.4959	2	0	2012	1.00	0
3	34.4795	2	0	2012	1.00	0
3	27.8685	3	0	2012	2.00	3
2	21.9616	2	0	2012	1.00	0
3	28.2082	3	0	2012	1.00	0
2	40.8384	2	0	2012	2.00	3
2	40.7781	3	0	2012	2.00	2
3	41.1014	5	0	2012	1.00	0
3	25.4603	5	0	2012	2.00	3
3	24.2137	2	0	2012	1.00	0
3	28.4219	3	0	2012	1.00	0
3	28.2274	5	0	2012	1.00	0
3	59.2712	2	0	2012	1.00	0
2	30.9890	3	0	2012	1.00	0
2	34.5890	2	0	2012	1.00	0
2	50.1260	4	0	2012	1.00	0
3	26.1644	3	0	2012	1.00	0
3	34.2986	2	0	2012	1.00	0
2	29.7397	6	1	2012	1.00	0
2	22.1918	5	0	2012	1.00	0
2	45.4822	3	0	2012	2.00	4
2	23.7589	3	0	2012	2.00	3
3	46.6438	2	0	2012	1.00	0
2	28.2493	3	0	2012	2.00	2
3	31.4740	2	0	2012	1.00	0
2	47.5068	3	0	2012	2.00	2
2	29.1178	5	0	2012	1.00	0
2	24.7260	2	0	2012	1.00	0
2	52.5205	2	0	2012	1.00	0
2	25.4219	2	0	2012	1.00	0
2	25.7205	2	0	2012	1.00	0
3	44.1671	3	0	2012	1.00	0
2	44.9342	3	0	2012	1.00	0
3	37.4219	3	0	2012	1.00	0
2	53.4411	3	0	2012	2.00	3
2	52.7507	2	0	2012	1.00	0
2	34.8630	6	1	2012	1.00	0

2	24.9699	4	0	2012	1.00	0
3	31.2274	5	0	2012	1.00	0
2	38.2877	2	0	2012	1.00	0
3	26.0904	2	0	2012	1.00	0
2	22.4849	2	0	2012	1.00	0
2	39.5753	3	0	2012	2.00	4
3	32.2740	2	0	2012	1.00	0
3	27.0767	2	0	2012	1.00	0
2	43.5205	2	0	2012	1.00	0
3	40.9096	2	0	2011	1.00	0
3	54.5452	2	0	2011	1.00	0
2	38.0356	3	0	2011	2.00	4
2	38.5425	2	0	2011	2.00	3
3	23.3699	2	0	2011	1.00	0
2	25.5479	2	0	2011	2.00	3
3	35.1616	3	0	2011	2.00	2
2	30.9616	5	0	2011	2.00	3
2	52.0712	2	0	2011	2.00	3
3	31.4795	2	0	2011	1.00	0
2	27.2493	5	0	2011	2.00	3
2	42.7753	3	0	2011	2.00	3
2	36.6603	2	0	2011	1.00	0
3	41.0082	3	0	2011	2.00	2
2	25.8521	2	0	2011	1.00	0
2	28.6164	6	1	2011	2.00	4
2	35.4055	3	0	2011	1.00	0
2	28.5205	2	0	2011	1.00	0
2	57.6493	2	0	2011	2.00	2
2	45.4219	3	0	2011	1.00	0
2	51.6603	3	0	2011	1.00	0
3	25.5616	6	1	2011	2.00	4
2	25.1973	2	0	2011	2.00	2
3	54.6630	2	0	2011	1.00	0
3	43.1616	6	1	2011	2.00	2
2	47.1644	2	0	2011	2.00	5
3	32.7671	3	0	2011	2.00	2
2	56.6658	2	0	2011	2.00	2
3	26.1479	2	0	2011	2.00	2
2	38.6740	2	0	2011	2.00	3
3	37.6219	2	0	2011	1.00	0
2	40.0767	5	0	2011	2.00	7
2	84.9945	3	0	2011	1.00	0
3	71.2164	2	0	2011	2.00	3
3	49.3973	3	0	2011	1.00	0
2	47.7178	2	0	2011	2.00	3
2	35.0959	5	0	2011	1.00	0
2	24.1507	6	1	2011	2.00	3
2	47.2192	2	0	2011	2.00	5
3	56.2274	6	1	2011	2.00	3
3	39.2192	2	0	2011	2.00	4
2	30.8630	2	0	2011	2.00	2
2	45.7699	3	0	2011	1.00	0
2	29.5452	5	0	2011	2.00	2
2	31.8301	2	0	2011	1.00	0
3	71.2658	2	0	2011	2.00	3
3	30.6630	2	0	2011	1.00	0
2	33.1397	6	1	2011	2.00	3
3	28.5781	3	0	2011	1.00	0
2	44.0986	3	0	2011	2.00	4
2	31.1479	2	0	2011	1.00	0
2	35.2493	6	1	2011	1.00	0
3	30.6795	2	0	2011	1.00	0
2	27.8795	2	0	2011	2.00	2
2	50.1205	6	1	2011	1.00	0
2	54.2849	3	0	2011	1.00	0
2	32.7945	2	0	2011	1.00	0
2	19.6082	2	0	2011	1.00	0
2	44.3041	2	0	2011	1.00	0
3	33.4630	2	0	2011	1.00	0
2	54.5479	3	0	2011	1.00	0
2	33.5370	2	0	2011	1.00	0
3	56.8301	2	0	2011	1.00	0
2	32.1096	3	0	2011	1.00	0
3	25.7342	6	1	2011	2.00	4
2	40.2247	5	0	2011	2.00	7
2	39.6466	2	0	2011	2.00	2
2	20.5479	3	0	2011	1.00	0

3	20.4137	6	1	2011	2.00	5
3	39.6411	2	0	2011	2.00	2
3	17.3397	2	0	2011	1.00	0
2	28.6575	2	0	2011	1.00	0
3	25.7781	6	1	2011	2.00	4
2	49.5836	3	0	2011	2.00	2
3	43.8767	2	0	2011	2.00	4
2	40.9644	2	0	2011	2.00	2
2	37.2822	5	0	2011	1.00	0
2	16.5178	3	0	2011	1.00	0
2	27.6877	5	0	2011	2.00	2
2	39.7068	2	0	2011	2.00	2
2	32.7151	2	0	2011	1.00	0
2	32.3096	2	0	2011	2.00	2
2	38.9041	2	0	2011	2.00	3
2	24.0301	2	0	2011	1.00	0
2	45.2027	3	0	2011	1.00	0
2	45.3397	2	0	2011	1.00	0
2	27.8274	6	1	2011	2.00	4
2	47.5863	3	0	2011	2.00	3
2	32.9726	4	0	2011	1.00	0
2	27.8384	6	1	2011	2.00	4
2	57.3014	6	1	2011	1.00	0
2	27.4000	6	1	2011	2.00	2
2	35.3534	5	0	2011	2.00	2
2	33.9205	2	0	2011	2.00	2
2	27.7452	5	0	2011	2.00	2
3	49.4849	2	0	2011	1.00	0
2	39.8164	5	0	2011	2.00	3
2	34.0192	6	1	2011	2.00	3
2	47.9644	2	0	2011	2.00	3
2	41.8301	3	0	2011	1.00	0
2	28.9973	6	1	2011	2.00	2
2	47.4658	3	0	2011	1.00	0
3	58.4164	3	0	2011	1.00	0
3	40.0603	3	0	2011	2.00	2
3	20.5397	6	1	2011	2.00	5
2	18.2329	2	0	2011	1.00	0
2	36.0055	2	0	2011	2.00	2
2	40.4329	3	0	2011	2.00	3
3	34.3315	5	0	2011	1.00	0
2	36.1918	6	1	2011	1.00	0
3	52.5425	2	0	2011	1.00	0
2	40.3973	5	0	2011	2.00	7
2	19.0986	6	1	2011	2.00	15
3	23.1068	5	0	2011	1.00	0
2	42.8082	2	0	2011	1.00	0
2	30.8192	5	0	2011	2.00	2
2	24.2548	2	0	2011	2.00	2
2	22.5890	2	0	2011	2.00	8
2	26.0110	2	0	2011	1.00	0
3	25.9370	6	1	2011	2.00	4
2	37.5014	3	0	2011	2.00	3
2	27.9699	2	0	2011	1.00	0
3	37.4411	3	0	2011	1.00	0
2	38.2137	2	0	2011	2.00	4
2	17.4877	6	1	2011	2.00	2
2	20.6247	2	0	2011	2.00	2
2	34.4329	2	0	2011	2.00	3
2	45.7781	5	0	2011	1.00	0
2	57.0438	2	0	2011	2.00	2
2	38.3370	3	0	2011	1.00	0
3	44.0521	2	0	2011	1.00	0
2	53.0575	2	0	2011	2.00	3
2	37.5260	3	0	2011	2.00	3
2	41.6137	3	0	2011	2.00	2
2	34.6329	2	0	2011	1.00	0
3	38.0603	2	0	2011	2.00	2
3	29.7151	2	0	2011	1.00	0
3	32.7726	5	0	2011	1.00	0
2	37.5589	3	0	2011	2.00	3
2	27.5753	2	0	2011	2.00	5
2	19.1863	6	1	2011	2.00	15
2	34.1616	6	1	2011	2.00	3
2	31.4959	5	0	2011	2.00	3
3	18.8274	6	1	2011	2.00	2
2	22.2521	2	0	2011	2.00	2

2	19.1945	6	1	2011	2.00	15
2	27.5890	2	0	2011	2.00	5
2	34.1233	3	0	2011	1.00	0
2	34.0795	2	0	2011	2.00	2
2	31.1260	2	0	2011	2.00	4
2	27.5973	2	0	2011	2.00	5
2	41.7973	5	0	2011	2.00	2
2	33.4986	6	1	2011	2.00	3
2	38.3014	2	0	2011	2.00	4
2	40.5178	5	0	2011	2.00	7
2	29.9342	5	0	2011	2.00	2
2	42.9205	2	0	2011	1.00	0
2	30.4849	5	0	2011	1.00	0
2	29.9041	3	0	2011	1.00	0
3	71.6575	2	0	2011	2.00	3
2	41.3562	5	0	2011	2.00	2
2	35.1425	2	0	2011	2.00	4
2	35.5644	5	0	2011	2.00	2
2	31.3973	2	0	2011	1.00	0
2	24.5945	6	1	2011	2.00	3
3	22.5671	6	1	2011	2.00	3
3	19.0493	3	0	2011	2.00	2
3	25.4658	3	0	2011	2.00	2
3	31.4110	3	0	2011	2.00	2
3	41.0521	3	0	2011	1.00	0
2	29.2219	6	1	2011	2.00	2
2	33.0164	2	0	2011	2.00	2
2	67.2137	2	0	2011	1.00	0
2	47.5616	6	1	2011	1.00	0
2	23.7014	2	0	2011	1.00	0
3	41.1205	2	0	2011	2.00	3
2	27.0438	5	0	2011	2.00	2
2	53.2356	2	0	2011	2.00	3
2	24.9068	3	0	2011	2.00	2
2	29.8137	2	0	2011	1.00	0
2	18.8795	5	0	2011	1.00	0
2	27.7315	2	0	2011	2.00	5
2	31.6466	5	0	2011	2.00	3
3	32.6438	6	1	2011	1.00	0
2	56.7863	3	0	2011	2.00	2
2	20.8411	2	0	2011	2.00	2
3	21.9315	2	0	2011	2.00	3
3	32.2575	2	0	2011	2.00	2
2	23.8932	6	1	2011	1.00	0
3	21.4192	2	0	2011	1.00	0
2	22.8493	2	0	2011	2.00	3
2	34.6740	2	0	2011	2.00	3
2	35.1781	4	0	2011	2.00	3
3	30.9726	6	1	2011	2.00	2
2	36.3151	2	0	2011	2.00	2
2	25.9041	2	0	2011	2.00	3
2	34.6877	2	0	2011	2.00	3
3	20.9644	2	0	2011	1.00	0
2	24.9808	3	0	2011	2.00	2
2	27.5425	2	0	2011	1.00	0
2	24.5452	2	0	2011	2.00	8
2	21.4411	3	0	2011	1.00	0
2	35.3014	2	0	2011	2.00	4
2	49.3178	2	0	2011	1.00	0
2	26.3096	2	0	2011	2.00	3
3	44.3260	2	0	2011	2.00	2
2	28.8137	6	1	2011	2.00	10
2	53.3342	2	0	2011	2.00	3
2	22.8959	2	0	2011	1.00	0
2	37.3288	2	0	2011	1.00	0
2	27.1589	5	0	2011	2.00	2
2	31.2356	2	0	2011	1.00	0
2	40.7562	2	0	2011	2.00	6
2	35.0027	2	0	2011	2.00	2
2	22.9726	3	0	2011	2.00	3
2	22.9397	2	0	2011	2.00	8
3	49.0849	2	0	2011	1.00	0
2	42.5397	6	1	2011	1.00	0
3	16.4904	2	0	2011	2.00	2
2	33.2055	2	0	2011	2.00	2
2	19.0575	2	0	2011	2.00	2
3	31.0767	6	1	2011	2.00	2

2	31.2027	5	0	2011	2.00	2
2	40.2219	3	0	2011	1.00	0
3	19.1781	2	0	2011	2.00	2
2	25.9233	6	1	2011	2.00	2
3	48.4466	2	0	2011	2.00	3
2	32.4384	2	0	2011	1.00	0
2	47.9452	2	0	2011	2.00	5
2	18.9178	2	0	2011	1.00	0
2	22.2466	2	0	2011	1.00	0
3	28.9699	6	1	2011	2.00	2
3	37.2932	2	0	2011	1.00	0
2	23.6137	2	0	2011	1.00	0
2	54.2685	3	0	2011	2.00	2
2	50.0027	6	1	2011	2.00	3
3	43.2795	2	0	2011	1.00	0
2	32.2849	3	0	2011	1.00	0
2	34.1123	2	0	2011	1.00	0
3	37.6301	3	0	2012	2.00	2
3	29.0192	6	1	2012	2.00	2
2	39.8712	3	0	2012	2.00	4
2	19.2082	2	0	2012	2.00	2
2	41.5342	2	0	2012	2.00	2
2	28.6438	2	0	2012	2.00	2
2	42.0301	3	0	2012	1.00	0
2	42.3123	5	0	2012	1.00	0
3	30.6192	2	0	2012	1.00	0
2	32.4027	2	0	2012	1.00	0
2	40.9699	2	0	2012	2.00	6
2	33.9589	3	0	2012	1.00	0
3	44.5945	2	0	2012	2.00	2
2	53.0986	2	0	2012	2.00	3
3	19.4904	3	0	2012	2.00	2
3	26.4137	5	0	2012	1.00	0
2	30.4247	3	0	2012	1.00	0
2	33.4658	2	0	2012	1.00	0
3	27.0712	6	1	2012	1.00	0
3	33.7014	3	0	2012	1.00	0
2	19.7425	6	1	2012	2.00	15
3	33.4219	3	0	2012	1.00	0
2	29.9342	6	1	2012	1.00	0
2	31.8000	2	0	2012	2.00	2
2	26.1534	2	0	2012	1.00	0
3	23.0137	2	0	2012	2.00	2
3	48.6849	2	0	2012	2.00	3
3	27.2438	3	0	2012	2.00	3
3	72.7068	2	0	2012	1.00	0
3	35.7808	2	0	2012	1.00	0
2	31.3288	3	0	2012	2.00	3
2	35.4438	2	0	2012	1.00	0
3	20.3589	6	1	2012	1.00	0
2	38.7616	2	0	2012	2.00	2
2	36.1315	5	0	2012	1.00	0
3	44.7233	2	0	2012	2.00	4
2	31.3507	3	0	2012	2.00	3
2	29.7370	6	1	2012	2.00	4
2	27.5014	3	0	2012	2.00	2
2	30.6082	2	0	2012	1.00	0
3	32.4795	6	1	2012	2.00	2
3	21.6548	6	1	2012	2.00	5
2	29.2575	6	1	2012	2.00	10
2	41.7781	2	0	2012	2.00	2
2	28.4521	5	0	2012	2.00	3
2	29.7726	6	1	2012	2.00	2
2	30.6575	3	0	2012	2.00	2
2	29.7808	6	1	2012	2.00	2
2	30.4329	2	0	2012	2.00	2
2	30.6192	3	0	2012	2.00	4
3	32.0274	3	0	2012	2.00	2
2	41.2438	3	0	2012	2.00	3
2	29.2877	6	1	2012	2.00	10
3	35.2904	2	0	2012	1.00	0
2	38.9836	2	0	2012	2.00	4
2	25.0411	2	0	2012	2.00	8
2	36.7534	2	0	2012	1.00	0
2	25.0438	2	0	2012	2.00	8
2	30.6384	3	0	2012	2.00	4
3	16.7644	3	0	2012	2.00	2

2	30.1342	2	0	2012	2.00	2
2	45.1507	3	0	2012	2.00	4
2	51.4000	3	0	2012	2.00	24
2	43.6110	3	0	2012	1.00	0
3	36.4438	3	0	2012	2.00	2
2	35.4192	3	0	2012	2.00	4
2	35.4247	3	0	2012	2.00	4
2	29.8521	6	1	2012	2.00	4
3	37.9589	3	0	2012	2.00	2
2	26.3041	6	1	2012	2.00	2
3	45.4274	3	0	2012	1.00	0
2	34.1973	3	0	2012	1.00	0
3	51.8630	2	0	2012	1.00	0
2	32.2849	2	0	2012	1.00	0
2	48.3644	2	0	2012	2.00	5
2	34.9370	6	1	2012	2.00	3
2	27.3260	6	1	2012	1.00	0
2	26.4247	2	0	2012	2.00	2
2	25.9507	2	0	2012	1.00	0
2	32.2274	2	0	2012	1.00	0
3	47.6521	6	1	2012	1.00	0
2	46.6219	6	1	2012	1.00	0
2	28.3233	6	1	2012	1.00	0
2	29.1452	6	1	2012	1.00	0
3	45.2219	6	1	2012	2.00	2
2	40.8411	6	1	2012	2.00	3
2	27.9014	6	1	2012	2.00	2
2	61.2438	6	1	2012	1.00	0
3	38.5068	6	1	2012	1.00	0
2	29.4740	6	1	2012	2.00	10
2	42.7534	6	1	2012	1.00	0
2	46.4521	6	1	2012	1.00	0
2	72.9836	6	1	2012	1.00	0
2	37.4493	6	1	2012	1.00	0
3	58.0027	6	1	2012	2.00	3
2	29.3479	6	1	2012	2.00	5
2	62.1534	6	1	2012	2.00	2
2	44.2466	6	1	2012	2.00	2
2	30.5671	6	1	2012	1.00	0
2	19.2384	6	1	2012	1.00	0
2	34.5534	6	1	2012	2.00	3
3	32.6082	6	1	2012	1.00	0
3	20.6767	6	1	2012	1.00	0
2	31.8192	6	1	2012	1.00	0
2	21.0767	6	1	2012	1.00	0
2	45.7589	3	0	2012	1.00	0
3	17.4082	2	0	2012	1.00	0
2	36.6219	3	0	2012	1.00	0
2	24.6329	6	1	2012	1.00	0
2	33.8849	6	1	2012	1.00	0
2	36.8164	2	0	2012	1.00	0
3	25.7205	2	0	2012	1.00	0
2	30.2521	2	0	2012	1.00	0
2	35.9178	2	0	2012	1.00	0
2	20.6000	2	0	2012	1.00	0
3	49.9315	2	0	2012	1.00	0
2	45.3233	4	0	2012	1.00	0
2	26.9178	6	1	2012	1.00	0
3	19.5397	3	0	2012	1.00	0
2	38.4301	5	0	2012	2.00	2
2	42.2137	6	1	2012	1.00	0
3	50.5973	6	1	2012	1.00	0
2	32.7890	6	1	2012	1.00	0
3	30.3288	2	0	2012	1.00	0
3	34.8137	6	1	2012	1.00	0
3	34.3781	2	0	2012	1.00	0
2	34.6493	2	0	2012	1.00	0
2	18.3836	6	1	2012	1.00	0
2	31.2877	6	1	2012	1.00	0
3	36.5836	2	0	2012	1.00	0
2	18.2877	5	0	2012	1.00	0
2	28.1205	2	0	2012	1.00	0
2	38.9370	2	0	2012	1.00	0
2	70.1260	2	0	2012	1.00	0
2	43.5288	3	0	2012	1.00	0
2	19.3753	6	1	2012	1.00	0
2	34.5315	6	1	2012	1.00	0

2	37.1233	6	1	2012	1.00	0
2	55.9342	2	0	2012	1.00	0
2	26.4301	2	0	2012	1.00	0
2	41.9753	3	0	2012	2.00	3
2	33.2603	6	1	2012	1.00	0
2	30.7644	2	0	2012	1.00	0
2	37.1945	3	0	2012	1.00	0
2	61.8521	6	1	2012	2.00	2
2	35.6849	3	0	2012	2.00	4
2	48.4192	4	0	2012	1.00	0
3	37.2521	6	1	2012	1.00	0
2	37.3452	6	1	2012	1.00	0
2	42.0548	3	0	2012	1.00	0
3	41.1151	2	0	2013	2.00	4
2	37.9397	3	0	2013	2.00	2
2	27.7178	6	1	2013	2.00	2
2	38.3342	2	0	2013	1.00	0
3	47.5315	2	0	2013	1.00	0
3	68.8849	2	0	2013	2.00	2
3	48.4767	3	0	2013	1.00	0
3	26.1041	3	0	2013	1.00	0
3	53.9918	3	0	2013	1.00	0
2	52.2000	3	0	2013	2.00	24
2	45.4411	6	1	2013	1.00	0
2	51.5973	6	1	2013	2.00	2
3	49.8822	2	0	2013	1.00	0
2	25.8658	2	0	2013	2.00	2
2	34.2027	2	0	2013	2.00	2
2	27.0658	4	0	2013	2.00	2
2	36.1288	6	1	2013	1.00	0
3	17.4137	2	0	2013	1.00	0
2	48.2493	6	1	2013	1.00	0
2	24.4575	2	0	2013	2.00	3
3	43.3671	2	0	2013	2.00	3
3	23.1178	6	1	2013	2.00	5
2	28.6849	6	1	2013	2.00	2
2	49.2274	6	1	2013	1.00	0
2	27.5370	6	1	2013	2.00	2
2	45.6438	2	0	2013	2.00	2
2	16.6521	6	1	2013	1.00	0
2	43.9534	3	0	2013	2.00	2
2	25.7151	2	0	2013	2.00	4
3	42.9753	2	0	2013	1.00	0
2	54.1589	2	0	2013	2.00	3
2	50.9479	3	0	2013	1.00	0
2	40.4658	2	0	2013	1.00	0
2	45.3452	6	1	2013	1.00	0
3	29.6000	6	1	2013	1.00	0
2	42.3342	3	0	2013	1.00	0
3	30.9781	2	0	2013	1.00	0
2	36.1562	3	0	2013	2.00	4
2	20.6849	6	1	2013	1.00	0
3	39.4658	6	1	2013	1.00	0
2	29.7342	2	0	2013	2.00	5
2	52.5699	3	0	2013	2.00	24
3	28.5589	3	0	2013	2.00	3
2	52.7425	3	0	2013	2.00	24
2	58.3863	3	0	2013	2.00	3
2	24.4110	2	0	2013	2.00	8
2	50.1863	3	0	2013	1.00	0
2	25.9945	2	0	2013	1.00	0
3	20.3836	6	1	2013	2.00	2
2	58.0603	3	0	2013	2.00	3
2	37.4301	3	0	2013	2.00	3
2	28.8493	6	1	2013	1.00	0
2	43.6438	5	0	2013	2.00	2
3	54.5808	6	1	2013	1.00	0
3	24.1863	2	0	2013	2.00	3
2	31.6795	3	0	2013	2.00	4
2	29.4740	6	1	2013	2.00	4
2	53.4055	2	0	2013	2.00	2
3	68.9589	2	0	2013	1.00	0
2	31.8822	3	0	2013	2.00	4
2	34.5753	6	1	2013	1.00	0
3	27.3863	2	0	2013	1.00	0
2	30.6411	6	1	2013	2.00	3
3	32.1342	2	0	2013	1.00	0

2	55.3123	2	0	2013	2.00	3
2	53.2712	2	0	2013	2.00	2
3	41.3918	5	0	2013	2.00	3
2	26.5479	2	0	2013	2.00	8
2	25.8795	2	0	2013	2.00	4
2	20.9151	6	1	2013	2.00	15
2	44.0740	5	0	2013	1.00	0
2	25.9644	2	0	2013	2.00	8
2	52.3836	3	0	2013	2.00	24
2	63.2493	3	0	2013	1.00	0
3	23.7151	6	1	2013	1.00	0
2	21.5836	6	1	2013	2.00	15
3	42.1041	2	0	2013	2.00	2
2	39.6082	2	0	2013	2.00	2
2	23.9151	2	0	2013	1.00	0
2	21.0000	6	1	2013	2.00	15
2	41.0986	5	0	2013	2.00	3
3	39.1918	2	0	2013	1.00	0
2	30.3041	2	0	2013	1.00	0
3	46.9753	6	1	2013	1.00	0
3	37.5315	6	1	2013	2.00	2
2	37.6438	5	0	2013	2.00	2
2	33.9753	2	0	2013	1.00	0
2	42.4548	2	0	2013	1.00	0
3	37.4767	2	0	2013	2.00	2
3	22.3808	2	0	2013	1.00	0
2	49.7233	3	0	2013	2.00	3
2	46.1589	3	0	2013	2.00	3
2	30.7315	6	1	2013	2.00	10
2	25.4795	6	1	2013	1.00	0
3	22.1479	6	1	2013	2.00	5
2	20.6740	6	1	2013	2.00	15
2	31.8685	3	0	2013	1.00	0
3	22.4740	6	1	2013	2.00	5
3	44.6438	3	0	2013	1.00	0
3	34.0603	6	1	2013	1.00	0
3	43.3014	2	0	2013	2.00	3
2	29.7616	6	1	2013	2.00	5
3	19.4493	6	1	2013	2.00	2
2	55.7452	2	0	2013	1.00	0
2	26.4795	6	1	2013	2.00	3
2	21.3397	6	1	2013	2.00	15
2	52.5616	3	0	2013	2.00	24
2	41.4110	3	0	2013	2.00	4
2	28.1397	5	0	2013	1.00	0
3	42.6932	2	0	2013	2.00	3
3	27.3973	6	1	2013	2.00	2
3	16.0959	6	1	2013	1.00	0
3	57.0055	2	0	2013	1.00	0
2	30.6548	6	1	2013	1.00	0
3	31.4822	2	0	2013	2.00	2
2	16.9753	2	0	2013	2.00	2
2	19.1452	2	0	2013	1.00	0
3	44.3753	2	0	2013	1.00	0
2	35.9616	2	0	2013	2.00	2
2	52.4000	3	0	2013	2.00	24
2	40.2301	6	1	2013	1.00	0
2	28.2712	6	1	2013	2.00	2
2	41.4164	3	0	2013	2.00	4
2	29.3918	6	1	2013	1.00	0
2	33.3068	6	1	2013	2.00	3
2	19.6438	6	1	2013	1.00	0
2	46.8274	3	0	2013	2.00	4
3	21.3507	3	0	2013	1.00	0
3	16.6438	3	0	2013	1.00	0
3	41.0219	3	0	2013	1.00	0
2	40.8959	2	0	2013	1.00	0
2	30.1644	6	1	2013	2.00	3
3	27.7479	6	1	2013	1.00	0
2	26.3233	6	1	2013	2.00	3
2	21.1753	6	1	2013	1.00	0
2	52.6521	3	0	2013	2.00	24
2	25.1945	2	0	2013	1.00	0
2	35.1589	2	0	2013	1.00	0
2	45.5123	3	0	2013	2.00	3
2	29.4192	5	0	2013	2.00	3
2	42.3973	5	0	2013	2.00	7

2	52.6548	3	0	2013	2.00	24
3	18.1178	3	0	2013	2.00	2
3	45.5753	2	0	2013	1.00	0
2	24.9068	2	0	2013	2.00	8
2	38.1397	2	0	2013	2.00	2
2	30.3315	3	0	2013	1.00	0
2	81.6712	3	0	2013	1.00	0
2	27.2356	2	0	2013	2.00	4
2	29.8247	2	0	2013	2.00	2
2	36.1644	2	0	2013	1.00	0
2	32.4904	2	0	2013	2.00	2
3	22.4904	6	1	2013	2.00	5
2	40.0329	2	0	2013	1.00	0
2	23.8795	2	0	2013	1.00	0
3	32.1616	2	0	2013	1.00	0
2	27.2329	6	1	2013	2.00	2
2	31.6274	3	0	2013	2.00	2
2	39.8192	5	0	2013	1.00	0
2	30.9973	6	1	2013	2.00	10
3	22.4110	6	1	2013	2.00	5
2	29.8274	2	0	2013	2.00	2
2	32.9808	6	1	2013	2.00	3
2	21.8932	2	0	2013	1.00	0
2	51.3452	6	1	2013	2.00	3
3	38.7507	2	0	2013	2.00	2
3	41.6685	2	0	2013	2.00	4
2	38.7507	3	0	2013	2.00	2
2	37.5068	3	0	2013	2.00	3
2	25.0795	2	0	2013	2.00	2
2	43.7096	2	0	2013	1.00	0
2	36.6521	4	0	2013	2.00	3
2	40.1836	3	0	2013	1.00	0
2	30.9178	6	1	2013	2.00	10
2	30.3644	6	1	2013	2.00	5
2	52.7479	3	0	2013	2.00	24
3	26.3616	3	0	2013	2.00	2
2	33.6466	2	0	2013	2.00	3
2	45.1808	2	0	2013	1.00	0
2	22.4110	6	1	2013	1.00	0
2	19.7260	6	1	2013	1.00	0
2	55.6849	3	0	2013	2.00	3
2	49.7507	3	0	2013	2.00	3
3	40.1753	2	0	2013	2.00	2
2	18.5041	2	0	2013	1.00	0
2	33.5699	6	1	2013	2.00	3
2	18.3534	6	1	2013	1.00	0
2	28.1205	6	1	2013	1.00	0
3	20.4274	2	0	2013	2.00	2
3	23.6411	3	0	2013	1.00	0
3	34.8658	3	0	2013	2.00	2
2	27.0466	2	0	2013	1.00	0
3	17.5562	3	0	2013	1.00	0
3	23.4110	6	1	2013	2.00	5
2	28.7096	2	0	2013	1.00	0
2	55.1068	3	0	2013	1.00	0
2	28.3123	6	1	2013	2.00	2
3	19.6986	2	0	2013	1.00	0
2	47.7178	2	0	2013	2.00	2
3	21.4137	2	0	2013	1.00	0
3	49.3890	3	0	2013	2.00	2
2	24.5890	2	0	2013	2.00	3
2	16.5096	3	0	2013	1.00	0
2	32.7425	2	0	2013	2.00	3
2	27.2521	2	0	2013	2.00	4
3	33.3178	3	0	2013	1.00	0
2	21.0301	6	1	2013	2.00	15
2	35.8137	2	0	2013	1.00	0
2	27.3836	6	1	2013	2.00	2
2	30.7507	4	0	2013	1.00	0
3	27.4219	6	1	2013	2.00	2
3	42.3644	3	0	2013	2.00	2
2	25.9233	2	0	2013	2.00	2
2	28.3425	2	0	2013	2.00	3
2	27.5068	2	0	2013	2.00	4
2	58.6384	3	0	2013	2.00	2
2	42.8521	2	0	2013	2.00	2
2	43.4411	2	0	2013	2.00	2

2	33.2055	3	0	2013	2.00	3
2	41.4740	5	0	2013	2.00	3
3	26.6247	3	0	2013	2.00	2
3	24.9068	3	0	2013	2.00	3
2	37.5178	5	0	2013	2.00	2
3	28.9973	2	0	2013	1.00	0
3	20.9123	3	0	2013	1.00	0
2	59.3808	3	0	2013	1.00	0
2	17.1671	2	0	2013	2.00	2
2	62.0110	6	1	2013	2.00	3
2	43.3397	3	0	2013	2.00	2
2	21.0356	6	1	2013	2.00	15
2	37.5205	2	0	2013	2.00	4
2	52.1863	3	0	2013	2.00	24
3	68.7918	2	0	2013	2.00	2
3	27.1507	3	0	2013	2.00	2
2	24.0658	3	0	2013	1.00	0
2	60.5397	2	0	2013	2.00	2
2	58.7452	3	0	2013	2.00	3
2	31.4247	2	0	2013	1.00	0
3	31.3890	2	0	2013	1.00	0
3	45.6110	6	1	2013	2.00	2
2	53.7260	3	0	2013	2.00	3
3	23.0055	6	1	2013	2.00	5
2	33.6712	2	0	2014	2.00	3
2	33.5507	2	0	2014	2.00	4
2	51.8082	3	0	2014	2.00	3
2	26.3479	2	0	2014	2.00	2
2	20.3041	6	1	2014	1.00	0
2	53.5205	3	0	2014	2.00	24
3	60.1699	6	1	2014	1.00	0
2	27.7836	4	0	2014	1.00	0
2	32.6082	6	1	2014	1.00	0
3	34.9479	2	0	2014	2.00	2
2	33.2438	3	0	2014	2.00	3
2	51.2849	6	1	2014	1.00	0
2	29.0877	6	1	2014	1.00	0
2	51.2959	2	0	2014	1.00	0
2	27.8466	2	0	2014	1.00	0
3	52.9507	3	0	2014	1.00	0
2	26.2795	2	0	2014	2.00	4
2	53.2356	3	0	2014	1.00	0
2	27.7233	6	1	2014	1.00	0
2	78.7370	6	1	2014	2.00	5
3	45.3808	6	1	2014	2.00	2
3	25.4548	6	1	2014	1.00	0
2	34.6301	2	0	2014	1.00	0
3	42.3068	2	0	2014	2.00	4
2	19.4877	2	0	2014	2.00	3
2	28.1836	2	0	2014	2.00	3
2	57.8274	2	0	2014	1.00	0
2	53.5589	3	0	2014	2.00	24
2	45.8137	2	0	2014	1.00	0
3	24.4055	2	0	2014	2.00	3
2	66.3233	6	1	2014	1.00	0
2	32.5479	2	0	2014	2.00	2
2	46.7479	3	0	2014	2.00	3
2	35.6356	2	0	2014	2.00	2
2	27.0658	2	0	2014	2.00	8
3	21.5425	6	1	2014	1.00	0
2	32.2932	2	0	2014	1.00	0
2	54.0959	3	0	2014	1.00	0
2	50.9205	2	0	2014	2.00	3
2	24.2055	6	1	2014	1.00	0
2	21.3945	6	1	2014	1.00	0
2	45.7753	3	0	2014	2.00	3
2	25.4082	2	0	2014	2.00	8
2	36.7288	2	0	2014	2.00	2
2	45.9425	3	0	2014	2.00	3
2	53.4137	3	0	2014	2.00	24
2	31.0575	6	1	2014	2.00	10
3	35.5863	2	0	2014	1.00	0
2	43.0110	2	0	2014	2.00	6
2	28.3753	2	0	2014	2.00	3
3	44.6767	5	0	2014	1.00	0
3	25.5288	6	1	2014	2.00	2
2	27.2603	2	0	2014	2.00	8

2	28.1863	5	0	2014	1.00	0
2	53.5123	3	0	2014	2.00	24
2	32.0082	6	1	2014	2.00	4
2	51.8904	3	0	2014	2.00	3
2	19.6493	2	0	2014	2.00	3
2	31.0630	6	1	2014	2.00	10
2	35.4055	6	1	2014	1.00	0
2	38.2137	6	1	2014	1.00	0
3	38.0247	6	1	2014	2.00	2
2	44.3342	5	0	2014	1.00	0
2	71.2055	5	0	2014	2.00	5
2	21.8329	6	1	2014	2.00	15
3	42.4027	6	1	2014	1.00	0
2	24.2548	6	1	2014	1.00	0
2	40.5479	2	0	2014	1.00	0
2	26.7616	6	1	2014	1.00	0
3	59.4137	6	1	2014	2.00	3
3	47.5616	6	1	2014	1.00	0
2	59.3973	3	0	2014	1.00	0
2	56.3397	3	0	2014	2.00	2
2	43.4795	2	0	2014	2.00	3
2	31.7260	2	0	2014	1.00	0
2	43.3151	2	0	2014	2.00	2
2	78.5973	6	1	2014	2.00	5
2	19.0548	2	0	2014	2.00	2
2	33.7534	2	0	2014	2.00	4
2	29.7397	2	0	2014	2.00	3
2	33.2493	6	1	2014	1.00	0
3	46.6658	2	0	2014	1.00	0
3	26.0000	5	0	2014	1.00	0
2	18.2849	5	0	2014	1.00	0
2	33.8986	6	1	2014	1.00	0
2	37.7397	3	0	2014	2.00	3
2	26.3096	2	0	2014	2.00	2
2	23.2274	2	0	2014	1.00	0
2	20.9205	3	0	2014	1.00	0
3	27.7863	3	0	2014	1.00	0
3	45.1918	6	1	2014	1.00	0
2	56.2466	2	0	2014	2.00	3
2	25.5918	6	1	2014	1.00	0
3	38.8027	2	0	2014	1.00	0
2	39.3233	5	0	2014	1.00	0
3	46.8329	2	0	2014	2.00	4
2	71.1397	5	0	2014	2.00	5
2	22.9068	2	0	2014	1.00	0
2	27.4384	6	1	2014	2.00	3
2	39.6548	6	1	2014	1.00	0
3	16.8082	2	0	2014	2.00	2
2	24.8247	2	0	2014	2.00	2
2	38.4685	2	0	2014	2.00	2
2	37.9863	5	0	2014	2.00	2
3	30.8137	2	0	2014	1.00	0
2	70.8164	5	0	2014	2.00	5
3	20.0493	6	1	2014	2.00	2
2	39.5616	6	1	2014	1.00	0
3	25.3014	3	0	2014	2.00	3
2	57.9260	2	0	2014	1.00	0
3	42.0822	5	0	2014	2.00	3
3	33.9836	6	1	2014	1.00	0
2	44.6630	2	0	2014	1.00	0
2	35.0685	2	0	2014	1.00	0
2	29.6685	2	0	2014	2.00	3
2	43.1644	2	0	2014	2.00	6
2	27.4932	2	0	2014	1.00	0
2	32.9342	2	0	2014	1.00	0
3	42.0849	5	0	2014	2.00	3
2	47.9425	4	0	2014	1.00	0
2	78.7808	6	1	2014	2.00	5
2	41.5452	5	0	2014	2.00	3
3	25.1397	3	0	2014	2.00	3
2	41.5370	2	0	2014	1.00	0
2	28.1479	3	0	2014	1.00	0
2	39.0904	6	1	2014	1.00	0
3	33.4027	3	0	2014	1.00	0
2	29.6822	6	1	2014	2.00	2
2	35.4466	3	0	2014	1.00	0
2	44.4027	3	0	2014	2.00	2

3	46.8466	2	0	2014	2.00	4
3	34.7452	6	1	2014	2.00	2
2	56.3589	3	0	2014	2.00	3
3	16.7397	2	0	2014	2.00	2
2	33.7041	2	0	2014	2.00	4
3	18.1096	2	0	2014	1.00	0
3	38.4247	2	0	2014	2.00	2
3	30.6712	2	0	2014	1.00	0
2	42.7342	3	0	2014	2.00	2
2	78.7890	6	1	2014	2.00	5
2	20.0877	6	1	2014	1.00	0
2	21.7014	6	1	2014	2.00	15
2	28.2521	2	0	2014	2.00	4
3	32.2877	3	0	2014	1.00	0
3	40.0438	3	0	2014	1.00	0
3	18.4740	2	0	2014	1.00	0
2	31.8904	2	0	2014	1.00	0
2	20.8438	2	0	2014	2.00	2
3	25.4219	6	1	2014	2.00	3
3	34.5425	3	0	2014	1.00	0
3	23.6247	2	0	2014	1.00	0
2	30.8740	6	1	2014	2.00	5
3	66.9589	6	1	2014	1.00	0
2	39.4575	6	1	2014	1.00	0
2	30.7918	6	1	2014	2.00	5
2	29.6904	2	0	2014	2.00	3
2	44.3973	5	0	2014	1.00	0
2	45.0247	2	0	2014	1.00	0
2	45.0247	2	0	2014	1.00	0
2	38.2164	3	0	2014	1.00	0
3	70.0384	6	1	2014	1.00	0
3	43.5699	2	0	2014	2.00	3
3	26.9863	2	0	2014	1.00	0
3	38.2575	3	0	2014	1.00	0

Walk_In	Fach	Subspez	Diagnose	Suizidalität	Gewalt	AnzahlCo_Morbiditäten
1	2	11	98	0	0	0
1	2	3	50	0	0	0
1	5	9	139	0	0	0
1	5	9	139	0	0	0
1	5	12	16	0	0	8
1	2	3	19	0	0	0
1	2	3	19	0	0	0
1	6	15	9	0	0	1
1	2	3	49	0	0	2
1	2	3	19	0	0	0
1	2	3	19	0	0	0
0	2	11	48	0	0	4
1	5	2	6	0	0	1
1	2	3	19	0	0	0
1	6	15	47	1	0	2
1	5	10	70	0	0	5
1	6	15	87	0	0	0
1	2	11	48	0	0	0
1	5	6	31	0	0	0
1	5	9	139	0	0	0
1	6	15	132	0	0	0
0	5	6	31	0	0	1
1	2	3	50	0	0	0
1	6	15	3	1	0	0
1	5	6	31	0	0	3
1	2	11	177	0	0	0
1	5	6	31	0	0	0
1	5	9	139	0	0	0
1	2	11	98	0	0	2
1	2	3	19	0	0	3
1	5	9	139	0	0	8
1	5	6	31	0	0	0
1	5	9	24	0	0	9
1	2	3	19	0	0	1
1	6	15	11	0	0	7
1	5	14	20	0	0	1
1	2	3	49	0	0	0
1	5	10	21	0	0	10
1	2	3	157	0	0	0
1	2	11	98	0	0	0
1	5	12	16	0	0	1
1	5	12	81	0	0	1
1	5	9	139	0	0	0
0	2	11	52	0	0	0
1	2	11	98	0	0	1
1	5	12	28	0	0	0
1	5	9	53	0	0	1
1	5	9	53	0	0	2
1	5	2	6	0	0	0
1	6	15	38	0	0	1
1	2	3	49	0	0	0
0	2	11	54	0	0	3
1	2	3	19	0	0	0
1	2	3	19	0	0	0
0	5	5	69	0	0	0
0	5	9	139	0	0	0
1	5	12	16	0	0	1
1	3	4	178	0	0	0
1	2	3	50	0	0	0
1	5	9	176	0	0	0
1	5	9	139	0	0	0
1	2	11	104	0	0	1
1	6	15	9	0	0	0
1	5	14	20	0	0	1
1	5	9	139	0	0	5
1	2	3	49	0	1	0
1	2	3	19	0	1	0
1	2	3	49	0	0	1
1	5	6	75	0	0	2
1	6	15	5	0	0	0
1	5	12	16	0	0	0
1	2	11	177	0	0	0
1	2	11	26	0	0	1
1	6	15	72	0	0	1
1	6	15	64	1	0	5
1	5	12	155	0	0	2

1	2	11	98	0	0	1
1	5	9	139	0	0	0
1	5	9	53	0	0	3
1	5	9	139	0	0	0
1	2	11	48	0	0	0
1	2	3	49	0	1	1
1	5	14	138	0	0	0
1	2	11	98	0	0	0
1	2	3	19	0	0	0
1	5	6	31	0	0	4
1	2	3	19	0	0	1
1	6	15	106	0	0	1
1	2	3	19	0	0	0
1	5	9	139	0	0	0
1	5	9	139	0	0	0
1	2	11	173	0	0	1
1	5	9	139	0	0	0
1	2	3	19	0	0	0
1	5	12	155	0	0	0
1	5	6	31	0	0	0
1	2	3	49	0	1	0
1	2	3	19	0	0	1
1	6	15	136	0	0	1
1	5	12	28	0	0	0
1	5	6	175	0	0	0
1	6	15	2	0	0	1
1	3	4	44	0	0	4
1	5	16	94	0	0	0
1	5	9	139	0	0	5
1	2	3	19	0	0	3
1	5	6	31	0	0	0
1	5	9	139	0	0	0
1	5	6	31	0	0	0
1	2	11	18	0	0	5
1	5	12	16	0	0	7
1	2	3	19	0	0	0
1	2	11	84	0	0	5
1	2	3	50	0	0	0
1	2	3	19	0	0	0
1	5	9	139	0	0	0
1	2	3	19	0	0	0
1	5	12	28	0	0	5
1	4	8	167	0	0	0
1	2	3	19	0	0	0
1	2	11	101	0	0	5
1	2	3	50	0	1	0
1	5	12	16	0	0	0
1	5	12	16	0	0	7
1	2	11	101	0	0	1
1	2	11	101	0	0	6
1	2	3	49	0	0	3
1	6	15	111	0	0	0
1	2	3	19	0	1	0
1	2	3	157	0	0	0
1	5	10	71	0	0	6
1	6	15	2	0	0	1
1	4	8	141	0	0	1
1	2	11	101	0	0	1
1	2	11	91	0	0	7
1	6	15	161	1	0	0
1	2	3	19	0	0	0
1	6	15	169	1	0	1
1	5	9	139	0	0	0
1	6	15	161	1	1	0
1	2	3	19	0	0	0
1	5	6	31	0	0	0
1	2	3	49	0	0	0
1	2	3	19	0	0	0
1	2	11	173	0	0	0
1	2	11	182	0	0	0
1	5	9	139	0	0	0
1	6	15	2	1	0	4
1	2	3	49	0	0	0
1	5	6	31	0	0	0
1	2	3	49	0	1	1
1	2	3	19	0	0	0
1	2	3	49	0	0	0

1	6	15	161	1	0	1
1	2	11	107	0	0	1
1	2	7	30	0	0	0
1	2	3	19	0	0	0
1	5	6	31	0	0	0
1	6	15	160	0	0	7
1	2	11	101	0	0	1
1	6	15	143	0	0	0
1	6	15	130	1	0	4
1	2	11	101	0	0	0
1	6	15	161	1	0	1
1	2	3	19	0	0	0
1	2	11	105	0	0	0
1	6	15	87	1	0	3
1	5	12	16	0	0	1
1	2	3	19	0	0	0
1	6	15	128	0	0	3
1	6	15	22	0	0	0
1	2	11	101	0	0	0
1	2	11	91	0	0	0
1	2	3	49	0	0	1
1	2	11	101	0	0	0
1	2	3	19	0	0	1
1	5	6	31	0	0	1
1	4	8	40	0	0	3
1	4	8	110	0	0	0
1	6	15	161	1	0	1
1	5	10	32	0	0	1
1	5	9	24	0	0	2
1	2	11	91	0	0	0
1	2	11	172	0	0	5
1	2	3	49	0	0	0
1	5	9	139	0	0	0
1	5	12	16	0	0	1
1	2	11	101	0	0	8
1	5	12	16	0	0	3
1	6	15	161	1	0	2
1	2	3	50	0	0	0
1	5	9	139	0	0	1
1	2	3	19	0	1	1
1	2	3	19	0	0	0
1	2	3	19	0	0	1
1	2	11	101	0	0	0
1	2	3	19	0	0	0
1	2	3	49	0	0	0
1	2	11	173	0	0	0
1	4	8	93	0	0	0
1	3	4	44	0	0	1
1	6	15	38	0	0	4
1	5	9	139	0	0	1
1	6	15	22	1	0	0
1	5	6	31	0	0	2
1	6	15	143	0	0	0
1	2	3	19	0	1	0
1	2	3	19	0	0	0
1	5	2	6	0	0	0
1	2	3	49	0	0	0
1	2	3	49	0	0	0
1	2	3	49	0	0	0
1	5	9	139	0	0	0
1	2	11	104	0	0	3
1	2	3	19	0	1	0
1	2	11	173	0	0	2
1	2	3	49	0	0	0
1	6	15	143	0	0	0
1	2	11	78	0	0	0
1	5	9	53	0	0	0
1	5	9	53	0	0	1
1	5	12	155	0	0	5
1	2	3	50	0	0	0
1	6	15	5	0	0	2
1	2	11	104	0	0	1
1	5	12	16	0	0	0
1	2	11	91	0	0	0
1	2	11	164	0	0	0
1	2	11	76	0	0	2
1	6	15	134	0	0	0

1	3	4	44	0	0	1
1	2	11	177	0	0	0
1	2	3	19	0	0	0
1	5	9	139	0	0	1
1	2	11	37	0	0	1
1	2	11	29	0	0	0
1	2	11	91	0	0	0
1	2	11	116	0	0	0
1	2	11	91	0	0	3
1	5	9	139	0	0	0
1	2	3	49	0	1	0
1	2	3	49	0	0	0
1	2	11	129	0	0	0
1	5	12	16	0	0	0
1	2	3	50	0	0	0
1	6	15	34	0	0	2
1	6	15	38	0	0	1
1	5	12	16	0	0	3
1	2	11	91	0	0	0
1	6	15	3	1	0	1
1	2	3	157	0	0	0
1	5	6	31	0	0	0
1	5	6	31	0	0	0
1	5	10	166	0	0	0
1	5	6	31	0	0	2
1	2	3	19	0	0	0
1	4	8	109	0	0	0
1	5	10	79	0	0	1
1	2	3	19	0	0	0
1	4	8	168	0	0	0
1	2	11	91	0	0	0
1	5	14	12	0	0	1
1	2	11	91	0	0	0
1	5	6	75	0	0	0
1	5	6	31	0	0	1
1	6	15	5	0	0	0
1	2	11	177	0	0	0
1	5	9	24	0	0	0
1	2	3	19	0	0	0
1	2	11	173	0	0	1
1	2	3	49	0	0	0
1	2	3	49	0	1	0
1	5	14	12	0	0	0
1	2	3	49	0	0	0
1	2	3	19	0	0	1
1	5	9	139	0	1	0
1	6	15	27	0	0	2
1	2	3	157	0	0	0
1	5	6	31	0	0	2
1	3	4	57	0	0	0
1	2	11	63	0	0	1
1	6	15	27	0	0	1
1	2	3	19	0	0	0
1	5	16	137	0	0	1
1	4	8	140	0	0	0
1	2	11	101	0	0	0
1	6	15	133	0	0	0
1	2	3	19	0	0	0
1	4	8	120	0	0	3
1	2	3	19	0	1	0
1	5	6	31	0	0	0
1	2	11	173	0	0	0
1	2	11	91	0	0	0
1	5	9	139	0	0	0
1	5	12	16	0	0	0
1	5	12	16	0	0	1
1	2	11	116	0	0	0
1	5	13	90	0	0	4
1	5	9	24	0	0	0
1	6	15	144	1	0	0
1	2	3	50	0	1	0
1	6	15	8	0	0	1
1	2	11	117	0	0	0
1	5	9	139	0	0	0
1	4	8	109	0	0	0
1	2	11	101	0	0	1
1	6	15	87		0	1

1	5	9	139	0	0	0
1	2	11	177	0	0	0
1	6	15	163	1	0	0
1	5	9	139	0	0	1
1	2	11	145	0	0	0
1	5	12	16	0	0	0
1	5	12	16	0	0	4
1	6	15	5	1	0	0
1	5	16	42	0	0	1
1	5	12	16	0	0	0
1	5	12	16	0	0	1
1	6	15	3	1	0	0
1	2	11	177	0	0	0
1	2	11	104	0	0	0
1	2	3	19	0	0	2
1	5	12	16	0	0	0
1	2	11	10	0	0	0
1	5	9	139	0	0	0
1	2	11	100	0	0	0
1	4	8	40	0	0	0
1	5	12	16	0	0	0
1	5	2	6	0	0	0
1	4	8	109	0	0	0
1	2	11	100	0	0	1
1	6	15	9	0	0	0
1	5	12	16	0	0	0
1	2	3	157	0	0	0
1	5	13	90	0	0	5
1	4	8	181	0	0	0
1	5	9	139	0	0	2
1	6	15	3	1	0	0
1	5	6	31	0	0	0
1	5	9	139	0	0	0
1	6	15	5	1	1	0
1	5	9	24	0	0	0
1	5	12	28	0	0	0
1	4	8	110	0	0	0
1	2	3	19	0	0	1
1	5	9	176	0	0	1
1	5	9	139	0	0	1
1	2	3	49	0	0	1
1	2	3	50	0	1	0
1	6	15	38	0	0	0
1	2	3	50	0	0	0
1	2	3	19	0	0	0
1	2	3	19	0	0	4
1	4	8	146	0	0	0
1	6	15	153	0	0	0
1	5	9	24	0	0	0
1	5	12	16	0	0	1
1	2	3	19	0	0	0
1	2	3	19	0	1	0
1	5	9	139	0	0	0
1	5	12	28	0	0	0
1	2	11	173	0	0	3
1	5	9	139	0	0	0
1	2	3	49	0	0	0
1	2	3	49	0	0	
1	6	15	163	1	0	0
1	2	3	49	0	0	0
1	2	3	19	0	0	1
1	2	3	19	0	0	0
1	5	9	53	0	0	0
1	5	12	155	0	0	1
1	6	6	31	0	0	1
1	2	11	126	0	0	0
1	2	11	177	0	0	0
1	2	11	108	0	0	0
1	2	11	48	0	0	1
1	2	11	184	0	0	0
1	6	15	72	0	0	0
1	5	6	31	0	0	2
1	6	15	134	0	0	1
1	2	3	19	0	0	0
1	5	6	31	0	0	2
1	2	11	48	0	0	0
1	5	9	139	0	0	0

1	5	9	24	0	0	0
1	6	15	135	0	0	0
1	5	10	32	0	0	4
1	2	3	19	0	0	0
1	2	3	19	0	0	0
1	2	11	26	0	0	0
1	2	11	26	0	0	0
1	5	13	90	0	0	1
1	2	11	173	0	0	0
1	2	3	19	0	0	0
1	5	12	180	0	0	1
1	5	9	139	0	0	0
1	6	15	87	0	0	3
1	2	3	19	0	1	0
1	5	12	16	0	0	5
1	5	9	24	0	0	0
1	2	11	100	0	0	0
1	5	2	6	0	0	0
1	4	8	170	0	1	0
1	6	15	118	1	0	0
1	5	16	56	0	0	1
1	5	12	16	0	0	7
1	5	10	162	0	0	0
1	6	15	5	1	0	1
1	2	11	173	0	0	3
1	2	11	98	0	0	1
1	2	11	124	0	0	0
1	2	3	19		0	2
1	2	3	19	0	1	0
1	2	11	48	0	0	0
0	5	9	139	0	0	0
1	6	15	5	0	0	1
1	2	11	185	0	0	1
1	6	15	95	0	1	2
1	4	8	89	0	0	0
1	2	3	49	0	0	0
1	5	13	90	0	0	8
1	5	14	142	0	0	6
1	2	3	49	0	1	0
1	5	10	127	0	0	0
1	2	3	19	0	0	0
1	5	9	176	0	0	0
1	2	17	177	0	0	1
1	6	15	174	1	0	3
1	6	15	3	1	0	2
1	2	3	19	0	0	1
1	5	9	53	0	0	0
1	2	3	19	0	0	0
1	3	4	159	0	0	1
1	5	9	139	0	0	2
0	2	11	55	0	0	6
1	4	8	109	0	0	0
1	2	3	19	0	1	0
1	2	11	58	0	0	3
1	6	15	67	1	0	0
1	2	3	19	0	1	2
0	5	9	139	0	0	0
1	6	15	3	1	0	0
1	5	9	53	0	0	0
1	5	12	180	0	0	1
1	2	11	98	0	0	1
1	3	4	44	0	0	0
1	2	11	173	0	0	0
0	5	9	139	0	0	0
1	5	6	31	0	0	1
1	2	11	98	0	0	0
1	5	2	6	0	0	0
1	5	2	6	0	0	0
1	2	11	123	0	0	0
1	4	8	114	0	0	1
1	2	3	49	0	1	0
1	2	11	51	0	0	0
1	5	6	31	0	0	3
1	5	9	139	0	0	3
1	2	11	177			2
1	6	15	72	0	0	4
1	6	10	149	0	0	0

0	2	3	49	0	0	0
1	6	15	80	1	0	5
1	5	6	25	0	0	0
1	5	9	139	0	0	0
1	5	12	16	0	0	0
1	2	11	63	0	0	0
1	5	9	139	0	0	0
0	5	9	176	0	0	8
0	5	9	139	0	0	0
1	5	9	139	0	0	1
0	2	11	97	0	0	0
1	2	3	19	0	0	0
1	5	9	139	0	0	0
1	2	11	98	0	0	2
0	2	11	98	0	0	0
1	2	11	177	0	0	0
1	2	3	19	0	0	0
1	2	3	49	0	0	0
1	5	10	162	0	0	1
1	6	15	73	1	0	2
1	5	6	31	0	0	0
1	5	12	180	0	0	0
1	2	17	129	0	0	9
1	2	11	59	0	0	0
1	5	10	70	0	0	2
1	6	15	9	0	0	0
1	2	11	63	0	0	0
1	3	4	35	0	0	1
1	6	15	47	1	0	2
1	2	3	19	0	0	0
1	2	3	19	0	0	0
1	2	3	50	0	0	0
1	2	11	125	0	0	0
1	5	12	180			6
1	5	9	139	0	0	1
1	2	11	98	0	0	0
1	5	9	139	0	0	4
1	2	11	86	0	0	0
1	5	14	12	0	0	1
1	6	15	72	0	0	0
1	2	3	19	0	1	0
1	6	15	118	1	0	1
1	5	10	166	0	0	2
1	6	15	3	1	0	1
1	5	9	53	0	0	0
1	2	11	15	0	0	0
0	5	6	14	0	0	3
1	5	9	139	0	0	0
1	6	15	3	1	0	2
1	2	3	19	0	1	0
1	2	3	19	0	0	0
1	6	15	72	0	0	1
1	6	15	3	1	0	0
1	5	10	166	0	0	2
1	2	3	19	0	0	10
1	5	9	139	0	0	0
1	5	9	139	0	0	5
1	6	15	87	0	0	6
1	2	3	49	0	0	0
0	2	11	115			3
1	2	3	19			0
1	2	11	19	0	1	4
0	2	11	151	0	0	5
1	2	11	102	0	0	5
1	2	11	33	0	0	0
1	3	4	44	0	0	5
1	5	5	69	0	0	3
1	5	9	176	0	0	1
0	2	11	119	0	0	0
1	2	11	86	0	0	0
1	5	5	69	0	0	1
1	2	11	173	0	0	0
1	2	11	158	0	0	0
1	2	11	45	0	0	0
1	5	12	180	0	0	1
1	2	3	19	0	1	0
1	5	9	139	0	0	0

1	2	3	19	0	0	5
1	3	4	44	0	0	3
0	5	12	16	0	0	1
1	5	10	166	0	0	0
1	6	15	173	1	0	3
1	2	3	19	0	0	1
1	2	11	39	0	0	0
1	5	9	139	0	0	1
1	5	10	65	0	0	6
1	5	9	53	0	0	1
1	6	15	72	0	0	3
1	5	12	16	0	0	0
1	5	9	53	0	0	7
1	5	9	139	0	0	0
1	2	3	19	0	0	1
1	2	3	19	0	0	0
1	5	9	24	0	0	0
1	5	12	180	0	0	0
1	6	15	36	0	0	1
1	5	9	139	0	0	0
1	5	2	6	0	0	0
1	5	9	53	0	0	0
0	2	3	19	0	0	0
1	5	9	53	0	0	0
1	5	6	31	0	0	0
1	2	3	19	0	0	0
1	2	11	91	0	0	2
1	2	11	104	0	0	0
1	5	9	176	0	0	0
1	5	9	139	0	0	0
1	2	3	19	0	0	0
1	5	12	16	0	0	0
1	5	9	53	0	0	0
1	6	15	23	1	0	2
1	2	11	18	0	0	0
1	2	11	98	0	0	0
0	5	12	81	0	0	0
1	4	8	113	0	0	0
1	2	7	68		0	1
1	2	11	98	0	0	3
1	2	11	173	0	0	0
1	2	3	19	0	0	0
1	5	9	139	0	0	9
1	2	3	19	0	1	2
0	2	11	52	0	0	1
1	5	9	53	0	0	0
1	2	11	173	0	0	0
1	2	3	19	0	0	0
1	5	9	139	0	0	2
1	5	9	139	0	0	0
0	5	9	139	0	0	1
0	2	11	119	0	0	1
1	2	11	48	0	0	0
1	5	9	139	0	0	0
1	2	11	91	0	0	1
1	6	15	132	1	1	8
1	5	9	139	0	0	0
1	2	3	19	0	0	0
1	2	3	157	0	1	1
1	2	11	158	0	0	0
1	3	4	178	0	0	0
1	2	3	19	0	0	0
1	5	9	53	0	0	0
1	5	6	31	0	0	1
1	2	3	19	0	0	0
1	5	6	31	0	0	1
1	2	3	50	0	0	1
1	6	15	147	1	0	1
1	2	11	98	0	0	0
1	2	11	98	0	0	0
1	6	15	4	1	0	1
1	5	12	16	0	0	9
1	2	11	7	0	0	2
1	2	3	19	0	0	0
1	5	9	139	0	0	0
1	5	10	166	0	0	0
1	5	9	139	0	0	1

1	2	11	171	0	0	9
1	5	9	24	0	0	0
1	6	15	162	0	0	0
1	2	3	50	0	0	0
1	5	12	16	0	0	3
1	2	11	129	0	0	0
0	5	9	139	0	0	10
1	2	3	50	0	1	0
1	2	3	157	0	0	2
1	2	11	13	0	0	4
1	5	9	139	0	0	0
1	2	3	49	0	0	0
1	5	12	180	0	0	0
1	2	3	49	0	0	0
1	5	9	176	0	0	2
1	2	11	173	0	0	0
1	5	9	53	0	0	2
1	2	3	49	0	0	0
1	6	15	87	0	0	1
1	2	3	49	0	0	0
1	2	3	157	0	0	2
1	2	3	49	0	0	0
1	2	11	63	0	0	0
1	2	11	122	0	0	0
1	5	9	139	0	0	0
1	5	10	166	0	0	2
1	2	11	103	0	0	0
1	5	10	166	0	0	0
1	5	9	139	0	0	0
1	5	10	162	0	0	0
1	2	11	121	0	0	0
1	2	11	33	0	0	1
1	6	15	87	0	0	0
1	5	9	139	0	0	2
0	5	10	82	0	0	10
1	6	15	163	1	0	0
1	5	5	74	0	0	3
1	5	10	70	0	0	1
1	5	2	6	0	0	0
1	2	3	49	0	0	0
1	5	6	31	0	0	2
1	2	11	26	0	0	1
1	5	10	166	0	0	0
1	2	3	19	0	0	0
1	2	11	148	0	0	0
1	6	15	72	0	0	0
1	5	6	31	0	0	1
1	2	11	99	0	0	0
1	2	11	52	0	0	0
1	5	9	176	0	0	0
1	2	11	98	0	0	0
1	6	15	43	1	0	2
1	2	11	173	0	0	9
1	5	12	81	0	0	1
1	2	11	91	0	0	2
1	2	11	91	0	0	0
1	6	15	127	0	0	0
1	2	11	98	0	0	0
1	5	12	16	0	0	0
1	2	11	26	0	0	0
1	2	3	19	0	0	1
1	3	4	183	0	0	0
1	2	3	49	0	0	1
1	2	11	125	0	0	0
1	4	8	109	0	0	0
1	2	3	19	0	1	0
1	2	3	50	0	0	0
1	2	11	98	0	0	0
1	5	9	139	0	0	1
1	5	9	24	0	0	0
1	6	15	131	0	0	4
1	2	3	50	0	1	0
0	2	3	50	0	1	0
1	2	3	49	0	0	0
1	2	3	157	0	0	2
1	6	15	22	0	0	1
1	5	6	14	0	0	1

1	6	15	9	0	0	7
1	2	3	19	0	0	0
1	5	9	139	0	0	0
1	2	11	151	0	0	0
1	4	8	109	0	0	0
1	5	9	53	0	0	1
1	5	9	139	0	0	0
0	5	10	165	0	0	0
1	5	9	24	0	0	0
1	5	10	165	0	0	2
1	6	15	36	0	0	0
1	2	3	19	0	0	0
1	2	11	48	0	0	0
1	2	11	98	0	0	7
1	2	11	98	0	0	8
1	6	15	22	0	0	0
1	6	15	132	0	0	0
0	2	11	98	0	0	7
1	5	6	31	0	0	7
1	3	4	77	0	0	0
1	2	7	150	0	0	1
0	5	12	81	0	0	6
1	6	15	22	0	0	0
0	6	15	160	1	0	1
1	5	12	155	0	0	1
1	2	11	100	0	0	0
1	5	16	56	0	0	0
1	5	9	53	0	0	0
1	5	9	139	0	0	0
1	5	10	21			5
1	2	11	98	0	0	1
1	6	15	147	1	0	0
1	2	3	49	0	1	0
1	5	9	139	0	0	0
1	5	9	139	0	0	0
1	5	9	53	0	0	3
1	5	9	24	0	0	2
1	5	6	31	0	0	0
1	2	3	49	0	1	0
1	5	12	16	0	0	0
1	5	9	24	0	0	1
1	2	3	157	0	0	0
1	2	3	19	0	0	0
1	2	17	186	0	0	3
1	5	6	31	0	0	3
0	2	3	157	0	0	1
1	2	3	50	0	1	0
1	5	10	162	0	0	3
1	5	9	24	0	0	0
1	5	12	16	0	0	0
1	5	12	16	0	0	1
1	5	10	88	0	0	0
1	2	3	49	0	1	0
1	2	3	19	0	0	0
1	5	10	83	0	0	3
0	6	15	22	1	0	1
1	6	15	72	0	0	1
1	2	11	98	0	0	0
1	6	15	9	1	0	0
1	2	3	157	0	0	2
1	6	15	47	1	0	4
1	2	11	116	0	0	1
1	2	11	91	0	0	6
1	6	15	3	1	0	1
1	5	9	176	0	0	0
1	5	12	16	0	0	1
1	2	3	49	0	1	0
1	2	11	48	0	0	3
1	6	15	87	0	0	1
1	5	10	82	0	0	9
1	6	15	87	0	0	1
1	5	9	139	0	0	0
1	5	6	31	0	0	7
1	5	12	16	0	0	1
1	2	11	61	0	0	0
1	6	15	85	1	0	3
1	5	6	31	0	0	0

1	2	3	49	0	0	0
1	5	14	92	0	0	6
1	5	12	16	0	0	0
1	5	16	56	0	0	1
1	3	4	41	0	0	0
1	5	10	166	0	0	2
1	2	3	50	0	0	0
1	6	15	11	1	0	1
1	2	11	91	0	0	1
1	2	11	98	0	0	1
0	5	12	28	0	0	5
1	5	9	139	0	0	0
1	6	15	112	0	0	0
1	2	3	49	0	0	0
1	5	12	16	0	0	3
1	2	3	49	0	0	0
1	5	9	139	0	0	0
1	2	11	173	0	0	0
0	5	9	176	0	0	3
1	2	3	49	0	0	0
1	2	3	49	0	0	0
1	2	3	49	0	0	0
1	6	15	3	1	0	2
1	5	9	176	0	0	4
1	6	15	133	0	0	0
1	5	9	139	0	0	0
1	6	15	3	1	0	2
1	2	11	173	0	0	1
1	6	15	87	0	0	4
0	6	15	153	0	0	0
1	2	3	19	0	0	0
1	2	3	50	0	0	0
1	6	15	3	1	0	4
1	3	4	178	0	0	0
1	2	3	50	0	0	0
1	5	9	139	0	0	0
1	5	9	139	0	0	0
1	5	6	31	0	0	0
1	3	4	178	0	0	0
1	2	11	66	0	0	0
1	5	9	139	0	0	0
1	4	8	40	0	0	0
1	2	11	52	0	0	3
1	2	11	152	0	0	3
1	6	15	3	1	0	0
1	2	3	50	0	0	0
1	5	9	53	0	0	0
0	5	6	31	0	0	0
1	5	9	53	0	0	6
1	2	3	50	0	0	2
1	2	3	19	0	0	0
1	6	15	87	0	0	7
1	4	8	109	0	0	0
1	5	12	16	0	0	3
1	2	11	7	0	0	0
1	5	14	20	0	0	3
1	5	9	53	0	0	5
1	5	12	16	0	0	0
1	5	12	180	0	0	1
1	5	9	139	0	0	0
0	5	12	81	0	0	2
1	2	11	98	0	0	2
1	5	9	139	0	0	7
1	5	12	28	0	0	1
1	2	11	60	0	0	0
1	5	12	16	0	0	0
1	2	3	19	0	0	8
1	5	9	139	0	0	2
1	2	3	19	0	0	1
1	5	9	139	0	0	0
1	4	8	17	0	0	0
1	5	14	62	0	0	1
1	6	15	22	1	0	0
1	6	15	87	0	0	0
1	6	15	87	0	0	0
1	2	3	49	0	0	0
1	6	15	87	0	0	0

1	2	11	7	0	0	3
1	2	11	91	0	0	0
0	6	15	156	0	0	1
1	5	6	31	0	0	0
1	5	9	139	0	0	0
0	5	9	139	0	0	2
1	2	11	173	0	0	1
1	2	3	19	0	0	0
1	2	3	19	0	0	1
1	2	11	179	0	0	1
1	5	6	175	0	0	0
1	5	12	16	0	0	0
1	6	15	5	1	1	0
1	2	11	46	0	0	0
1	6	15	3	1	0	2
1	2	3	50	0	0	0
1	5	6	14	0	0	0
1	2	11	173	0	0	0
0	2	11	173	0	0	4
0	6	15	154	0	0	0
1	5	9	139	0	0	0
1	5	12	16	0	0	1
1	5	9	139	0	0	1
0	6	15	3	1	0	0
1	6	15	149	0	0	1
1	6	15	163	1	0	2
1	2	3	19	0	0	1
1	6	15	96	1	1	1
1	5	12	155	0	0	2
1	2	3	19	0	0	0
1	2	11	91	0	0	3
1	6	15	87	0	0	2
1	6	15	67	1	0	0
1	6	15	67	0	0	0

CharlsonCo_MorbidityIndex	PsychischeGrunderkrankung	PsychGK_Typ
0	0	
0	0	
0	0	
0	0	
1	0	
0	0	
0	0	
0	1	7
0	0	
0	0	
0	0	
0	0	
1	0	
0	0	
0	1	5
1	1	4
0	1	9
0	0	
0	0	
0	0	
0	1	8
0	0	
0	0	
0	1	7
0	1	5
0	0	
0	0	
0	0	
0	0	
0	0	
2	0	
0	0	
0	1	7
0	1	4
1	1	5
1	0	
0	0	
1	1	8
0	0	
0	0	
0	1	7
0	1	7
0	0	
0	0	
0	0	
0	0	
0	0	
0	0	
0	0	
0	1	7
0	0	
5	0	
0	0	
0	0	
0	0	
0	0	
0	0	
0	0	
0	0	
0	0	
0	0	
0	0	
0	1	5
0	0	
0	0	
0	0	
0	1	4
0	0	
1	0	
0	1	4
0	1	5
0	0	
0	0	
0	1	9
5	1	8
0	1	4

0	1	5
0	0	
0	0	
0	0	
0	0	
0	1	5
0	0	
0	0	
0	0	
2	0	
2	0	
0	1	9
0	0	
0	0	
0	0	
0	0	
0	0	
0	0	
0	0	
0	0	
0	0	
0	1	9
0	1	6
0	0	
0	0	
1	1	6
0	0	
0	0	
1	1	5
0	0	
0	0	
0	0	
0	0	
1	0	
1	1	4
0	0	
0	0	
0	0	
0	0	
0	0	
0	0	
1	0	
0	0	
0	0	
0	0	
0	0	
0	0	
1	1	4
0	0	
0	0	
1	0	
0	1	7
0	0	
0	0	
2	0	
0	1	7
0	0	
0	0	
1	1	7
0	1	7
0	0	
0	1	5
0	0	
0	1	5
0	0	
1	0	
0	0	
0	0	
0	0	
0	0	
0	0	
1	1	7
0	0	
0	0	
0	0	
0	0	
0	0	

0	1	5
0	0	
0	0	
0	0	
0	0	
0	1	5
0	1	4
0	1	8
0	1	5
0	0	
0	1	8
0	0	
0	0	
0	1	9
0	1	6
0	0	
2	1	9
0	1	5
0	0	
0	0	
2	0	
0	0	
0	0	
0	0	
0	1	4
0	0	
0	1	8
0	0	
0	0	
0	0	
0	0	
0	0	
0	0	
0	0	
0	0	
0	1	6
0	1	6
0	0	
0	0	
0	0	
0	0	
0	0	
0	0	
0	0	
0	0	
0	0	
0	0	
0	0	
0	1	9
0	0	
0	1	5
0	0	
0	1	8
0	0	
0	0	
0	0	
0	0	
0	0	
0	0	
0	0	
0	0	
0	0	
0	0	
	0	
0	1	8
0	0	
0	0	
0	0	
0	0	
0	0	
0	1	8
0	0	
0	0	
0	0	
0	0	
1	0	
0	1	7

0	0	
0	0	
0	0	
0	0	
0	0	
0	0	
0	0	
0	0	
0	1	9
0	0	
0	0	
0	0	
0	0	
0	0	
0	0	
0	1	6
0	1	9
0	1	6
0	0	
0	1	7
0	0	
0	0	
0	0	
0	0	
0	1	5
0	0	
0	0	
1	0	
0	0	
0	0	
0	0	
0	0	
0	0	
0	0	
0	0	
0	1	4
0	0	
0	0	
0	0	
0	0	
0	0	
0	0	
0	0	
0	0	
0	0	
0	0	
0	0	8
0	0	
0	0	
0	0	
0	0	
0	1	8
0	0	
0	0	
0	0	
0	0	
0	1	8
0	0	
0	0	
0	0	
0	0	
0	0	
0	0	
0	0	
0	0	
0	0	
0	0	
2	0	
0	0	
0	1	7
0	0	
0	1	7
0	0	
0	0	
0	0	
0	0	
0	1	9

0	0	
0	0	
0	1	7
0	0	
0	0	
0	0	
1		
0	1	4
0	0	
0	0	
0	0	
0	1	7
0	0	
0	0	
0	1	5
0	0	
0	0	
0	0	
0	0	
0	0	
0	0	
0	0	
0	0	
0	0	
0	1	5
0	0	
0	0	
2	0	
0	0	
0	0	
0	1	5
0	0	
0	0	
0	1	4
0	0	
0	0	
0	0	
0	0	
0	0	
0	0	
1	0	
0	0	
0	1	6
0	0	
0	0	
1	0	
0	0	
0	1	6
0	0	
0	0	
0	0	
0	0	
0	0	
0	0	
0	0	
0	0	
0	0	
0	0	
0	1	7
0	0	
1	0	
0	0	
0	0	
0	1	7
1	1	6
0	0	
0	0	
0	0	
0	1	7
0	0	
0	1	6
0	1	9
0	1	7
0	0	
0	1	7
0	0	
0	0	

0	0	
0	1	8
1	1	7
0	0	
0	0	
0	0	
0	0	
2	0	
0	0	
0	0	
0	0	
0	0	
0	1	9
0	0	
1	0	
0	0	
0	0	
0	0	
0	0	
0	1	5
0	0	
1	0	
0	0	
0	1	5
0	1	6
0	0	
0	0	
0	0	
0	0	
0	0	
0	0	
0	1	4
0	0	
0	1	8
0	0	
0	0	
9	1	6
4	0	
0	0	
0	0	
0	0	
0	0	
0	0	
0	1	7
0	1	6
0	0	
0	0	
0	0	
0	0	
0	0	
2	0	
0	0	
0	0	
0	1	4
0	1	7
0	1	5
0	0	
0	1	5
0	0	
0	0	
0	0	
0	0	
0	0	
0	0	
0	0	
0	0	
0	0	
0	0	
0	0	
0	0	
0	0	
0	0	
0	1	4
1	0	
0	0	
1	1	8
0	0	

0	0	
1	1	7
0	0	
0	0	
0	0	
0	0	
0	0	
9	1	6
0	0	
0	0	
0	0	
0	0	
0	0	
0	0	
0	0	
0	0	
0	0	
0	0	
0	0	
0	1	6
0	0	
0	0	
1	1	6
0	0	
0	1	4
0	1	7
0	0	
0	0	
0	1	7
0	0	
0	0	
0	0	
0	0	
0	0	
0	0	
0	0	
0	0	
0	0	
0	0	
0	1	7
0	0	
0	1	7
0	1	5
0	1	5
0	0	
0	0	
1	0	
0	0	
0	1	5
0	0	
0	0	
0	1	7
0	1	7
0	0	
2	1	8
0	0	
0	1	9
0	1	6
0	0	
3	0	
0	0	
1	0	
0	1	5
1	1	7
0	0	
0	1	6
1	0	
0	0	
0	0	
0	0	
2	0	
0	0	
0	0	
0	0	
0	1	5
0	0	
0	0	

1	1	5
3	0	
0	1	5
0	1	7
0	1	7
0	0	
0	0	
0	0	
1	1	6
0	0	
0	1	7
0	0	
0	0	
0	0	
0	1	4
0	0	
0	0	
0	0	
0	1	4
0	0	
0	0	
0	0	
0	0	
0	0	
0	0	
0	0	
0	0	
0	0	
0	0	
0	0	
0	0	
0	0	
0	0	
0	1	6
0	0	
0	0	
0	0	
0	0	
0	0	
0	0	
0	0	
0	0	
2	1	8
0	0	
0	0	
0	0	
0	0	
0	0	
0	0	
0	0	
0	0	
0	0	
0	0	
0	0	
1	0	
2	1	8
0	0	
0	0	
0	0	
0	0	
0	0	
0	0	
0	0	
0	0	
0	0	
0	0	
0	0	
0	1	6
0	0	
0	0	
0	1	5
2	1	8
0	0	
0	0	
0	0	
0	0	
0	0	

2	1	8
0	0	
0	1	7
0	0	
0	0	
0	0	
2	0	
0	1	4
0	0	
0	1	7
0	0	
0	0	
0	0	
0	0	
0	0	
0	0	
0	0	
0	0	
0	1	9
0	0	
0	0	
0	0	
0	0	
0	0	
0	0	
0	1	7
0	0	
0	1	7
0	0	
0	0	
0	0	
1	0	
0	1	7
1	0	
2	1	8
0	1	7
1	0	
0	0	
0	0	
0	0	
1	0	
0	0	
0	0	
0	0	
0	0	
0	1	6
0	0	
0	0	
0	0	
0	0	
0	0	
0	1	6
0	1	9
0	1	7
1	0	
0	0	
0	1	7
0	0	
0	0	
0	0	
0	0	
0	0	
1	0	
0	0	
0	0	
0	0	
0	0	
0	0	
0	0	
0	0	
1	1	7
0	0	
0	0	
0	0	
0	0	
0	1	7
1	1	7

1	0	
0	0	
0	0	
0	0	
0	0	
0	0	
0	0	
0	0	
0	0	
1	0	
0	1	4
0	0	
0	0	
1	1	7
1	0	
0	0	
0	1	4
1	1	5
0	1	6
0	0	
0	0	
0	1	6
0	1	7
0	1	6
1	0	
0	0	
0	0	
0	0	
0	0	
1	1	8
0	0	
0	1	7
0	0	
0	0	
0	0	
0	0	
0	0	
0	0	
0	1	4
0	0	
0	0	
0	0	
0	0	
2	0	
0	1	6
0	0	
0	0	
0	0	
0	0	
0	0	
1	0	
0	0	
0	0	
0	0	
3	0	
0	1	5
0	1	5
0	0	
0	1	5
0	0	
0	1	6
1	0	
1	1	7
0	1	7
0	0	
0	0	
0	0	
1	0	
0	1	5
1	1	8
0	1	7
0	0	
2	0	
0	0	
0	0	
0	1	7
0	0	

0	1	4
1	1	8
0	0	
0	0	
0	0	
0	1	4
0	0	
0	1	6
0	0	
0	0	
2	0	
0	0	
0	1	7
0	0	
2	0	
0	0	
0	0	
0	0	
2	0	
0	0	
0	0	
0	0	
0	1	5
1	0	
0	1	7
0	0	
0	1	5
1	0	
0	1	9
0	1	7
0	0	
0	0	
1	1	7
0	0	
0	0	
0	0	
0	0	
0	0	
0	0	
0	0	
0	0	
0	0	
0	0	
0	0	
0	1	7
0	0	
0	0	
0	0	
0	1	5
0	0	
0	0	
0	1	9
0	0	
0	0	
0	0	
0	0	
0	0	
0	0	
0	0	
0	0	
2	0	
0	0	
2	0	
0	0	
0	0	
0	0	
4	0	
1	0	
0	0	
0	0	
0	0	
0	0	
0	1	5
0	1	9
0	1	9
0	0	
0	1	9

0	0	
0	0	
0	1	9
0	1	7
0	0	
0	0	
0	1	7
0	0	
0	0	
0	0	
0	0	
0	0	
0	1	4
0	0	
0	1	6
0	0	
0	0	
0	0	
0	1	7
0	1	7
0	0	
1	0	
0	0	
0	1	5
0	1	7
0	1	7
0	0	
0	1	5
0	1	4
0	0	
1	0	
0	1	9
0	1	7
0	1	7

ChronischeGastrointestinaleBeschwerden	ChronischeKopfschmerzen	ErkrankungdesBewegungsapparates
0	0	0
0	0	0
0	0	0
0	0	0
1	1	0
0	0	0
0	0	0
0	0	0
0	1	0
0	0	0
0	0	0
0	0	0
0	0	0
0	0	0
0	0	0
0	0	1
0	0	0
0	0	0
1	0	0
0	0	0
0	0	0
1	1	0
0	0	0
0	0	0
1	1	1
0	0	0
1	0	0
0	0	0
0	0	0
0	0	1
1	0	0
1	0	0
1	0	0
0	0	0
1	0	1
0	0	0
0	0	0
1	0	0
0	0	0
0	0	0
0	1	0
0	0	0
0	0	0
1	0	0
1	0	1
0	0	0
1	1	1
0	0	0
0	0	0
1	0	0
0	0	0
1	0	0
0	0	0
0	0	0
0	0	0
0	0	0
0	1	0
0	0	0
0	0	0
0	0	0
0	0	0
0	0	0
0	0	0
0	1	0
1	0	1
0	0	0
0	0	0
0	0	0
0	0	1
0	0	0
0	1	0
0	0	0
0	0	1
0	0	1
1	0	0
0	0	0

0	0	0
0	0	0
0	0	0
0	0	0
0	0	0
0	0	0
0	0	0
0	0	0
0	0	0
0	1	0
0	0	0
1	0	1
0	0	0
0	0	0
0	0	0
1	0	0
0	0	0
0	0	0
0	0	0
0	0	0
0	0	0
0	0	0
0	0	0
0	0	1
1	0	0
0	0	0
0	1	0
0	0	1
1	0	1
0	0	0
1	0	0
0	0	0
1	0	0
0	0	0
0	1	0
0	0	0
0	0	0
0	0	0
0	0	1
0	0	0
0	0	0
0	0	0
0	0	0
0	0	0
0	1	1
0	0	0
0	1	0
0	1	0
0	0	0
1	1	0
0	0	0
0	0	0
0	0	0
0	0	0
0	0	0
0	0	0
1	0	0
0	0	1
0	0	1
0	0	0
0	0	0
0	0	0
0	0	0
0	0	0
0	0	0
1	0	0
0	0	0
0	0	0
0	0	0
0	0	0
0	0	0
0	0	0
0	0	0
1	0	0
0	0	1
0	0	0
0	0	0

0	0	0
1	0	0
0	0	0
0	0	0
1	0	0
1	0	1
0	1	0
0	0	0
0	0	0
0	0	1
0	0	0
0	0	0
0	0	0
1	0	1
0	1	0
0	0	0
1	0	0
0	0	0
0	0	1
0	0	0
0	0	0
0	0	0
0	0	1
1	0	0
0	1	1
0	0	0
0	0	0
0	0	0
0	0	1
0	0	0
0	1	1
0	0	0
0	0	0
0	0	1
0	1	0
0	1	1
0	0	0
0	0	0
0	0	0
0	0	1
0	0	0
0	0	0
0	0	1
0	0	0
0	0	0
1	0	0
0	0	0
0	0	0
1	0	0
0	0	0
0	0	0
1	0	0
0	0	0
0	0	0
0	0	0
0	0	0
0	0	0
0	0	0
0	0	0
0	0	0
0	0	1
0	0	0
0	0	0
0	0	0
0	0	0
0	0	0
0	0	0
0	0	1
1	0	0
0	0	0
0		0
0	0	0
0	1	0
0	0	0
0	0	0
0	0	0
0	0	0

0	1	0
0	0	0
0	0	0
0	0	0
0	0	0
0	0	0
0	0	0
0	0	0
1	0	1
0	0	0
0	0	0
0	0	0
0	0	0
0	1	0
0	0	0
0	0	0
1	0	1
0	0	1
0	0	0
0	0	0
0	0	0
1	0	0
1	0	0
0	0	0
1	0	0
0	0	0
0	0	0
0	0	0
0	0	0
0	0	0
0	0	0
0	0	1
0	0	1
1	0	0
1	0	0
0	0	0
0	0	0
0	0	0
0	0	0
0	0	0
0	0	0
0	0	0
0	0	0
0	0	0
0	0	0
0	0	0
0	0	0
0	0	0
1	0	0
0	0	0
0	0	0
0	0	0
0	0	0
1	0	1
0	0	0
0	0	0
0	0	0
0	0	0
0	0	0
0	0	0
1	0	0
1	0	0
0	0	1
0	0	0
0	0	0
0	1	0
0	0	0
0	1	0
0	0	0
0	0	0
0	0	0
0	0	0
0	0	1
0	0	0
0	0	0
1	0	0
1	1	1

0	0	0
0	0	0
0	0	0
0	0	0
0	0	0
0	1	0
0	1	0
0	0	0
0	0	1
0	1	0
0	1	0
0	0	0
0	0	0
0	0	0
0	0	0
0	0	1
0	0	0
0	0	0
0	0	0
0	0	0
0	0	0
0	0	0
0	0	0
0	0	0
0	0	0
0	1	0
0	0	0
0	0	0
0	0	0
1	1	0
0	0	0
1	0	0
0	0	0
0	0	0
0	0	0
0	1	0
0	0	0
0	1	0
0	1	1
0	0	0
0	0	0
0	0	0
1	1	0
0	0	0
0	0	0
0	0	1
0	0	0
0	0	0
0	0	0
0	1	0
0	0	0
0	0	0
0	0	0
0	0	0
0	0	0
0	0	0
0	0	0
0	0	0
0	0	0
0	0	0
0	0	0
0	0	0
0	0	0
0	1	0
1	0	0
0	0	0
0	0	0
0	0	0
0	0	0
0	0	0
0	0	0
1	0	0
0	0	0
0	0	0
1	0	0
0	0	0
0	0	0

0	0	0
0	0	0
0	0	0
0	0	0
0	0	0
0	0	0
0	0	0
1	0	0
0	0	0
0	0	0
0	0	0
0	0	0
0	0	1
0	0	0
0	1	0
0	0	0
0	0	1
0	0	0
0	0	0
0	0	0
0	0	1
0	0	1
0	1	0
0	0	0
1	1	0
1	0	0
0	0	0
0	0	1
0	0	0
0	0	0
0	0	0
1	0	0
0	0	0
0	0	0
0	0	0
0	0	0
1	0	0
0	0	0
0	0	0
0	0	0
0	0	0
1	0	0
0	0	0
0	0	0
0	0	0
0	0	1
0	0	0
0	0	0
0	0	0
0	0	0
0	0	0
0	0	0
0	0	0
0	0	1
0	0	0
0	0	1
0	0	0
0	0	0
0	1	0
0	0	0
0	1	1
0	0	0
1	0	0
0	0	0
1	0	0
0	0	0
0	0	0
0	0	0
0	0	0
0	0	0
0	0	0
0	0	1
1	0	0
0	0	0
0	0	1
1	0	0
0	0	0

0	0	0
1	0	0
1	0	0
0	0	0
0	1	0
0	0	0
0	0	0
1	0	0
0	0	0
0	0	0
0	0	0
0	0	0
0	0	0
0	0	1
0	0	0
0	0	0
0	0	0
0	0	0
0	1	0
1	0	0
1	0	0
0	0	0
0	0	0
0	0	0
0	0	0
0	1	1
1	0	0
0	0	0
0	0	0
0	0	0
0	0	0
0	0	0
0	0	0
0	1	0
1	1	1
0	0	0
1	0	0
0	0	0
0	0	0
0	0	0
0	0	0
0	0	0
0	1	0
0	0	0
0	0	0
0	0	0
1	0	0
0	0	0
0	1	0
0	0	0
0	0	1
1	0	0
0	0	0
0	0	0
0	0	0
0	0	0
0	0	0
1	1	1
0	0	0
0	0	0
0	0	0
1	0	0
0	1	0
0	0	0
0	0	0
0	0	0
0	0	0
0	0	0
0	0	0
0	0	1
0	0	0
0	0	0
0	0	0
1	0	0
0	0	0
0	0	0
0	0	0

0	0	1
0	0	1
0	1	0
0	0	0
1	0	0
0	1	0
0	0	0
1	0	0
0	0	0
0	0	0
0	0	0
0	1	0
0	0	1
0	0	0
0	0	0
0	0	0
0	0	0
0	0	0
0	0	0
0	0	0
0	0	0
0	0	0
0	0	0
0	0	0
1	0	0
0	0	0
1	0	1
0	0	0
0	0	0
0	0	0
0	0	0
0	1	0
0	0	0
0	0	0
1	0	0
0	0	0
0	0	0
0	0	0
0	1	0
0	0	1
0	0	0
0	0	0
1	0	0
0	0	0
1	0	0
0	0	0
0	0	0
0	0	0
0	0	0
0	0	0
0	0	0
0	0	0
0	0	0
0	0	0
0	0	1
1	0	0
0	0	0
0	0	0
0	0	0
0	0	0
0	0	0
0	0	0
0	0	0
1	0	0
0	0	0
1	0	0
0	0	0
0	0	0
0	0	0
0	0	1
0	0	0
1	0	0
1	0	0
0	0	0
0	0	0
0	0	0
1	0	0

1	0	0
0	0	0
0	0	0
0	0	0
0	0	1
0	0	0
0	0	0
0	0	0
0	0	0
1	0	1
0	0	0
0	0	0
0	0	0
0	0	0
0	0	0
0	0	0
0	0	0
0	0	0
1	1	1
0	0	0
0	0	0
0	0	0
1	0	0
0	0	0
0	0	0
0	0	0
0	0	0
0	0	0
0	0	0
0	0	0
0	0	0
0	0	0
0	1	1
0	0	0
1	0	0
0	0	0
0	0	0
0	0	0
0	0	0
0	0	0
1	0	0
1	0	1
0	0	0
0	0	0
0	0	0
0	0	0
1	0	0
0	0	1
1	0	0
0		0
0	0	0
1	0	0
1	1	1
0	0	0
0	0	1
0	0	1
0	0	0
0	0	0
0	1	0
0	0	1
0	0	1
0	0	0
0	0	0
0	0	0
0	0	0
0	0	0
0	0	0
0	0	1
0	0	0
0	0	0
0	0	0
0	0	0
0	0	0
0	0	0
1	0	0
0	0	0
0	0	0

1	1	0
0	0	0
0	0	0
0	0	0
0	0	0
1	0	0
0	0	0
0	0	1
0	0	0
0	0	1
0	0	0
0	0	0
0	0	0
1	0	0
1	0	1
0	0	0
0	0	0
0	0	1
1	0	1
0	0	0
0	0	0
0	0	1
0	0	0
0	0	0
0	0	0
0	0	0
0	1	0
0	0	0
0	0	0
0	0	0
0	0	0
0	0	0
0	0	0
0	0	0
0	0	0
0	0	0
0	0	0
1	0	0
0	0	0
0	0	1
0	0	0
0	0	0
0	0	0
0	0	0
1	1	0
0	0	0
0	0	0
0	0	1
0	0	0
0	1	0
0	0	0
0	0	0
0	0	0
0	0	0
0	0	0
0	0	0
0	0	0
0	0	0
0	0	0
0	1	0
0	0	0
1	0	0
0	0	1
0	0	0
0	0	0
0	0	0
0	0	0
0	0	0
0	0	0
1	0	0
1	0	1
0	0	0
1	0	0
0	1	0
0	0	1
0	1	0
1	0	0

0	0	0
1	0	0
0	1	0
0	0	0
0	0	0
1	0	0
0	0	0
0	0	0
0	0	1
0	0	0
0	0	1
0	0	0
0	0	1
0	0	0
0	1	0
0	0	0
0	0	0
0	0	0
0	0	0
0	0	0
0	0	0
0	0	0
0	0	0
0	0	0
0	0	0
0	0	0
1	0	0
0	0	0
1	1	1
0	0	1
0	0	0
0	0	0
0	0	0
0	0	0
0	0	0
0	0	0
0	0	0
1	0	0
0	0	0
0	0	0
0	0	0
0	0	0
1	0	0
0	0	1
0	0	0
0	0	0
0	0	0
1	0	0
0	0	0
0	0	1
0	0	0
0	0	1
0	0	0
0	0	0
0	0	0
0	0	0
1	0	0
0	1	0
0	0	0
0	0	0
0	0	0
1	0	1
0	0	1
0	0	0
0	0	0
0	1	0
0	0	0
0	0	0
0	0	0
0	0	0
0	0	0
0	0	1
0	0	0
0	0	0
0	0	0
0	0	0
1	1	1

1	0	0
0	0	0
0	0	0
1	0	0
0	0	0
0	0	0
1	0	0
0	0	0
0	0	1
0	0	0
1	0	0
0	1	0
0	0	0
0	0	0
0	0	0
0	0	0
0	0	0
0	0	0
0	0	0
0	0	0
0	0	0
0	0	1
0	0	0
0	0	0
0	0	0
1	0	0
0	0	0
1	0	0
0	0	0
0	0	0
0	0	0
0	1	1
0	0	0
0	0	0

Appetitverlust	Schlafprobleme	Schwindel	Sucht	Sucht_Typ	Uebertragbare_KH	Infekt_KH
0	0	0	0		0	
0	0	0	0		0	
0	0	0	0		0	
0	0	0	0		0	
0	0	0	0		0	
0	0	0	0		0	
0	0	0	0		0	
1	0	0	0		0	
0	0	0	0		0	
0	0	0	0		0	
0	0	0	0		0	
0	0	0	0		0	
0	0	0	0		0	
0	0	0	0		0	
0	0	0	0		0	
0	0	0	1	4	0	
0	0	0	0		0	
0	0	0	0		0	
0	0	0	0		0	
0	0	0	0		0	
0	1	0	0		0	
0	0	0	0		0	
0	0	0	0		0	
0	1	0	0		0	
0	0	0	1	5	0	
0	0	0	0		0	
0	0	0	0		0	
0	0	0	0		0	
0	0	0	0		0	
0	0	0	0		0	
0	0	0	0		0	
0	0	0	0		0	
0	0	0	0		1	9
0	0	0	1	4	0	
1	0	0	0		1	13
0	0	0	0		0	
0	0	0	0		0	
0	0	0	0		0	
0	0	0	0		0	
0	0	0	0		0	
0	1	0	0		0	
0	0	0	0		0	
0	0	0	0		0	
0	0	0	0		0	
0	0	0	0		0	
0	0	0	0		0	
0	0	1	0		0	
0	0	0	0		0	
0	0	0	0		0	
0	0	0	0		0	
0	0	0	0		0	
0	0	0	0		0	
0	0	0	0		0	
0	0	0	0		0	
0	0	0	0		0	
0	0	0	0		1	13
0	0	0	0		0	
0	0	0	0		0	
0	0	0	0		0	
0	0	0	0		0	
0	0	0	0		0	
0	0	0	0		0	
0	0	0	1	4	0	
0	0	1	0		0	
0	0	0	0		0	
0	0	0	0		0	
0	0	0	1	4	0	
0	0	0	0		0	
0	0	0	0		0	
0	0	0	1	4	0	
0	1	0	0		0	
0	0	0	0		0	
0	0	0	0		0	
0	0	0	0		0	
0	0	0	0		1	6
0	0	0	1	5	0	

1	0	0	0		0
0	0	0	0		0
0	0	0	0		0
0	0	0	0		0
0	0	0	0		0
0	0	0	0		0
0	0	0	0		0
0	0	0	0		0
0	0	0	0		0
0	0	1	0		0
0	0	0	0		0
0	0	0	0		0
0	0	0	0		0
0	0	0	0		0
0	0	0	0		0
0	0	0	0		0
0	0	0	0		0
0	0	0	0		0
0	0	0	0		0
0	0	0	0		0
0	0	0	0		0
0	0	0	0		0
0	0	0	0		0
0	0	0	0		0
0	0	0	0		0
0	0	0	0		0
0	0	0	0		0
0	0	0	0		0
0	0	0	0		0
0	0	0	0		0
0	0	0	0		0
0	0	0	0		0
0	0	0	0		0
0	0	0	0		0
0	0	0	1	5	0
0	0	0	0		0
0	0	0	0		0
0	0	0	0		0
0	0	0	0		0
0	0	0	0		0
0	0	0	0		0
0	0	0	0		0
0	0	0	0		0
0	0	1	0		
1	1	1	0		0
0	0	0	0		0
0	0	1	0		0
0	0	0	1	5	0
0	0	0	0		0
0	0	1	0		0
	0	0	0		0
0	0	0	0		0
0	0	0	0		0
0	0	0	0		0
0	0	0	0		0
0	0	0	0		0
0	0	0	0		0
0	0	0	0		0
1	0	1	0		0
0	0	0	0		0
0	0	0	0		0
0	0	0	1	4	0
0	0	0	0		0
0	0	0	1	4	0
0	0	0	0		0
0	0	0	0		0
0	0	0	0		0
0	0	0	0		0
0	0	0	0		0
0	0	0	0		0
0	0	0	0		0
0	0	0	0		0
0	0	0	0		0
1	0	0	0		0
0	0	0	0		0
0	0	0	0		0
0	0	0	0		0

0	1	0	0		0
0	0	0	0		0
0	0	0	0		0
0	0	0	0		0
0	0	0	0		0
0	0	0	1	4	0
0	0	0	1	5	0
0	0	0	0		0
0	0	0	1	5	0
0	0	0	0		0
0	0	0	0		0
0	0	0	0		0
0	0	0	0		0
0	0	0	0		0
0	0	0	0		0
0	0	0	0		0
0	0	0	0		0
0	0	0	0		0
0	0	0	0		0
0	0	0	0		0
0	0	0	0		0
0	0	0	0		0
0	0	0	0		0
1	0	0	0		0
0	0	1	1	5	0
0	0	0	0		0
0	0	0	0		0
0	0	0	0		0
0	0				
1	0	0	0		0
0	0	0	0		0
0	0	0	0		0
0	0	0	0		0
0	0	0	0		0
0	0	0	0		0
0	0	0	0		0
0	0	0	0		0
0	0	0	0		0
1	0	0	0		0
0	0	0	0		0
0	0	0	0		0
0	0	0	0		0
0	0	0	0		0
0	0	0	0		0
0	0	0	0		0
0	0	0	0		0
0	0	1	0		0
0	0	0	0		0
0	0	0	0		0
0	0	0	0		0
0	0	0	0		0
0	0	0	0		0
0	1	0	1	5	0
0	0	0	0		0
0	0	0	0		0
0	0	0	0		0
0	0	0	0		0
0	0	0	0		0
0	0	0	0		0
0	0	0	0		0
0	0	0	0		0
0	0	0	0		0
0	0	0	0		0
0	0	0	0		0
0	0	0	1	5	0
0	0	0	0		0
0	0	0	0		0
0	0	0	0		0
0	0	1	0		0
0	0	0	0		0
0	0		1	4	0
1	0	0	0		0
0	0	1	0		0
0	0	0	0		0
0	0	0	0		0
0	0	0	0		0
0	0	0	0		0

0	0	0	0		0	
0	0	0	0		0	
0	0	0	0		0	
0	0	0	0		0	
0	0	0	0		0	
0	0	0	0		0	
0	0	0	0		0	
0	0	0	0		0	
0	0	0	0		0	
0	0	0	0		0	
0	0	0	0		0	
0	0	0	0		0	
0	0	0	0		0	
0	0	0	0		0	
0	0	0	0		0	
0	0	0	1	5	0	
0	0	0	0		0	
0	0	0	0		0	
0	0	0	0		0	
0	0	0	0		0	
0	0	0	0		0	
0	0	0	0		0	
0	0	0	0		0	
0	0	0	0		0	
0	0	0	0		0	
0	0	0	0		0	
0	0	0	0		0	
0	0	0	0		0	
0	0	0	0		0	
0	0	0	0		0	
1	0	0	0		0	
0	0	0	0		0	
0	0	0	0		0	
1	0	0	0		0	
0	0	0	0		0	
0	0	0	1	4	0	
0	0	0	0		0	
0	0	0	0		0	
0	0	0	0		0	
0	0	0	0		0	
0	0	0	0		0	
0	0	0	0		0	
0	0	0	0		0	
0	0	0	0		0	6
0	0	0	0		0	
0	0	0	0		0	
0	0	0	0		0	
0	0	0	0		0	
0	0	0	0		0	
0	0	0	0		0	
0	0	0	0		0	
0	0	0	0		0	
0	0	0	0		0	
0	0	0	0		1	5
0	0	0	0		0	
1	0	0	0		0	
0	0	0	1	5	0	
0	0	0	0		0	
0	0	0	0		0	
0	0	0	0		0	
0	0	0	0		0	
0	0	0	0		0	
0	0	0	0		0	
0	0	0	0		0	
0	0	0	0		0	
0	0	0	0		0	
0	0	0	0		0	
0	0	0	0		0	
0	0	0	0		0	
0	1	0	0		0	
0	0	0	0		0	
0	0	0	0		0	
0	0	0	0		0	
0	0	0	0		0	
0	0	0	0		0	
0	0	0	0		0	
0	1	0	0		0	

0	0	0	0		0	
0	0	0	0		0	
0	0	0	0		0	
0	0	0	0		0	
0	0	0	0		0	
0	0	0	0		0	
0	0	0	0		0	
0	0	0	1	4	0	
0	0	0	0		0	
0	0	0	0		0	
0	0	0	0		0	
0	0	0	0		0	
0	0	0	0		0	
0	0	0	0		0	
0	0	0	0		0	
0	1	0	0		0	
0	0	0	0		0	
0	0	0	0		0	
0	0	0	0		0	
0	0	0	0		0	
0	0	0	0		0	
0	0	0	0		0	
0	0	0	0		0	
0	0	0	0		0	
0	1	0	0		0	
0	0	0	0		0	
0	0	0	0		0	
0	0	0	0		0	
0	0	0	0		0	
0	0	1	0		0	
	0	0	0		0	
0	0	0	0		0	
0	0	0	0		0	
0	0	0	1	4	0	
0	0	0	0		0	
0	0	0	0		0	
0	0	0	0		0	
0	0	0	0		0	
0	0	0	0		0	
0	0	0	0		0	
0	0	0	0		0	
0	0	0	0		0	
1	1	1	0		0	
0	0	0	0		0	
0	0	0	0		0	
0	0	0	0		0	
0	0	0	0		0	
0	0	1	0		0	
0	0	0	0		1	12
0	0	0	0		0	
0	0	0	0		0	
0	0	0	0		0	
0	0	0	0		0	
0	0	0	0		0	
0	0	0	0		0	
0	0	0	0		0	
0	0	0	0		0	
0	0	0	0		0	
0	0	0	0		0	
0	0	0	0		0	
0	0	0	0		0	
0	0	0	0		0	
0	0	0	0		0	
0	0	0	0		0	
0	0	0	0		0	
0	0	0	0		0	
0	0	0	0		0	
0	0	0	0		0	
0	0	0	0		0	
0	0	0	0		0	
0	0	0	0		0	
1	0	0	0		0	
1	1	0	1	4	0	
0	0	0	0		0	
0	0	0	1	4	0	
0	0	0	0		0	
0	0	0	0		0	

0	0	0	0		0	
0	0	0	0		0	
0	0	0	0		0	
0	0	0	0		0	
0	0	0	0		0	
0	0	0	0		0	
0	0	0	0		0	
0	0	0	0		0	
0	0	0	0		0	
0	0	0	0		0	
0	0	1	0		0	
0	0	0	0		0	
0	0	0	0		0	
0	0	0	0		0	
0	0	0	0		0	
0	0	0	0		0	
0	0	0	0		0	
0	0	0	0		0	
0	0	0	0		0	
0	0	0	1	5	0	
0	0	0	0		0	
0	0	0	0		1	7
0	0	0	0		0	
0	0	0	1	4	0	
0	0	0	0		0	
0	0	0	0		0	
0	0	0	0		0	
0	0	0	0		0	
0	0	0	0		0	
0	0	0	0		0	
0	0	0	0		0	
0	0	0	1	4	0	
0	0	0	0		0	
0	0	0	0		0	
0	0	0	0		0	
0	0	0	0		0	
0	0	0	0		0	
0	0	0	0		0	
0	0	0	0		0	
0	0	0	0		0	
0	0	0	0		0	
0	0	0	0		0	
0	0	0	0		0	
0	0	0	1	4	0	
0	0	0	1	4	0	
0	0	0	0		0	
0	0	0	0		0	
0	0	0	0		0	
0	0	0	0		1	10
0	0	1	0		0	
0	0	0	0		1	8
0	0	0	0		0	
0	0	0	0		0	
0	0	0	1	5	0	
0	0	0	0		0	
0	0	1	0		0	
0	0	0	0		1	4
0	0	0	1	4	0	
1	0	0	0		0	
0	0	0	0		0	
0	0	0	0		0	
0	0	0	0		0	
0	0	0	0		0	
0	0	0	0		1	4
0	0	0	0		0	
0	0	0	0		0	
0	0	0	0		0	
0	0	0	0		0	
0	0	0	0		0	
0	0	0	0		0	
0	0	0	0		0	
0	0	0	0		0	
0	0	0	1	5	0	
0	0	0	0		0	
0	0	0	0		0	
0	0	0	0		1	6
0	0	0	0		0	

0	0	0	0		0	
0	0	0	0		1	13
1	0	1	0		0	
0	0	0	0		0	
0	0	1	0		0	
0	0	0	0		0	
0	0	0	0		0	
0	0	0	0		0	
0	0	0	0		0	
0	0	0	0		0	
0	0	0	0		0	
0	0	0	0		0	
0	0	0	0		0	
0	0	0	0		1	13
0	0	0	0		0	
0	0	0	0		0	
0	0	0	0		0	
0	0	0	0		0	
0	0	0	0		0	
0	0	0	0		0	
0	0	0	0		0	
0	0	0	0		0	
0	0	0	0		0	
0	0	0	0		0	
0	0	0	1	4	0	
0	1	1	0		0	
0	0	0	0		0	
0	0	0	0		0	
0	0	0	0		0	
0	0	0	0		0	
0	0	0	0		0	
0	0	0	0		0	
0	0	0	0		0	
0	0	1	0		0	
0	0	0	0		0	
0	0	0	0		0	
0	0	0	0		0	
0	0	0	0		0	
0	0	0	0		0	
0	0	0	0		0	
0	0	0	0		0	
0	0	0	0		0	
0	0	0	0		0	
0	0	0	0		0	
0	0	0	0		0	
0	0	0	0		0	
0	0	0	0		0	
0	0	0	0		1	13
0	1	0	0		0	
0	0	0	0		0	
0	0	0	0		0	
1	1	0	0		0	
0	0	0	0		0	
0	0	0	0		0	
0	0	0	0		0	
0	0	0	0		0	
0	0	0	0		0	
0	0	0	1	5	0	
0	0	0	0		0	
0	0	0	0		0	
0	0	0	0		0	
0	0	0	0		0	
0	0	0	0		0	
0	0	0	1	4	0	
0	0	0	0		0	
0	0	0	0		0	
0	0	0	0		0	
0	0	0	0		0	
0	0	0	0		0	
0	0	0	0		0	
0	0	0	0		0	
0	0	0	0		0	
0	0	0	0		0	
0	0	0	0		0	
0	0	0	0		0	
0	0	0	0		0	
0	0	0	0		0	

0	0	0	0		1	13
0	0	0	0		0	
0	0	0	0		0	
0	0	0	0		0	
0	0	0	0		0	
0	0	0	0		0	
0	0	0	0		0	
0	0	0	0		0	
0	0	0	0		0	
1	0	0	0		0	
0	0	0	0		0	
0	0	0	0		0	
0	0	0	0		0	
0	0	0	0		0	
0	0	0	1	4	0	
0	0	0	0		0	
0	0	0	0		0	
0	0	0	0		0	
0	0	0	1	5	0	
0	0	0	0		0	
0	0	0	0		0	
0	0	0	0		0	
0	0	0	0		0	
0	0	0	0		0	
0	0	0	0		0	
0	0	0	0		0	
0	0	0	0		0	
0	0	0	0		0	
0	0	0	0		0	
0	0	0	0		0	
0	0	0	0		0	
0	0	0	0		0	
0	0	0	0		0	
0	1	0	0		0	
0	0	0	0		0	
0	0	0	0		0	
0	0	0	0		0	
0	0	0	0		0	
0	0	0	0		0	
0	0	0	0		0	
0	0	0	0		0	
0	0	0	0		0	
0	0	0	0		0	
0	0	1	0		0	
0	0	0	0		0	
0	0	0	0		0	
0	0	0	0		0	
0	0	0	0		0	
0	0	0	0		0	
0	0	0	0		1	13
0	0	0	0		0	
0	0	0	0		0	
0	0	0	0		0	
0	0	0	0		0	
0	0	0	0		0	
0	0	0	0		0	
0	0	0	0		0	
0	0	0	0		0	
0	0	0	0		0	
0	0	0	0		0	
0	0	0	0		1	11
0	0	0	0		0	
0	0	0	0		0	
0	0	0	0		0	
0	0	0	0		0	
0	0	0	0		0	
0	0	0	0		0	
0	0	0	0		0	
0	0	0	0		0	
0	0	0	0		0	
0	0	0	0		0	
0	0	0	0		0	
0	0	0	0		0	
0	0	0	0		0	
0	1	0	0		0	
0	0	0	0		0	
0	0	0	0		0	

0	0	0	0		0
0	0	0	0		0
0	0	0	0		0
0	0	0	0		0
0	0	0	0		0
0	0	0	0		0
0	0	0	0		0
0	0	0	1	4	0
0	0	0	0		0
0	0	0	0		0
0	0	0	0		0
0	0	0	0		0
0	0	0	0		0
0	0	0	0		0
0	0	0	0		0
0	0	0	0		0
0	0	0	0		0
0	0	0	0		0
0	0	0	0		0
0	0	0	0		0
0	0	0	0		0
0	0	0	0		0
0	0	0	0		0
0	0	0	0		0
0	0	0	0		0
0	0	0	0		0
0	0	0	0		0
0	0	0	1	5	0
0	0	0	0		0
0	0	0	0		0
0	0	0	0		0
0	0	0	0		0
1	0	0	0		0
0	0	0	0		0
0	0	0	0		0
0	0	0	0		0
0	0	0	0		0
0	0	0	0		0
0	0	0	0		0
0	0	0	0		0
0	0	0	0		0
0	0	0	0		0
0	0	0	0		0
0	0	0	0		0
0	0	0	0		0
0	0	0	0		0
0	0	0	0		0
0	0	0	0		0
0	0	0	0		0
0	0	0	0		0
0	0	0	0		0
0	0	0	0		0
0	1	0	0		0
0	0	0	0		0
0	0	0	0		0
0	0	0	0		0
0	0	0	0		0
0	0	0	0		0
0	0	0	0		0
0	0	0	0		0
0	0	0	0		0
0	0	0	0		0
0	0	0	0		0
0	0	0	0		0
0	0	0	0		0
0	0	0	0		0
0	0	0	0		0
0	0	0	0		0
0	0	0	0		0
0	0	0	0		0
0	0	0	0		0
0	0	0	0		0
0	0	0	0		
0	0	0	0		0
0	0	1	0		0
0	0	0	0		0
0	0	0	0		0

0	1	0	0		0	
0	0	0	0		0	
0	0	0	0		0	
0	0	0	0		0	
0	0	0	0		0	
0	0	0	0		0	
0	0	0	0		0	
0	0	0			0	
0	0	0	0		0	
0	0	0	0		0	
0	0	0	1	5	0	
0	0	0	0		0	
0	0	0	0		0	
0	0	0	0		0	
0	0	0	0		1	13
0	1	0	0		0	
0	0	0	1	5	0	
0	0	0	0		0	
0	0	0	1	5	0	
0	0	0	0		0	
0	0	0	0		0	
0	0	0	0		1	13
0	0	0	0		0	
0	0	0	0		0	
0	0	0	0		0	
0	0	0	0		0	
0	0	0	0		0	
0	0	0	0		0	
0	0	0	0		0	
0	0	0	0		0	
0	0	0	0		0	
0	0	0	0		0	
0	0	0	0		0	
0	0	0	0		0	
0	0	0	0		0	
0	0	0	0		0	
0	0	0	0		1	12
0	0	0	0		0	
0	0	0	1	4	0	
0	0	0	0		0	
0	0	0	0		0	
0	0	0	0		0	
0	0	0	0		0	
0	0	0	0		0	
0	0	0	0		0	
0	0	0	0		0	
0	0	0	0		0	
0	0	0	0		0	
0	0	0	0		1	6
0	0	0	0		0	
0	0	0	0		0	
0	0	0	0		0	
0	0	0	0		0	
0	0	0	0		0	
0	0	0	0		0	
1	1	0	0		0	
1	1	0	0		0	
0	0	0	0		0	
0	1	0	1	4	0	
0	0	0	0		0	
0	0	0	1	4	0	
0	0	0	0		0	
0	1	0	0		0	
0	0	0	0		0	
0	0	0	0		0	
0	0	0	0		0	
0	0	0	0		0	
0	0	0	0		0	
0	0	0	0		0	
1	0	0	1	4	0	
0	0	0	0		0	
1	0	0	0		0	
0	0	0	0		0	
0	0	0	0		0	
0	0	0	0		0	
0	0	0	0		0	

0	0	0	1	5	0	
0	0	0	0		0	
0	0	0	0		0	
0	0	0	0		0	
0	0	0	0		0	12
0	0	0	1	4	0	
0	0	0	0		0	
0	0	0	0		0	
0	0	0	0		0	
0	0	0	0		0	
1	1	0	0		0	
0	0	0	0		0	
0	0	0	0		0	
0	0	0	0		0	
0	0	1	0		0	
0	0	0	0		0	
0	0	0	0		0	
0	0	0	0		0	
0	0	0	0		0	
0	0	0	0		0	
0	0	0	0		0	
0	0	0	0		0	
0	0	0	0		0	
0	0	0	0		0	
0	0	0	0		0	
0	0	0	0		0	
0	0	0	0		0	
0	0	0	0		0	
0	0	0	0		0	
0	0	1	0		0	
0	0	0	0		0	
0	0	0	0		0	
0	1	0	1	4	0	
0	0	0	0		0	
0	0	0	0		0	
0	0	0	0		0	
0	0	0	0		0	
0	0	0	0		0	
0	0	0	0		0	
0	0	0	0		0	
0	0	0	0		0	
0	0	0	0		0	
0	0	0	0		0	
0	1	0	0		0	
1	1	0	0		0	
0	0	0	0		0	
0	0	0	0		0	
0	0	0	0		0	
0	1	0	1	5	0	
0	0	0	0		0	
0	0	0	0		0	
0	0	0	0		0	
0	0	0	0		0	
0	0	0	0		0	
0	0	0	0		0	
0	0	0	0		0	
0	0	0	0		0	
0	0	0	0		0	
0	0	0	0		0	
0	0	0	0		0	
0	0	0	0		0	
0	0	0	0		0	
0	0	0	0		0	
0	0	0	0		0	
0	0	0	0		0	
0	0	0	0		0	
0	0	0	0		0	
0	0	0	0		0	
0	0	0	0		0	
0	0	0	0		0	
0	0	0	0		0	
0	0	0	0		0	
0	1	0	0		0	
0	0	0	0		0	
0	0	0	0		0	
0	0	0	0		0	
0	0	0	0		0	

0	0	0	0		0
0	0	0	0		0
0	0	0	0		0
0	0	0	0		0
0	0	0	0		0
0	0	0	0		0
0	0	0	0		0
0	0	0	0		0
0	0	0	0		0
0	0	0	0		0
0	0	0	0		0
0	0	0	0		0
0	0	0	1	4	0
0	0	0	0		0
0	0	0	0		0
0	0	0	0		0
0	0	0	0		0
0	0	0	0		0
0	0	0	0		0
0	0	0	0		0
0	0	0	0		0
0	0	0	0		0
0	0	0	0		0
0	0	0	0		0
0	0	0	0		0
0	0	0	1	5	0
0	0	0	0		0
0	0	0	1	4	0
0	0	0	1	4	0
0	0	0	0		0
0	0	0	0		0
0	0	0	0		0
0	0	0	0		0
0	0	0	0		0

Hospitalisation	VAR00004	VAR00003	VAR00002	VAR00001	IntensivstationIMC	InnereMedizin
0					0	1
0					0	0
0					0	1
0					0	1
0					0	0
0					0	0
0					0	0
0					0	0
0					0	0
0					0	0
0					0	0
0					0	1
0					0	0
0					0	0
1					0	?
1					0	1
0					0	0
1					0	0
0					0	1
0					0	1
1					0	0
1					0	1
0					0	0
1					0	0
0					0	1
0					0	0
0					0	1
0					0	1
0					0	1
0					0	0
1					0	1
0					0	1
0					0	1
0					0	0
0					0	?
0					0	1
0					0	0
1					0	1
0					0	0
0					0	1
0					0	0
0					0	0
0					0	1
0					0	0
0					0	1
0					0	1
0					0	1
0					0	1
0					0	1
0					0	1
1					0	0
0					0	1
0					0	0
0					0	0
0					0	1
1					0	1
0					0	0
0					0	?
0					0	0
0					0	1
0					0	1
0					0	0
0					0	1
0					0	1
0					0	1
0					0	0
0					0	0
0					0	0
0					0	1
0					0	1
0					0	0
0					0	0
1					0	0
1					0	0
0					0	1
0					0	0

0	0	1
0	0	1
0	0	1
0	0	1
0	0	0
0	0	0
0	0	1
0	0	1
0	0	0
0		1
0		0
0		1
0		0
0		0
0		1
0		0
0		1
0		0
0		0
0		1
0		0
0		0
0		0
0		0
0		0
0		0
0		1
0		1
0		1
0		0
0		1
0		1
0		1
0		0
0		0
0		0
0		0
0		0
0		0
0		1
0		0
0		0
0		0
0		0
0		1
0		0
0		0
0		0
0		1
0		1
0		0
0		0
0		0
0		0
0		1
0		?
0		1
0		1
0		0
1		0
0		0
0		?
0		1
0		1
0		0
0		1
0		0
0		0
0		0
0		0
0		1
0		0
0		0
0		1
0		0
0		0
0		0

0		0
0		0
0		0
0		0
0		1
0		?
0		0
0		0
0		0
0		1
0		0
0		0
0		0
0		0
0		0
0		0
0		1
0		0
0		0
0		0
0		0
0		0
0		0
0		1
0		1
0	0	0
0		0
0	0	1
0	0	1
0	0	0
0		0
0	0	0
0	0	1
0	0	0
0	0	1
0	0	0
0	0	0
0	0	0
0	0	1
0	0	0
0	0	0
0	0	0
0	0	0
0	0	0
0	0	0
0	0	1
0	0	0
0	0	1
0	0	1
0	0	1
0	0	0
0	0	1
0	0	0
0	0	0
0	0	0
0	0	1
0	0	0
0	0	0
0	0	0
0	0	?
0	0	0
0	0	0
0	0	0
0	0	0
0	0	0
0	0	0
0	0	1
0	0	1
0	0	0
0	0	0
0	0	1
0	0	0
	0	0
0	0	0
0	0	0
0	0	0
0	0	0

0	0	?
0	0	0
0	0	0
0	0	1
0	0	0
0	0	0
0	0	0
0	0	?
0	0	0
0	0	1
0	0	0
0	0	0
0	0	0
0	0	0
0	0	0
0	0	0
0	0	1
0	0	0
0	0	0
0	0	0
0	0	0
0	0	1
0	0	1
0	0	1
0	0	?
0	0	0
0	0	0
0	0	1
0	0	0
0	0	1
0	0	0
0	0	1
0	0	0
0	0	1
0	0	1
0	0	1
0	0	0
0	0	1
0	0	0
0	0	0
0	0	1
0	0	0
0	0	1
0	0	0
0	0	0
0	0	0
0	0	0
0	0	0
0	0	1
0	0	0
0	0	0
0	0	0
0	0	0
0	0	1
0	0	0
0	0	1
0	0	1
0	0	0
0	0	0
0	0	0
0	0	1
0	0	1
0	0	0
0	0	1
0	0	0
0	0	0
0	0	1
0	0	1
0	0	1
0	0	0
0	0	0
0	0	?
0	0	0
0	0	1
0	0	0
0	0	1
0	0	0

0	0	1
0	0	0
0	0	1
0	0	1
0	0	0
0	0	0
0	0	0
0	0	1
0	0	?
0	0	0
0	0	0
0	0	0
0	0	0
0	0	0
0	0	0
0	0	0
0	0	0
0	0	1
0	0	1
0	0	0
0	0	0
0	0	1
0	0	0
0	0	1
0	0	0
0	0	0
0	0	0
0	0	1
0	0	0
0	0	1
0	0	0
0	0	1
0	0	1
0	0	1
0	0	1
0	0	0
0	0	0
0	0	0
0	0	1
0	0	1
0	0	0
0	0	0
0	0	1
0	0	0
0	0	0
0	0	1
0	0	0
0	0	0
0	0	1
0	0	0
0	0	0
0	0	0
0	0	1
0	0	0
0	0	0
0	0	1
0	0	0
0	0	0
0	0	1
0	0	0
0	0	0
0	0	0
0	0	1
0	0	0
0	0	1
0	0	0
0	0	0
0	0	0
0	0	0
0	0	0
0	0	1
0	0	1
0	0	0
0	0	0
0	0	?
0	0	0
0	0	1

0	0	0
0	0	0
0	0	1
0	0	0
0	0	0
0	0	0
0	0	0
0	0	?
0	0	1
0	0	0
0	0	0
0	0	1
0	0	0
0	0	0
0	0	0
0	0	1
0	0	0
0	0	1
0	0	0
0	0	0
0	0	1
0	0	1
0	0	?
0	0	1
0	0	1
0	0	1
0	0	0
0	0	0
0	0	0
0	0	0
1	0	?
0	0	1
0	0	0
0	0	0
0	0	0
0	0	0
1	0	1
1	0	1
0	0	0
0	0	?
0	0	0
0	0	1
1	0	0
1	0	0
1	0	0
0	0	0
0	0	1
0	0	0
0	0	0
0	0	1
0	1	1
0	0	0
0	0	0
0	0	1
1	0	1
0	0	0
0	0	1
1	0	0
0	0	1
0	0	0
0	0	0
0	0	1
0	0	1
0	0	1
0	0	1
0	0	1
0	0	0
0	0	1
0	0	1
0	0	0
0	0	0
0	0	0
1	0	1
0	0	1
0	0	0
1	0	1
0	0	1

0	0	0
0	0	1
0	0	1
0	0	1
0	0	0
0	0	0
0	0	1
0	0	1
0	0	?
1	0	1
0	0	1
0	0	0
0	0	1
0	0	0
0	0	0
0	0	0
0	0	0
1	0	0
0	0	1
0	0	1
0	0	1
0	0	0
0	0	0
0	0	0
0	0	1
0	0	0
0	0	0
0	0	1
0	0	?
0	0	0
0	0	0
0	0	0
0	0	0
0	0	0
0	0	1
0	0	1
0	0	1
0	0	0
0	0	1
0	0	1
0	0	0
1	0	0
0	0	0
1	0	0
0	0	1
0	0	0
1	0	1
0	0	1
1	0	0
0	0	0
0	0	0
0	0	1
0	0	?
0	0	1
0	0	0
0	0	1
0	0	1
0	0	0
0	0	0
0	0	1
0	0	0
0	0	0
0	0	1
1	0	1
0	0	1
1	0	0
0	0	?
0	0	1
0	0	0
0	0	0
0	0	1
0	0	0
0	0	0
0	0	0
0	0	0
0	0	0
0	0	1

0	0	0
0	0	1
0	0	0
0	0	0
0	0	0
0	0	0
0	0	0
0	0	1
1	0	1
0	0	1
0	0	1
0	0	0
0	0	1
0	0	1
0	0	0
0	0	0
0	0	1
0	0	0
1	0	1
0	0	1
0	0	1
0	0	1
0	0	0
0	0	1
0	0	1
0	0	0
0	0	0
0	0	0
0	0	1
0	0	1
0	0	0
0	0	0
0	0	1
0	0	0
0	0	0
0	0	1
1	0	0
0	0	0
0	0	0
0	0	1
0	0	0
0	0	0
0	0	1
0	0	0
0	0	1
0	0	1
0	0	0
0	0	0
0	0	1
0	0	1
0	0	1
0	0	0
0	0	0
0	0	1
0	0	0
1	0	0
0	0	1
0	0	0
0	0	0
0	0	0
0	0	?
0	0	0
0	0	1
0	0	1
0	0	0
0	0	?
0	0	0
0	0	0
0	0	1
0	0	1
1	0	0
0	0	0
0	0	0
0	0	0
0	0	1
0	0	1
0	0	1

0	0	1
0	0	0
0	0	1
1	0	0
0	0	1
0	0	0
0	0	1
1	0	0
0	0	0
0	0	0
0	0	0
0	0	0
0	0	0
0	0	0
0	0	1
0	0	0
0	0	1
0	0	0
0	0	1
0	0	0
0	0	0
0	0	0
0	0	0
0	0	0
0	0	1
0	0	1
1	0	0
0	0	1
0	0	1
0	0	0
0	0	0
0	0	1
0	0	0
0	0	1
1	0	1
1	0	1
0	0	1
0	0	1
0	0	1
0	0	0
0	0	1
0	0	0
0	0	1
0	0	0
0	0	0
0	0	?
0	0	1
0	0	0
0	0	0
0	0	1
0	0	1
0	0	0
0	0	0
0	0	0
0	0	0
0	0	1
0	0	?
0	0	1
0	0	0
0	0	0
0	0	0
0	0	0
0	0	0
0	0	0
0	0	0
0	0	0
0	0	0
0	0	1
0	0	1
0	0	0
1	0	0
0	0	0
0	0	0
0	0	0
1	0	0
0	0	0
0	0	1

1	0	0
0	0	0
0	0	1
0	0	0
0	0	0
0	0	1
0	0	1
0	0	1
0	0	0
0	0	1
0	0	0
0	0	0
0	0	0
0	0	1
0	0	1
0	0	0
0	0	0
0	0	1
0	0	1
0	0	0
0	0	0
0	0	0
0	0	0
0	0	0
0	0	0
0	0	0
0	0	1
0	0	1
0	0	1
1	0	1
0	0	1
1	0	0
0	0	0
0	0	1
0	0	1
1	0	1
0	0	?
0	0	1
0	0	0
0	0	0
0	0	0
1	0	0
0	0	0
0	0	0
0	0	1
0	0	1
0	0	0
0	0	1
0	0	1
0	0	0
0	0	?
0	0	1
0	0	0
1	0	0
0	0	1
1	0	0
0	0	0
0	0	1
0	0	0
0	0	0
1	0	0
0	0	?
1	0	0
1	0	0
0	0	0
0	0	0
0	0	0
0	0	0
0	0	0
1	0	1
0	0	1
0	0	1
0	0	1
0	0	0
0	0	0
1	0	0
0	0	1

0	0	0
0	0	1
0	0	0
0	0	1
0	0	0
0	0	1
0	0	0
1	0	?
0	0	0
0	0	0
1	0	0
0	0	1
0	0	0
0	0	0
1	0	0
0	0	0
0	0	1
1	0	0
0	0	1
0	0	0
0	0	0
0	0	0
1	0	?
0	0	1
0	0	1
0	0	1
1	0	0
0	0	1
0	0	0
0	0	0
0	0	0
0	0	0
1	0	0
0	0	0
0	0	0
0	0	1
0	0	1
0	0	1
0	0	0
0	0	0
0	0	1
0	0	0
0	0	0
0	0	0
1	0	0
0	0	0
0	0	1
0	0	1
0	0	1
0	0	0
0	0	0
0	0	?
0	0	0
0	0	1
0	0	0
0	0	1
0	0	1
0	0	0
0	0	0
0	0	1
0	1	0
0	0	1
1	0	1
1	0	1
1	0	1
0	0	0
1	0	0
0	0	1
0	0	0
0	0	1
0	0	0
0	0	?
1	0	0
0	0	0
0	0	0
0	0	0
0	0	0

0	0	0
0	0	0
0	0	0
0	0	1
0	0	1
1	0	?
0	0	1
0	0	0
0	0	0
0	0	0
0	0	0
0	0	0
0	0	1
0	0	0
1	0	?
0	0	0
1	0	0
0	0	1
0	0	0
0	0	0
1	0	?
0	0	0
0	0	1
1	0	0
0	0	0
0	0	1
0	0	0
0	0	1
1	0	0
0	0	0
0	0	?
0	0	0
0	0	1
0	0	1

ChirurgiekeinTrauma	VCH	HCH	SKG	NCH	Ortho	Urologie	Gynäkologie	Trauma	Sturz
0	0	0	0	0	0	0	0	0	0
0	0	0	0	0	0	0	0	1	1
0	0	0	0	0	0	0	0	0	0
0	0	0	0	0	0	0	0	0	0
0	0	0	0	0	0	0	0	0	0
0	0	0	0	0	0	0	0	1	1
0	0	0	0	0	0	0	0	0	0
0	0	0	0	0	0	0	0	0	0
0	0	0	0	0	0	0	0	1	1
1	0	0	0	0	0	0	0	0	0
0	0	0	0	0	0	0	0	0	0
0	0	0	0	0	0	0	0	0	0
1	0	0	0	0	0	0	0	0	0
0	0	0	0	0	0	0	0	1	1
0	0	0	0	0	0	0	0	0	0
0	0	0	0	0	0	0	0	0	0
0	0	0	0	0	0	0	0	0	0
0	0	0	0	0	0	1	0	0	0
0	0	0	0	0	0	0	0	0	0
0	0	0	0	0	0	0	0	0	0
0	0	0	0	0	0	0	0	0	0
0	0	0	0	0	0	0	0	0	0
0	0	0	0	0	0	0	0	1	0
0	0	0	0	0	0	0	0	0	0
0	0	0	0	0	0	0	0	0	0
0	0	0	0	0	0	1	0	0	0
0	0	0	0	0	0	0	0	0	0
0	0	0	0	0	0	0	0	0	0
0	0	0	0	0	0	0	0	0	0
0	0	0	0	0	0	0	0	1	1
0	0	0	0	0	0	0	0	0	0
0	0	0	0	0	0	0	0	0	0
0	0	0	0	0	0	0	0	0	0
0	0	0	0	0	0	0	0	1	1
0	0	0	0	0	0	0	0	0	0
0	0	0	0	0	0	0	0	0	0
0	0	0	0	0	0	0	0	1	0
0	0	0	0	0	0	0	0	0	0
0	0	0	0	0	0	0	0	1	1
0	0	0	0	0	0	0	0	0	0
0	0	0	0	0	0	0	0	0	0
0	0	0	0	0	0	0	0	0	0
0	0	0	0	0	0	0	0	0	0
1	0	0	0	0	0	0	0	0	0
0	0	0	0	0	0	0	0	0	0
0	0	0	0	0	0	0	0	0	0
0	0	0	0	0	0	0	0	0	0
0	0	0	0	0	0	0	0	0	0
0	0	0	0	0	0	0	0	0	0
0	0	0	0	0	0	0	0	0	0
0	0	0	0	0	0	0	0	1	0
0	0	0	0	0	0	0	0	0	0
0	0	0	0	0	0	0	0	1	1
0	0	0	0	0	0	0	0	1	0
0	0	0	0	0	0	0	0	0	0
0	0	0	0	0	0	0	0	0	0
0	0	0	0	0	0	0	0	0	0
0	0	0	0	0	0	0	0	0	0
0	0	0	0	0	0	0	0	1	0
0	0	0	0	0	0	0	0	0	0
0	0	0	0	0	0	0	0	0	0
0	0	0	0	0	0	1	0	0	0
0	0	0	0	0	0	0	0	0	0
0	0	0	0	0	0	0	0	0	0
0	0	0	0	0	0	0	0	0	0
0	0	0	0	0	0	0	0	1	0
0	0	0	0	0	0	0	0	1	0
0	0	0	0	0	0	0	0	1	0
0	0	0	0	0	0	0	0	0	0
0	0	0	0	0	0	0	0	0	0
0	0	0	0	0	0	0	0	0	0
0	0	0	0	0	0	1	0	0	0
0	0	0	0	0	0	0	0	0	0
0	0	0	0	0	0	0	0	0	0
0	0	0	0	0	0	0	0	0	0
0	0	0	0	0	0	0	0	0	0

0	0	0	0	0	0	0	0	0	0
0	0	0	0	0	0	0	0	0	0
0	0	0	0	0	0	0	0	0	0
0	0	0	0	0	0	0	0	0	0
0	0	0	0	0	0	1	0	0	0
0	0	0	0	0	0	0	0	1	0
0	0	0	0	0	0	0	0	0	0
0	0	0	0	0	0	0	0	0	0
0	0	0	0	0	0	0	0	1	0
0	0	0	0	0	0	0	0	0	0
0	0	0	0	0	0	0	0	1	1
0	0	0	0	0	0	0	0	0	0
0	0	0	0	0	0	0	0	1	0
0	0	0	?	0	0	0	0	0	0
0	0	0	0	0	0	0	0	0	0
0	1	0	0	0	0	0	0	0	0
0	0	0	0	0	0	0	0	0	0
0	0	0	0	0	0	0	0	1	1
0	0	0	0	0	0	0	0	0	0
0	0	0	0	0	0	0	0	0	0
0	0	0	0	0	0	0	0	1	0
0	0	0	0	0	0	0	0	1	1
0	0	0	0	0	0	0	0	0	0
0	0	0	0	0	0	0	0	0	0
1	0	0	0	0	0	0	0	0	0
0	0	0	0	0	0	0	0	0	0
0	0	0	0	0	0	0	0	0	0
0	0	0	0	0	0	0	0	0	0
0	0	0	0	0	0	0	0	0	0
0	0	0	0	0	0	0	0	1	0
0	0	0	0	0	0	0	0	0	0
0	0	0	0	0	0	0	0	0	0
0	0	0	0	0	0	0	0	0	0
0	1	0	0	0	0	0	0	0	0
0	0	0	0	0	0	0	0	0	0
0	0	0	0	0	0	0	0	1	0
0	0	0	0	0	0	0	0	0	0
0	0	0	0	0	0	0	0	1	1
0	0	0	0	0	0	0	0	1	0
0	0	0	0	0	0	0	0	0	0
0	0	0	0	0	0	0	0	1	0
0	0	0	0	0	0	0	0	0	0
0	0	0	0	0	0	0	0	0	0
0	0	0	0	0	0	0	0	1	0
0	0	0	0	0	0	0	0	0	0
0	0	0	0	0	0	0	0	1	0
0	0	0	0	0	0	0	0	0	0
0	0	0	0	0	0	0	0	0	0
0	0	0	0	0	0	0	0	0	0
0	0	0	0	0	0	0	0	0	0
0	0	0	0	0	0	0	0	1	0
0	0	0	0	0	0	0	0	0	0
0	0	0	0	0	0	0	0	1	0
0	0	0	0	0	0	0	0	1	0
0	0	0	0	0	0	0	0	0	0
0	0	0	0	0	0	0	0	0	0
0	0	0	0	0	0	0	0	0	0
0	0	0	0	0	0	0	0	0	0
0	0	0	0	0	1	0	0	0	0
0	0	0	0	0	0	0	0	0	0
0	0	0	0	0	0	0	0	1	0
0	0	0	0	0	0	0	0	0	0
0	0	0	0	0	0	0	0	0	0
0	0	0	0	0	0	0	0	0	0
0	0	0	0	0	0	1	0	0	0
0	0	0	0	0	0	0	0	0	0
0	0	0	0	0	0	0	0	1	0
0	0	0	0	0	0	0	0	1	0
0	1	0	0	0	0	0	0	0	0
0	0	0	?	0	0	0	0	0	0
0	0	0	0	0	0	0	0	0	0
0	0	0	0	0	0	0	0	0	0
0	0	0	0	0	0	0	0	1	0
0	0	0	0	0	0	0	0	0	0
0	0	0	0	0	0	0	0	1	0
0	0	0	0	0	0	0	0	1	0
0	0	0	0	0	0	0	0	1	0

0	0	0	0	0	0	0	0	0	0
1	0	0	0	0	0	0	0	0	0
0	0	0	0	0	0	0	1	0	0
0	0	0	0	0	0	0	0	1	0
0	0	0	0	0	0	0	0	0	0
0	0	0	0	0	0	0	0	0	0
0	0	0	0	0	1	0	0	0	0
0	0	0	0	0	0	0	0	0	0
0	0	0	0	0	0	1	0	0	0
0	0	0	0	0	0	0	0	0	0
0	0	0	0	0	0	0	0	0	0
0	0	0	0	0	0	0	0	1	1
0	0	0	0	0	0	1	0	0	0
0	0	0	0	0	0	0	0	0	0
0	0	0	0	0	0	0	0	1	0
0	0	0	0	0	0	0	0	1	0
0	0	0	0	0	0	0	0	0	0
0	0	0	0	0	0	0	0	0	0
0	0	1	0	0	0	0	0	0	0
0	0	0	0	0	0	0	0	0	0
0	0	0	0	0	0	0	0	1	0
0	0	1	0	0	0	0	0	0	0
0	0	0	0	0	0	0	0	1	0
0	0	0	0	0	0	0	0	0	0
0	0	0	0	0	0	0	0	0	0
0	0	0	0	0	0	0	0	0	0
0	0	0	0	0	0	0	0	0	0
0	0	0	0	0	0	0	0	0	0
0	0	0	0	0	0	0	0	0	0
0	0	0	0	1	0	0	0	0	0
?	0	0	0	0	0	0	0	0	0
0	0	0	0	0	0	0	0	1	0
0	0	0	0	0	0	0	0	0	0
0	0	0	0	0	0	0	0	0	0
0	0	0	0	0	0	0	0	0	0
0	0	0	0	0	0	0	0	0	0
0	0	0	0	0	0	0	0	0	0
0	0	0	0	0	0	0	0	1	0
0	0	0	0	0	0	0	0	0	0
0	0	0	0	0	0	0	0	1	0
0	0	0	0	0	0	0	0	1	0
0	0	0	0	0	0	0	0	1	1
0	0	0	0	0	1	0	0	0	0
0	0	0	0	0	0	0	0	1	0
0	0	0	0	0	0	0	0	1	0
0	0	0	0	0	0	0	0	0	0
0	0	0	0	0	0	0	0	0	0
0	0	0	0	0	0	0	0	0	0
0	0	0	0	0	0	0	0	0	0
0	0	0	0	0	0	0	0	0	0
0	0	0	0	0	0	0	0	0	0
0	0	0	0	0	0	0	0	0	0
0	0	0	0	0	0	0	0	0	0
0	0	0	0	0	0	0	0	1	0
0	0	0	0	0	0	0	0	1	1
0	0	0	0	0	0	0	0	0	0
0	0	0	0	0	0	0	0	1	1
0	0	0	0	0	0	0	0	1	0
0	0	0	0	0	0	0	0	1	0
?	0	0	0	0	0	0	0	0	0
0	0	0	0	0	0	1	0	0	0
0	0	0	0	0	0	0	0	1	0
0	1	0	0	0	0	0	0	0	0
0	0	0	0	0	0	0	0	1	0
0	0	0	0	0	0	0	0	0	0
0	0	0	0	0	1	0	0	0	0
0	0	0	0	0	0	0	0	0	0
0	0	0	0	0	0	0	0	0	0
0	0	0	0	0	0	0	0	0	0
0	0	0	0	0	0	0	0	1	1
0	0	0	0	0	0	0	0	0	0
0	0	0	0	0	0	1	0	0	0
0	0	0	0	0	0	0	0	0	0
0	0	0	0	1	0	0	0	0	0
0	0	0	0	0	1	0	0	0	0
0	0	0	0	0	0	1	0	0	0
0	0	0	0	0	0	0	0	0	0

0	0	0	0	0	0	0	0	0	0
0	0	0	0	0	0	1	0	0	0
0	0	0	0	0	0	0	0	1	0
0	0	0	0	0	0	0	0	0	0
0	0	0	0	0	0	1	0	0	0
0	0	0	0	0	0	1	0	0	0
0	0	0	0	0	0	0	0	0	0
?	0	0	0	0	0	0	0	0	0
0	0	0	0	0	0	0	0	0	0
0	0	0	0	0	0	0	0	0	0
0	0	0	0	0	0	0	0	1	0
0	0	0	0	0	0	0	0	1	0
0	0	0	0	0	0	1	0	0	0
0	0	0	0	0	0	0	0	0	0
0	0	0	0	0	0	0	0	1	1
0	0	0	0	0	0	0	0	0	0
0	0	0	0	0	0	0	0	0	0
0	0	0	0	0	0	0	0	0	0
0	0	0	0	0	0	0	0	0	0
0	0	0	0	0	0	0	0	1	0
0	0	0	0	0	0	0	0	1	0
0	0	0	0	0	0	0	0	0	0
0	0	0	0	0	0	0	0	0	0
0	0	0	0	0	0	0	0	0	0
0	0	0	0	0	0	0	0	0	0
0	0	0	0	0	0	0	0	1	0
0	0	0	0	0	0	0	0	0	0
0	0	0	0	0	0	0	0	0	0
0	0	0	0	0	0	0	0	1	0
0	0	0	0	0	0	0	0	0	0
0	0	0	0	0	0	0	0	0	0
0	0	0	0	0	0	0	0	0	0
0	0	0	0	0	1	0	0	0	0
0	0	0	0	0	0	0	0	0	0
0	0	0	0	0	0	0	0	0	0
0	0	0	0	0	0	0	0	0	0
0	0	0	0	0	0	1	0	0	0
0	0	0	0	0	0	0	0	0	0
0	0	0	0	0	0	0	0	1	0
0	1	0	0	0	0	0	0	0	0
0	0	0	0	0	0	0	0	0	0
0	0	0	0	0	0	0	0	1	0
0	0	0	0	0	0	0	0	0	0
0	0	0	0	0	0	0	0	1	0
0	0	0	0	0	0	0	0	1	1
0	0	0	0	0	0	0	0	1	1
0	0	0	0	0	0	0	0	0	0
0	0	0	0	0	0	0	0	1	0
0	0	0	0	0	0	0	0	0	0
0	0	0	0	0	0	0	0	0	0
1	0	0	0	0	0	0	0	0	0
0	0	0	0	0	0	0	0	0	0
0	0	0	0	0	0	0	0	1	0
0	0	0	0	0	0	0	0	0	0
0	0	0	0	0	0	0	0	0	0
0	0	0	0	0	0	0	0	0	0
0	0	0	0	0	0	0	0	0	0
0	0	0	0	0	0	0	0	1	0
0	0	0	0	0	0	0	0	0	0
0	0	0	0	0	0	0	0	1	0
0	0	0	0	0	0	0	0	0	0
0	0	0	0	0	0	0	0	0	0
0	0	0	0	0	0	0	0	0	0
0	0	0	0	0	0	0	0	0	0
0	0	0	0	0	0	0	0	0	0
0	0	0	0	0	0	0	0	0	0
0	0	0	0	0	0	0	0	0	0
0	0	0	0	0	0	0	0	0	0
0	0	0	0	0	0	0	0	0	0
0	0	0	0	0	0	0	0	0	0
0	0	0	0	0	0	0	0	1	0
0	0	0	0	0	0	0	0	0	0
0	0	0	0	0	1	0	0	0	0
0	0	0	0	0	0	0	0	0	0
0	0	0	0	0	0	0	0	0	0
0	0	0	0	0	0	0	0	0	0
0	0	0	0	0	0	0	0	0	0

0	0	0	0	0	0	0	0	0	0
0	0	0	0	0	0	1	0	0	0
0	0	0	0	0	0	0	0	0	0
0	0	0	0	0	0	0	0	0	0
0	0	0	0	0	1	0	0	0	0
0	0	0	0	0	0	0	0	0	0
0	0	0	0	0	0	0	0	0	0
0	0	0	0	0	0	0	0	0	0
0	0	0	0	0	0	0	0	0	0
0	0	0	0	0	0	0	0	0	0
0	0	0	0	0	0	0	0	0	0
0	0	0	0	0	0	0	0	0	0
0	0	0	0	0	0	1	0	0	0
0	0	0	0	0	0	1	0	0	0
0	0	0	0	0	0	0	0	1	1
0	0	0	0	0	0	0	0	0	0
0	1	0	0	0	0	0	0	0	0
0	0	0	0	0	0	0	0	0	0
0	0	0	0	0	0	0	0	0	0
0	0	0	0	0	0	0	0	0	0
0	0	0	0	0	0	0	0	0	0
0	0	0	0	0	0	0	0	0	0
0	0	0	0	0	0	0	0	0	0
0	0	0	0	0	0	0	0	0	0
0	0	0	0	0	0	0	0	0	0
0	0	0	0	0	0	0	0	0	0
0	0	0	0	0	0	0	0	1	1
0	0	0	0	0	0	0	0	0	0
0	0	0	0	0	0	0	0	0	0
0	0	0	0	0	0	0	0	0	0
0	0	0	0	0	0	0	0	?	0
0	0	0	0	0	0	0	0	0	0
0	0	0	0	0	0	0	0	0	0
0	0	0	0	0	0	0	0	0	0
0	0	0	0	0	0	0	0	0	0
0	0	0	0	1	0	0	0	0	0
0	0	0	0	0	0	0	0	0	0
0	0	0	0	0	0	0	0	1	1
0	0	0	0	0	0	0	0	0	0
0	0	0	0	0	0	0	0	0	0
0	0	0	0	0	0	0	0	1	0
0	0	0	0	0	0	0	0	1	0
0	0	0	0	0	0	0	0	0	0
0	0	0	0	0	0	0	0	1	1
0	0	0	0	0	0	0	0	1	1
0	0	0	0	0	0	0	0	0	0
0	0	0	0	0	0	0	0	0	0
0	0	0	0	0	0	0	0	0	0
0	0	0	0	0	0	0	0	0	0
0	0	0	0	0	0	0	0	0	0
0	0	0	0	0	0	0	0	1	0
0	0	0	0	0	0	0	0	1	0
0	0	0	0	0	0	0	0	0	0
0	0	0	0	0	0	0	0	0	0
0	1	0	0	0	0	0	0	0	0
0	0	0	0	0	0	0	0	0	0
0	0	0	0	0	0	0	0	1	0
0	0	0	0	0	0	0	0	1	0
0	0	0	0	0	0	0	0	0	0
0	0	0	0	0	0	0	0	1	0
0	0	0	0	0	0	0	0	1	1
0	0	0	0	0	0	0	0	1	1
0	0	0	0	0	0	0	0	0	0
0	0	0	0	0	0	0	0	0	0
0	0	0	0	0	0	0	0	0	0
1	0	0	0	0	0	0	0	0	0
0	0	0	0	0	0	1	0	0	0
1	0	0	0	0	0	0	0	0	0
0	0	0	0	0	0	1	0	0	0
1	0	0	0	0	0	0	0	0	0
0	0	0	0	0	0	0	0	0	0
0	0	0	0	0	0	0	0	0	0
0	0	0	0	0	0	0	0	0	0
0	0	0	0	0	0	0	0	1	0
0	0	0	0	0	0	0	0	0	0
0	0	0	0	0	0	1	0	0	0
0	0	0	0	0	0	0	0	0	0

0	0	0	0	0	0	0	0	1	0
0	0	0	0	0	0	0	0	0	0
0	0	0	0	0	0	0	0	0	0
0	0	0	0	0	0	0	0	1	0
0	0	0	0	0	0	0	0	1	0
0	0	0	0	1	0	0	0	0	0
0	0	0	0	1	0	0	0	0	0
0	0	0	0	0	0	0	0	0	0
0	0	0	0	0	0	0	0	0	0
0	0	0	0	0	0	0	0	1	0
0	0	0	0	0	0	0	0	0	0
0	0	0	0	0	0	0	0	0	0
0	0	0	0	0	0	0	0	1	1
0	0	0	0	0	0	0	0	1	0
0	0	0	0	0	0	0	0	0	0
0	0	0	0	0	0	0	0	0	0
0	0	0	0	0	1	0	0	0	0
0	0	0	0	0	0	0	0	0	0
0	0	0	0	0	0	0	0	?	0
0	0	0	0	0	0	0	0	0	0
0	0	0	0	0	0	0	0	0	0
0	0	0	0	0	0	0	0	0	0
0	0	0	0	0	0	0	0	?	0
0	0	0	0	0	0	0	0	0	0
0	0	0	0	0	0	0	0	0	0
0	0	0	0	0	0	0	0	0	0
0	0	0	0	0	0	0	0	0	0
0	0	0	0	0	0	0	0	0	0
0	0	0	0	0	0	0	0	1	0
0	0	0	0	0	0	1	0	0	0
?	0	0	0	0	0	0	0	0	0
0	0	0	0	0	0	0	0	0	0
1	0	0	0	0	0	0	0	0	0
0	0	0	0	0	0	0	0	1	0
0	0	0	0	0	0	0	0	0	0
0	0	0	0	0	0	0	0	1	0
0	0	0	0	0	0	0	0	0	0
0	0	0	0	0	0	0	0	0	0
0	0	0	0	0	0	0	0	1	0
0	0	0	0	0	0	0	0	0	0
0	0	0	0	0	0	0	0	1	1
0	0	0	0	0	0	0	0	0	0
0	0	0	0	0	0	1	0	0	0
0	0	0	0	0	0	0	0	0	0
0	0	0	0	0	0	0	0	1	0
0	0	0	0	0	0	0	0	1	1
0	0	0	0	0	0	0	0	0	0
0	0	0	0	0	0	0	0	1	1
0	0	0	0	0	0	0	0	0	0
0	0	0	0	0	0	0	0	0	0
0	0	0	0	0	0	0	0	0	0
0	0	0	0	0	0	0	0	0	0
0	0	0	0	0	0	0	0	1	0
0	0	0	0	0	0	0	0	0	0
0	0	0	0	0	0	0	0	0	0
0	0	0	0	0	0	0	0	0	0
0	0	0	0	0	0	0	0	0	0
0	0	0	0	0	0	0	0	0	0
0	0	0	0	0	0	0	0	0	0
0	0	0	0	0	0	0	0	0	0
0	0	0	0	0	0	0	0	0	0
0	0	0	0	0	0	0	0	0	0
0	0	0	0	0	0	0	0	0	0
0	0	0	0	0	0	0	0	0	0
0	0	0	0	0	0	0	0	0	0
0	0	0	0	0	0	0	0	0	0
0	0	0	0	0	0	0	0	0	0
0	0	0	0	0	0	0	0	0	0
0	0	0	0	0	0	0	0	0	0
0	0	0	0	0	0	0	0	1	0
0	0	1	0	0	0	0	0	0	0
0	0	0	0	0	0	0	0	0	0
0	0	0	0	0	0	0	0	0	0
0	0	0	0	0	0	1	0	0	0
0	0	0	0	0	0	0	0	0	0
0	0	0	0	0	0	0	0	0	0

0	0	0	0	0	0	0	0	1	0
0	0	0	0	0	0	0	0	0	0
0	0	0	0	0	0	0	0	0	0
0	0	0	0	0	0	0	0	0	0
0	0	0	0	0	0	0	0	0	0
1	0	0	0	0	0	0	0	0	0
0	0	0	0	0	0	0	0	0	0
0	0	0	0	0	0	0	0	0	0
0	0	0	?	0	0	0	0	0	0
0	0	0	0	0	0	0	0	0	0
0	0	0	0	0	0	0	0	0	0
0	0	0	0	0	0	0	0	1	0
0	0	0	0	0	0	0	0	0	0
0	0	0	0	0	1	0	0	0	0
0	0	0	0	0	1	0	0	0	0
0	0	0	0	0	0	1	0	0	0
0	0	0	0	0	0	0	0	1	0
0	0	0	0	0	0	0	0	1	0
0	0	0	0	0	0	0	0	0	0
0	0	0	0	0	0	0	0	0	0
0	0	0	0	0	0	0	0	0	0
0	0	0	0	0	0	0	0	0	0
0	0	0	0	0	0	1	0	0	0
0	0	0	0	0	1	0	0	0	0
0	0	0	0	0	0	0	0	0	0
0	0	0	0	0	0	0	0	0	0
1	0	0	0	0	0	0	0	0	0
0	0	0	0	0	0	0	0	0	0
0	0	0	0	0	0	0	0	0	0
0	0	0	0	0	0	0	0	1	0
0	0	0	0	0	0	0	0	1	0
0	0	0	0	0	0	0	0	1	0
0	0	0	0	0	1	0	0	0	0
0	0	0	0	0	0	0	0	0	0
0	0	0	0	0	0	0	0	0	0
0	0	0	0	0	0	0	0	0	0
0	0	0	0	0	0	0	0	0	0
0	0	0	0	0	1	0	0	0	0
0	0	0	0	0	0	0	0	0	0
0	0	0	0	0	0	0	0	0	0
0	0	0	0	0	0	0	0	1	0
0	0	0	0	0	0	0	0	0	0
0	0	0	0	0	0	0	0	0	0
0	0	0	0	0	0	0	0	0	0
0	0	0	0	0	0	0	0	0	0
1	0	0	0	0	0	0	0	0	0
0	0	0	0	0	0	0	0	0	0
0	0	0	0	0	0	0	0	0	0
0	0	0	0	0	0	0	0	0	0
0	0	0	0	0	0	0	0	1	0
0	0	0	0	1	0	0	0	0	0
0	0	0	0	0	0	0	0	0	0
0	0	0	0	0	0	0	0	0	0
0	0	0	0	0	0	0	0	0	0
1	0	0	0	0	0	0	0	0	0
0	0	0	0	0	0	0	0	0	0
0	0	0	0	0	0	0	0	0	0
0	0	0	0	0	0	0	0	0	0
0	0	0	0	0	0	0	0	1	1
0	0	0	0	0	0	0	0	0	0
0	0	0	0	0	0	0	0	1	1
0	0	0	0	0	0	0	0	1	1
0	0	0	0	0	0	0	0	0	0
0	0	0	0	0	0	0	0	0	0
0	0	0	0	0	0	0	0	0	0
0	0	0	0	0	0	0	0	0	0
0	0	0	0	0	?	0	0	0	0
0	0	0	0	0	0	0	0	0	0
1	0	0	0	0	0	0	0	0	0
0	0	0	0	0	1	0	0	0	0
0	0	0	0	0	0	0	0	0	0
0	1	0	0	0	0	0	0	0	0
1	0	0	0	0	0	0	0	0	0
0	0	0	0	0	0	1	0	0	0
0	0	0	0	0	0	0	0	0	0
0	0	0	0	0	0	0	0	1	0
0	0	0	0	0	0	0	0	0	0

0	0	0	0	0	0	0	0	1	0
0	0	0	0	0	0	0	0	0	0
0	0	0	0	0	0	0	0	0	0
0	0	0	0	0	0	0	0	0	0
0	1	0	0	0	0	0	0	0	0
0	0	0	0	0	0	0	0	1	0
0	0	0	0	0	1	0	0	0	0
0	0	0	0	0	0	0	0	0	0
0	0	0	0	0	0	0	0	0	0
0	0	0	0	0	0	0	0	0	0
0	0	0	0	0	0	0	0	0	0
0	0	0	0	0	0	0	0	0	0
0	0	0	0	0	0	0	0	0	0
0	0	0	0	0	0	0	0	0	0
0	0	0	0	0	0	0	0	1	1
0	0	0	0	0	0	0	0	1	0
0	0	0	0	0	0	0	0	0	0
0	0	0	0	0	0	0	0	0	0
0	0	0	0	0	0	0	0	0	0
0	0	0	0	0	0	0	0	0	0
0	0	0	0	0	0	0	0	0	0
0	0	0	0	0	0	0	0	0	0
0	0	0	0	0	0	0	0	0	0
0	0	0	0	0	0	0	0	0	0
0	0	0	0	0	0	0	0	0	0
0	0	0	0	0	0	0	0	1	0
0	0	0	0	1	0	0	0	0	0
0	0	0	0	0	0	1	0	0	0
0	0	0	0	0	0	0	0	0	0
0	0	0	0	0	0	0	0	0	0
0	0	0	0	0	0	0	0	1	0
0	0	0	0	0	0	0	0	0	0
0	0	0	0	0	0	0	0	0	0
0	0	0	0	0	0	0	0	0	0
0	1	0	0	0	0	0	0	0	0
0	0	0	0	0	0	0	0	0	0
0	0	0	0	0	0	0	0	0	0
0	0	0	0	0	0	0	0	0	0
0	0	0	0	0	0	0	1	0	0
0	0	0	0	0	0	0	0	0	0
0	1	0	0	0	0	0	0	0	0
0	0	0	0	0	0	0	0	1	1
0	0	0	0	0	0	0	0	0	0
0	0	0	0	0	0	0	0	1	0
0	0	0	0	0	0	0	0	0	0
0	0	0	0	0	0	0	0	0	0
0	1	0	0	0	0	0	0	0	0
0	0	0	0	0	0	0	0	1	0
0	0	0	0	0	0	0	0	0	0
0	0	0	0	0	0	0	0	0	0
0	0	0	0	0	0	0	0	0	0
1	0	0	0	0	0	0	0	0	0
0	0	0	0	0	0	1	0	0	0
0	0	0	0	0	0	0	0	0	0
0	0	0	0	0	0	0	0	1	0
0	0	0	0	0	0	0	0	0	0
0	0	0	0	0	0	0	0	0	0
0	0	0	0	0	0	0	0	1	0
0	0	0	0	0	0	0	0	1	0
1	0	0	0	0	0	0	0	0	0
0	0	0	0	0	0	0	0	0	0
0	0	0	0	0	1	0	0	0	0
0	0	0	0	0	0	0	0	0	0
0	0	0	0	0	0	0	0	0	0
0	0	0	0	0	0	0	0	1	1
0	0	0	0	0	0	0	?	0	0
0	0	0	0	0	0	0	0	1	1
0	0	0	0	0	0	0	0	1	0
0	0	0	0	0	0	0	0	0	0
0	0	0	0	0	0	0	0	0	0
0	0	0	0	0	0	0	0	0	0
0	0	0	0	0	0	0	0	0	0
1	0	0	0	0	0	0	0	0	0
0	0	0	0	0	0	0	0	1	0
0	0	0	0	0	0	0	0	0	0
0	0	0	0	0	0	0	0	0	0
0	0	0	0	0	0	0	0	0	0

0	0	0 0	0 0	0 0	0	0
0	0	0 0	0 0	0 0	0	0
0	0	0 0	0 0	0 0	0	0
0	0	0 0	0 0	0 0	1	0
0	0	0 0	0 0	0 0	0	0
0	0	0 0	0 0	1 0	0	0
0	0	0 0	0 0	0 0	0	0
0	0	0 0	0 0	0 0	1	0
0	0	0 0	0 0	0 0	1	0
0	1	0 0	0 0	0 0	0	0
0	0	0 0	0 0	0 0	1	0
0	0	0 0	0 0	0 0	0	0
0	0	0 0	0 0	0 0	0	0
0	0	0 0	0 0	0 0	1	0
0	0	0 0	0 0	0 0	0	0
0	1	0 0	0 0	0 0	0	0
0	0	0 0	0 0	0 0	0	0
0	0	0 0	0 0	0 0	1	0
0	0	0 0	0 0	0 0	0	0
0	0	0 0	0 0	0 0	1	0
0	0	0 0	0 0	0 0	1	0
0	0	0 0	0 0	0 0	1	0
1	0	0 0	0 0	0 0	0	0
0	0	0 0	0 0	0 0	0	0
0	0	0 0	0 0	0 0	0	0
0	0	0 0	0 0	0 0	0	0
0	0	0 0	0 0	0 0	0	0
0	0	0 0	0 0	0 0	0	0
0	0	0 0	0 0	0 0	0	0
0	0	0 0	0 0	0 0	0	0
0	0	0 0	0 1	0 0	0	0
0	0	0 0	0 0	0 0	0	0
0	0	0 0	0 0	0 0	0	0
0	0	0 0	0 0	0 0	0	0
0	0	0 0	0 0	0 0	0	0
0	0	0 0	0 0	0 0	0	0
0	0	0 0	0 0	0 0	0	0
0	0	0 0	0 0	0 0	0	0
0	0	0 0	0 0	0 0	0	0
0	0	0 0	0 0	0 0	1	0
0	0	0 0	0 0	0 0	0	0
0	0	0 0	1 0	0 0	0	0
0	0	0 0	0 0	0 0	0	0
0	0	0 0	0 0	0 0	1	0
0	0	0 0	0 1	0 0	0	0
0	0	0 0	0 0	0 0	0	0
0	0	0 0	0 0	0 0	0	0
1	0	0 0	0 0	0 0	0	0
1	0	0 0	0 0	0 0	0	0
0	0	0 0	0 0	0 0	0	0
0	0	0 0	0 0	0 0	0	0
0	0	0 0	0 0	0 0	0	0
0	?	0 0	0 0	0 ?	0	0
0	0	0 0	0 0	0 0	0	0
0	0	0 0	0 1	0 0	0	0
0	0	0 0	0 0	0 0	0	0
0	0	0 0	0 0	0 0	0	0
0	0	0 0	0 0	0 0	0	0
0	0	0 0	0 0	0 0	0	0
0	0	0 0	1 0	0 0	0	0
0	0	0 0	0 1	0 0	0	0
1	0	0 0	0 0	0 0	0	0
0	0	0 0	0 0	0 0	1	0
0	0	0 0	0 0	0 0	1	0
0	0	0 0	0 0	0 0	0	0
0	0	0 0	0 0	0 0	1	0
0	0	0 0	0 0	0 0	1	0
0	0	0 0	0 0	0 0	0	0
0	0	0 0	0 0	0 0	0	0
0	0	0 0	0 0	0 0	0	0
0	0	0 0	0 0	0 0	0	0
0	0	0 0	0 0	0 0	1	0
0	0	0 0	0 0	0 0	1	0
0	0	0 0	0 0	0 0	1	1
0	0	0 0	0 0	0 0	1	1
0	0	0 0	0 0	0 0	0	0
0	0	0 0	0 0	0 0	0	0

0	0	0	0	0	0	0	0	0	0
0	0	0	0	0	0	0	0	1	1
0	0	0	0	0	0	0	0	0	0
0	0	0	0	0	0	0	0	0	0
0	0	0	0	0	0	0	0	0	0
0	0	0	0	0	0	0	0	0	0
0	0	0	0	0	0	0	0	0	0
0	0	0	0	0	0	0	0	0	0
1	0	0	0	0	0	0	0	0	0
0	0	0	0	0	0	0	0	0	0
0	0	0	0	0	0	0	0	0	0
0	0	0	0	0	0	0	0	1	1
0	0	0	0	0	0	1	0	0	0
0	0	0	0	0	0	0	0	0	0
0	0	0	0	0	0	0	0	0	0
0	0	0	0	0	0	0	0	0	0
0	0	0	0	0	0	0	0	0	0
0	0	0	0	0	0	0	0	0	0
0	0	0	0	0	0	0	0	0	0
0	0	0	0	0	0	0	0	0	0
0	0	0	0	0	0	0	1	0	0
0	0	0	0	0	0	0	0	0	0
0	0	0	0	0	0	0	0	0	0
0	0	0	0	0	0	0	0	0	0
0	0	0	0	0	0	0	0	0	0
0	0	0	0	0	1	0	0	0	0
0	0	0	0	0	0	0	0	0	0
0	0	0	0	0	0	0	0	0	0
0	0	0	0	0	0	0	0	0	0
0	0	0	0	0	0	0	0	0	0
0	0	0	0	0	0	0	0	0	0
0	0	0	0	0	0	0	0	1	0
0	0	0	0	0	0	0	0	1	0
0	0	0	0	0	0	0	0	0	0
0	0	0	0	0	0	0	0	0	0
0	0	0	0	0	0	0	0	0	0
0	0	0	0	0	0	0	0	0	0
0	0	0	0	0	0	0	0	0	0
0	0	0	0	0	0	0	0	1	0
0	0	0	?	0	0	0	0	0	0
0	0	0	0	0	0	0	0	0	0
0	0	0	0	0	0	0	0	1	1
0	0	0	0	0	0	0	0	1	1
0	0	0	0	0	0	1	0	0	0
0	0	0	0	0	0	0	0	0	0
0	0	0	0	0	0	0	0	0	0
0	0	0	0	0	0	0	0	1	1
0	0	0	0	0	0	0	0	0	0
0	0	0	0	0	0	0	0	0	0
0	0	0	0	0	0	0	0	0	0
0	0	0	0	0	0	0	0	0	0
0	0	0	0	0	0	0	0	0	0
0	0	0	0	0	0	0	0	1	0
0	0	0	0	0	0	0	0	1	0
0	0	0	0	0	0	0	0	0	0
0	0	0	0	0	0	0	0	0	0
0	0	0	0	0	0	0	0	0	0
0	0	0	0	0	0	0	0	0	0
0	0	0	0	0	0	0	0	0	0
0	0	0	0	0	0	0	0	1	1
0	0	0	0	0	0	0	0	1	0
?	0	0	0	0	0	0	0	0	0
0	0	0	0	0	?	0	0	0	0
0	0	0	0	0	0	0	0	0	0
0	0	0	0	0	0	1	0	0	0
0	0	0	0	0	0	0	0	0	0
0	0	0	0	0	0	0	0	1	0
0	0	0	0	0	0	1	0	0	0
0	0	0	0	0	0	0	0	0	0
0	0	0	0	0	0	0	0	0	0
0	0	0	0	0	0	0	0	0	0
0	0	0	0	0	0	0	0	0	0
0	0	0	0	0	0	0	0	0	0
0	0	0	0	0	0	0	0	0	0
1	0	0	0	0	0	0	0	0	0
0	0	0	1	0	0	0	0	0	0
0	0	0	0	0	0	0	0	0	0

0	1	0	0	0	0	0	0	0	0
0	0	0	0	0	0	0	0	0	0
0	0	0	0	0	0	0	0	0	0
0	0	0	0	0	0	0	0	0	0
0	0	0	0	0	0	1	0	0	0
0	0	0	0	0	0	0	0	0	0
0	0	0	0	0	0	0	0	1	0
0	0	0	0	0	0	0	0	0	0
0	0	0	0	0	1	0	0	0	0
0	0	0	0	0	1	0	0	0	0
0	0	0	0	0	0	0	0	0	0
0	0	0	0	0	0	0	0	0	0
0	0	0	0	0	0	0	0	0	0
0	0	0	0	0	0	0	0	1	1
0	0	0	0	0	0	0	0	0	0
0	0	0	0	0	0	0	0	1	1
0	0	0	0	0	0	0	0	0	0
0	1	0	0	0	0	0	0	0	0
0	0	0	0	0	0	0	0	0	0
0	0	0	0	0	0	0	0	1	0
0	0	0	0	0	0	0	0	1	0
0	0	0	0	0	0	0	0	0	0
0	0	0	0	0	0	0	0	0	0
0	0	0	0	0	0	0	0	0	0
0	0	0	0	0	0	0	0	0	0
0	0	0	0	0	0	0	0	0	0
0	0	0	0	0	0	0	0	1	0
0	0	0	0	0	0	0	0	0	0
0	0	0	0	0	1	0	0	0	0
0	0	0	0	0	0	0	0	0	0
0	0	0	0	0	0	0	0	1	0
0	0	0	0	0	0	0	0	1	0
0	0	0	0	0	0	0	0	0	0
0	0	0	0	0	0	0	0	0	0
0	0	0	0	0	0	0	0	1	1
0	0	0	0	0	0	0	0	0	0
0	0	0	0	0	0	0	0	0	0
0	0	0	0	0	0	0	0	0	0
0	0	0	0	0	0	0	0	0	0
0	0	0	0	0	0	1	0	0	0
0	0	0	0	0	0	0	0	0	0
0	0	0	0	0	0	0	0	0	0
0	1	0	0	0	0	0	0	0	0
1	0	0	0	0	0	0	0	0	0
0	0	0	0	0	0	0	0	0	0
0	0	0	0	0	0	0	0	1	0
0	0	0	0	0	0	0	0	0	0
0	0	0	0	0	0	0	0	0	0
0	0	0	0	0	0	0	0	0	0
0	0	0	0	0	0	0	0	1	1
0	0	0	0	0	0	0	0	0	0
0	0	0	0	0	0	0	0	0	0
0	0	0	0	0	0	0	0	0	0
0	0	0	0	0	0	0	0	0	0
1	0	0	0	0	0	0	0	0	0
0	0	0	0	0	0	0	0	0	0
0	0	0	0	0	0	0	0	0	0
0	0	0	0	0	0	0	0	0	0
0	0	0	0	0	0	0	0	0	0
0	0	0	0	0	0	0	0	0	0
0	0	0	0	0	0	0	0	0	0
0	0	0	0	0	0	0	0	0	0
0	0	0	0	0	0	0	0	0	0
0	0	0	0	0	0	0	0	0	0
0	0	0	0	0	0	0	0	0	0
0	0	0	0	0	0	0	0	0	0
0	0	0	0	0	0	0	0	1	1
0	0	0	0	0	0	0	0	0	0
0	0	0	0	0	0	1	0	0	0
0	0	0	0	0	0	0	0	0	0
0	0	0	0	0	0	0	0	0	0
0	0	0	0	0	0	0	0	?	0
0	0	0	0	0	0	0	0	0	0
0	0	0	0	0	0	0	0	0	0
0	0	0	0	0	0	0	0	0	0
0	0	0	0	0	0	0	0	0	0
0	0	0	0	0	1	0	0	0	0

1	0	0	0	0	0	0	0	0	0
0	0	0	0	0	0	0	0	0	0
0	0	0	0	0	0	0	0	0	0
0	0	0	0	0	0	0	0	0	0
0	0	0	0	0	0	0	0	0	0
?	0	0	0	0	0	0	0	0	0
0	0	0	0	0	0	0	0	0	0
0	0	0	0	0	0	0	0	0	0
0	0	0	0	0	0	0	0	1	0
0	0	0	0	0	0	1	0	0	0
0	1	0	0	0	0	0	0	0	0
0	0	0	0	0	0	0	0	0	0
0	0	0	0	0	0	0	0	0	0
0	0	0	0	0	0	0	0	0	0
0	0	0	0	0	0	0	0	0	0
0	0	0	0	0	0	0	0	1	0
0	1	0	0	0	0	0	0	0	0
0	0	0	0	0	0	0	0	0	0
0	1	0	0	0	0	0	0	0	0
0	0	0	0	0	0	0	0	0	0
0	0	0	?	0	0	0	0	0	0
0	0	0	0	0	0	0	0	0	0
0	0	0	0	0	0	0	0	0	0
0	0	0	0	0	0	0	0	0	0
0	0	0	0	0	0	0	0	1	0
0	0	0	0	0	0	0	0	0	0
0	0	0	0	0	0	0	0	1	0
0	0	0	0	0	0	0	0	0	0
0	0	0	0	0	0	0	0	0	0
0	0	0	0	0	0	0	0	1	0
0	0	0	0	0	0	0	0	0	0
0	0	0	0	0	0	0	0	0	0
0	0	0	0	0	0	0	0	0	0
0	0	0	0	0	0	0	0	0	0

Gewalt_A	Schnittverletzung	DistorsionKontusion	Verkehrsunfall	Suizidal	Selbstverletzung
0	0	0	0	0	0
0	0	0	0	0	0
0	0	0	0	0	0
0	0	0	0	0	0
0	0	0	0	0	0
0	0	0	0	0	0
0	0	0	0	0	0
0	0	0	0	0	0
0	0	0	0	0	0
0	0	0	0	0	0
0	0	0	0	0	0
0	0	0	0	0	0
0	0	0	0	0	0
0	0	0	0	0	0
0	0	0	0	0	0
0	0	0	0	0	0
0	0	0	0	0	0
0	0	0	0	0	0
0	0	0	0	0	0
0	0	0	0	0	0
0	0	0	0	0	0
0	0	0	0	0	0
0	0	1	0	0	0
0	0	0	0	0	0
0	0	0	0	0	0
0	0	0	0	0	0
0	0	0	0	0	0
0	0	0	0	0	0
0	0	0	0	0	0
0	0	0	0	0	0
0	0	0	0	0	0
0	0	0	0	0	0
0	0	0	0	0	0
0	0	0	0	0	0
0	0	0	0	0	0
0	0	0	0	0	0
0	0	0	0	0	0
0	0	0	0	0	0
0	0	0	0	0	0
0	0	0	0	0	0
0	0	0	0	0	0
0	0	0	0	0	0
0	0	0	0	0	0
0	0	0	0	0	0
0	0	0	0	0	0
0	0	0	0	0	0
0	0	0	0	0	0
0	0	0	0	0	0
0	0	0	0	0	0
0	0	0	0	0	0
1	0	0	0	0	0
0	0	0	0	0	0
0	0	0	0	0	0
0	0	1	0	0	0
0	0	0	0	0	0
0	0	0	0	0	0
0	0	0	0	0	0
0	0	0	0	0	0
0	0	1	0	0	0
0	0	0	0	0	0
0	0	0	0	0	0
0	0	0	0	0	0
0	0	0	0	0	0
0	0	0	0	0	0
0	0	0	0	0	0
1	0	0	0	0	0
1	0	0	0	0	0
0	1	0	0	0	0
0	0	0	0	0	0
0	0	0	0	0	0
0	0	0	0	0	0
0	0	0	0	0	0
0	0	0	0	0	0
0	0	0	0	0	0
0	0	0	0	0	0
0	0	0	0	0	0

0	0	0	0	0	0
0	0	0	0	0	0
0	0	0	0	0	0
0	0	0	0	0	0
0	0	0	0	0	0
1	0	0	0	0	0
0	0	0	0	0	0
0	0	0	0	0	0
1	0	0	0	0	0
0	0	0	0	0	0
0	0	0	0	0	0
0	0	0	0	0	0
0	0	0	1	0	0
0	0	0	0	0	0
0	0	0	0	0	0
0	0	0	0	0	0
0	0	0	0	0	0
0	0	0	0	0	0
0	0	0	0	0	0
0	0	0	0	0	0
1	0	0	0	0	0
0	0	0	0	0	0
0	0	0	0	0	0
0	0	0	0	0	0
0	0	0	0	0	0
0	0	0	0	0	0
0	0	0	0	0	0
0	0	0	0	0	0
0	0	0	0	0	0
0	0	0	1	0	0
0	0	0	0	0	0
0	0	0	0	0	0
0	0	0	0	0	0
0	0	0	0	0	0
0	0	0	0	0	0
0	0	1	0	0	0
0	0	0	0	0	0
0	0	0	0	0	0
0	0	0	1	0	0
0	0	0	0	0	0
0	0	1	0	0	0
0	0	0	0	0	0
0	0	0	0	0	0
0	0	1	0	0	0
0	0	0	0	0	0
1	0	0	0	0	0
0	0	0	0	0	0
0	0	0	0	0	0
0	0	0	0	0	0
0	0	0	0	0	0
0	1	0	0	0	0
0	0	0	0	0	0
1	0	0	0	0	0
0	0	0	1	0	0
0	0	0	0	0	0
0	0	0	0	0	0
0	0	0	0	0	0
0	0	0	0	0	0
0	0	0	0	0	0
0	0	0	0	0	0
0	0	0	0	0	0
0	0	0	0	0	0
0	0	0	0	0	0
0	0	0	0	0	0
0	0	0	0	0	0
0	0	0	0	0	0
0	0	0	0	0	0
0	0	1	0	0	0
0	0	0	0	0	0
0	0	0	0	0	0
0	0	0	0	0	0
0	0	0	0	0	0
0	0	0	0	0	0
0	0	0	0	0	0
0	0	0	0	0	1
0	0	1	0	0	0
0	0	1	0	0	0

0	0	0	0	0	0
0	0	0	0	0	0
0	0	0	0	0	0
0	0	0	0	0	0
0	0	0	0	0	0
0	0	0	0	0	0
0	0	0	0	0	0
0	0	0	0	0	0
0	0	0	0	0	0
0	0	0	0	0	0
0	0	0	0	0	0
0	0	0	0	0	0
0	0	0	0	0	0
0	0	0	0	0	0
0	0	0	1	0	0
0	0	1	0	0	0
0	0	0	0	0	0
0	0	0	0	0	0
0	0	0	0	0	0
0	0	0	0	0	0
0	0	0	0	0	0
0	0	0	0	0	0
0	0	1	0	0	0
0	0	0	0	0	0
0	0	0	0	0	0
0	0	0	0	0	0
0	0	0	0	0	0
0	0	0	0	0	0
0	0	0	0	0	0
0	0	0	0	0	0
0	0	0	0	0	0
0	0	0	0	0	0
0	0	0	0	0	0
0	0	0	0	0	0
0	0	0	0	0	0
0	0	0	0	0	0
0	0	0	0	0	0
0	0	0	0	0	0
1	0	0	0	0	0
0	0	1	0	0	0
0	0	0	0	0	0
0	0	0	0	0	0
0	0	1	0	0	0
0	0	0	0	0	0
0	0	0	0	0	0
0	0	0	0	0	0
0	0	0	0	0	0
0	0	0	0	0	0
0	0	0	0	0	0
0	0	0	0	0	0
0	0	0	0	0	0
0	0	0	0	0	0
1	0	0	0	0	0
0	0	0	0	0	0
0	0	0	0	0	0
0	0	0	0	0	0
0	0	0	0	0	0
0	1	0	0	0	0
0	0	0	0	0	0
0	0	0	0	0	0
1	0	0	0	0	0
0	0	0	0	0	0
0	0	0	0	0	0
0	0	0	0	0	0
0	0	0	0	0	0
0	0	0	0	0	0
0	0	0	0	0	0
0	0	0	0	0	0
0	0	0	0	0	0
0	0	0	0	0	0
0	0	0	0	0	0
0	0	0	0	0	0
0	0	0	0	0	0
0	0	0	0	0	0
0	0	0	0	0	0
0	0	0	0	0	0
0	0	0	0	0	0

0	0	0	0	0	0
0	0	0	0	0	0
0	0	0	0	0	0
0	0	0	0	0	0
0	0	0	0	0	0
0	0	0	0	0	0
0	0	0	0	0	0
0	0	0	0	0	0
0	0	0	0	0	0
0	0	0	0	0	0
1	0	0	0	0	0
0	0	0	0	0	0
0	0	0	0	0	0
0	0	0	0	0	0
0	0	0	0	0	0
0	0	0	0	0	0
0	0	0	0	0	0
0	0	0	0	0	0
0	0	0	0	0	0
0	0	0	0	0	1
0	0	0	1	0	0
0	0	0	0	0	0
0	0	0	0	0	0
0	0	0	0	0	0
0	0	0	0	0	0
0	0	0	1	0	0
0	0	0	0	0	0
0	0	0	0	0	0
0	0	1	0	0	0
0	0	0	0	0	0
0	0	0	0	0	0
0	0	0	0	0	0
0	0	0	0	0	0
0	0	0	0	0	0
0	0	0	0	0	0
0	0	0	0	0	0
0	0	0	0	0	0
0	0	0	0	0	0
0	0	1	0	0	0
0	0	0	0	0	0
0	0	0	0	0	0
1	0	0	0	0	0
0	0	0	0	0	0
0	0	0	0	0	0
0	0	0	0	0	0
0	0	0	0	0	0
0	0	0	0	0	0
0	0	0	0	0	0
0	0	0	0	0	0
0	0	0	0	0	0
0	0	0	0	0	0
0	0	0	0	0	0
0	0	0	1	0	0
0	0	0	0	0	0
0	0	0	0	0	0
0	0	0	0	0	0
0	0	0	0	0	0
0	0	0	1	0	0
0	0	0	0	0	0
1	0	0	0	0	0
0	0	0	0	0	0
0	0	0	0	0	0
0	0	0	0	0	0
0	0	0	0	0	0
0	0	0	0	0	0
0	0	0	0	0	0
0	0	0	0	0	0
0	0	0	0	0	0
0	0	0	0	0	0
0	0	0	0	0	0
1	0	0	0	0	0
0	0	0	0	0	0
0	0	0	0	0	0
0	0	0	0	0	0
0	0	0	0	0	0
0	0	0	0	0	0
0	0	0	0	0	0

0	0	0	0	0	0
0	0	0	0	0	0
0	0	0	0	0	0
0	0	0	0	0	0
0	0	0	0	0	0
0	0	0	0	0	0
0	0	0	0	0	0
0	0	0	0	0	0
0	0	0	0	0	0
0	0	0	0	0	0
0	0	0	0	0	0
0	0	0	0	0	0
0	0	0	0	0	0
0	0	0	0	0	0
0	0	0	0	0	0
0	0	0	0	0	0
0	0	0	0	0	0
0	0	0	0	0	0
0	0	0	0	0	0
0	0	0	0	0	0
0	0	0	0	0	0
0	0	0	0	0	0
0	0	0	0	0	0
0	0	0	0	0	0
0	0	0	0	0	0
0	0	0	0	0	0
0	0	0	0	0	0
0	0	0	0	0	0
0	0	0	0	0	0
0	0	0	0	0	0
0	0	0	0	?	0
0	0	0	0	0	0
0	0	0	0	0	0
0	0	0	0	0	0
0	0	0	0	0	0
0	0	0	0	0	0
0	0	0	0	0	0
0	0	0	0	0	0
0	0	0	0	0	0
0	0	0	0	0	0
0	1	0	0	0	0
1	0	0	0	0	0
0	0	0	0	0	0
0	0	0	0	0	0
0	0	0	0	0	0
0	0	0	0	0	0
0	0	0	0	0	0
0	0	0	0	0	0
0	0	0	0	0	0
0	0	0	0	0	0
0	0	1	0	0	0
1	0	0	0	0	0
0	0	0	0	0	0
0	0	0	0	0	0
0	0	0	0	0	0
0	0	0	0	0	0
0	1	0	0	0	0
0	0	0	0	0	0
0	0	0	0	0	0
0	1	0	0	0	0
0	0	0	0	0	0
0	0	0	0	0	0
0	0	0	0	0	0
0	0	0	0	0	0
0	0	0	0	0	0
0	0	0	0	0	0
0	0	0	0	0	0
0	0	0	0	0	0
0	0	0	0	0	0
0	0	0	0	0	0
0	0	0	0	0	0
0	0	0	0	0	0
0	0	0	0	0	0
0	0	1	0	0	0
0	0	0	0	0	0
0	0	0	0	0	0
0	0	0	0	0	0

0	0	1	0	0	0
0	0	0	0	0	0
0	0	0	0	0	0
0	0	0	1	0	0
0	0	0	1	0	0
0	0	0	0	0	0
0	0	0	0	0	0
0	0	0	0	0	0
0	0	0	0	0	0
0	0	0	0	0	1
0	0	0	0	0	0
0	0	0	0	0	0
0	0	0	0	0	0
1	0	0	0	0	0
0	0	0	0	0	0
0	0	0	0	0	0
0	0	0	0	0	0
0	0	0	0	0	0
0	0	0	0	0	0
0	0	0	0	0	0
0	0	0	0	0	0
0	0	0	0	0	0
0	0	0	0	0	0
0	0	0	0	0	0
0	0	0	0	0	0
0	0	0	0	0	0
0	0	0	0	0	0
0	0	0	0	0	0
1	0	0	0	0	0
0	0	0	0	0	0
0	0	0	0	0	0
0	0	0	0	0	0
0	0	0	0	0	0
1	0	0	0	0	0
0	0	0	0	0	0
0	1	0	0	0	0
0	0	0	0	0	0
0	0	0	0	0	0
0	0	0	0	0	0
0	0	0	0	0	0
0	0	0	0	0	0
0	0	0	0	0	0
0	0	0	0	0	0
0	0	0	0	0	0
0	0	0	0	1	0
0	0	0	0	0	0
0	0	0	0	0	0
0	0	0	0	0	0
0	0	0	0	0	0
0	0	0	0	0	0
0	0	0	0	0	0
0	0	0	0	0	0
1	0	0	0	0	0
0	0	0	0	0	0
0	0	0	0	0	0
0	0	0	0	0	0
0	0	0	0	0	0
0	0	0	0	0	0
0	0	0	0	0	0
0	0	0	0	0	0
0	0	0	0	0	0
0	0	0	0	0	0
0	0	0	0	0	0
0	0	0	0	0	0
0	0	0	0	0	0
0	0	0	0	0	0
0	0	0	0	0	0
0	0	0	0	0	0
0	0	0	0	0	0
0	0	0	0	0	0
1	0	0	0	0	0
0	0	0	0	0	0
0	0	0	0	0	0
0	0	0	0	0	0
0	0	0	0	0	0
0	0	0	0	0	0
0	0	0	0	0	0

0	1	0	0	0	0
0	0	0	0	0	0
0	0	0	0	0	0
0	0	0	0	0	0
0	0	0	0	0	0
0	0	0	0	0	0
0	0	0	0	0	0
0	0	0	0	0	0
0	0	0	0	0	0
0	0	0	0	0	0
0	0	0	0	0	0
0	0	1	0	0	0
0	0	0	0	0	0
0	0	0	0	0	0
0	0	0	0	0	0
0	0	0	0	0	0
0	0	1	0	0	0
0	0	0	0	0	0
0	0	0	0	0	0
0	0	0	0	0	0
0	0	0	0	0	0
0	0	0	0	0	0
0	0	0	0	0	0
0	0	0	0	0	0
0	0	0	0	0	0
0	0	0	0	0	0
0	0	0	0	0	0
0	0	0	0	0	0
0	0	0	0	0	0
0	0	1	0	0	0
0	0	1	0	0	0
0	0	1	0	0	0
0	0	0	0	0	0
0	0	0	0	0	0
0	0	0	0	0	0
0	0	0	0	0	0
0	0	0	0	0	0
0	0	0	0	0	0
0	0	0	0	0	0
0	0	0	0	0	0
1	0	0	0	0	0
0	0	0	0	0	0
0	0	0	0	0	0
0	0	0	0	0	0
0	0	0	0	0	0
0	0	0	0	0	0
0	0	0	0	0	0
0	0	0	0	0	0
0	0	0	0	0	0
1	0	0	0	0	0
0	0	0	0	0	0
0	0	0	0	0	0
0	0	0	0	0	0
0	0	0	0	0	0
0	0	0	0	0	0
0	0	0	0	0	0
0	0	0	0	0	0
0	0	0	0	0	0
0	0	0	0	0	0
0	0	0	0	0	0
0	0	0	0	0	0
0	0	0	0	0	0
0	0	0	0	0	0
0	0	0	0	0	0
0	0	0	0	0	0
0	0	0	0	0	0
0	0	0	0	0	0
0	0	0	0	0	0
0	0	0	0	0	0
0	0	0	0	0	0
0	0	0	0	0	0
0	0	0	0	0	0
0	0	0	0	0	0
0	0	0	0	0	0
0	0	0	0	0	0
1	0	0	0	0	0
0	0	0	0	0	0

0	0	1	0	0	0
0	0	0	0	0	0
0	0	0	0	0	0
0	0	0	0	0	0
0	0	0	0	0	0
0	0	0	0	0	0
0	0	0	0	0	0
0	0	0	0	0	0
0	0	0	0	0	0
0	0	0	0	0	0
0	0	0	0	0	0
0	0	0	0	0	0
0	0	0	0	0	0
0	0	0	0	0	0
0	0	0	0	0	0
0	0	1	0	0	0
0	0	0	0	0	0
0	0	0	0	0	0
0	0	0	0	0	0
0	0	0	0	0	0
0	0	0	0	0	0
0	0	0	0	0	0
0	0	0	0	0	0
0	0	0	0	0	0
0	0	0	0	0	0
0	0	0	0	0	0
0	0	0	0	0	0
0	0	0	0	0	0
0	0	0	0	0	0
0	0	0	0	0	0
0	0	1	0	0	0
0	0	0	0	0	0
0	0	0	0	0	0
0	0	0	0	0	0
0	0	0	0	0	0
0	0	0	0	0	0
0	0	0	0	0	0
0	0	0	0	0	0
0	0	0	0	0	0
0	0	0	0	0	0
0	0	0	0	0	0
0	0	0	0	0	0
0	0	0	0	0	0
1	0	0	0	0	0
0	0	0	0	0	0
0	0	0	0	0	0
0	0	0	0	0	0
0	0	1	0	0	0
0	0	0	0	0	0
0	0	0	0	0	0
0	0	0	0	0	0
0	0	0	0	0	0
0	0	0	0	0	0
0	0	0	0	0	0
0	0	0	0	0	0
0	0	0	0	0	0
0	0	0	0	0	0
0	0	1	0	0	0
1	0	0	0	0	0
0	0	0	0	0	0
0	0	0	0	0	0
0	0	0	0	0	0
0	0	0	0	0	0
0	0	0	0	0	0
0	0	0	0	0	0
0	0	0	0	0	0
0	0	0	0	0	0
0	0	0	0	0	1
0	0	0	0	0	0
0	0	0	0	0	0
0	0	0	0	0	0
0	0	0	0	0	0
0	0	0	0	0	0
0	0	1	0	0	0
0	0	0	0	0	0
0	0	0	0	0	0
0	0	0	0	0	0

0	0	0	0	0	0
0	0	0	0	0	0
0	0	0	0	0	0
0	0	0	0	0	0
0	0	0	0	0	0
0	0	0	0	0	0
0	0	0	0	0	0
1	0	0	0	0	0
0	0	0	1	0	0
0	0	0	0	0	0
0	0	0	0	0	0
0	0	0	0	0	0
0	0	0	0	0	0
0	1	0	0	0	0
0	0	0	0	0	0
0	0	0	0	0	0
0	0	0	0	0	0
0	0	0	0	0	0
0	0	0	0	0	0
0	0	0	0	0	0
0	0	0	1	0	0
0	1	0	0	0	0
0	0	0	0	0	0
0	0	0	0	0	0
0	0	0	0	0	0
0	0	0	0	0	0
0	0	0	0	0	0
0	0	0	0	0	0
0	0	0	0	0	0
0	0	0	0	0	0
0	0	0	0	0	0
0	0	0	0	0	0
0	0	0	0	0	0
0	0	0	0	0	0
0	0	0	0	0	0
0	0	0	0	0	0
0	0	0	0	0	0
0	0	0	0	0	0
0	0	0	0	0	0
0	1	0	0	0	0
0	0	0	0	0	0
0	0	0	0	0	0
0	0	0	0	0	0
0	0	1	0	0	0
0	0	0	0	0	0
0	0	0	0	0	0
0	0	0	0	0	0
0	0	0	0	0	0
0	0	0	0	0	0
0	0	0	0	0	0
0	0	0	0	0	0
0	0	0	0	0	0
0	0	0	0	0	0
0	0	0	0	0	0
0	0	0	0	0	0
0	0	0	0	0	0
0	0	0	0	0	0
0	0	0	0	0	0
0	0	0	0	0	0
0	0	0	0	0	0
0	0	0	0	0	0
0	0	0	0	0	0
0	0	0	0	0	0
1	0	0	0	0	0
0	0	0	0	0	0
1	0	0	0	0	0
0	0	1	0	0	0
0	0	0	0	0	0
0	0	0	0	0	0
0	0	0	0	0	0
0	0	0	0	0	0
1	0	0	0	0	0
1	0	0	0	0	0
0	0	0	0	0	0
0	0	0	0	0	0
0	0	0	0	0	0
0	0	0	0	0	0

0	0	0	0	0	0
0	0	0	0	0	0
0	0	0	0	0	0
0	0	0	0	0	0
0	0	0	0	0	0
0	0	0	0	0	0
0	0	0	0	0	0
0	0	0	0	0	0
0	0	0	0	0	0
0	0	0	0	0	0
0	0	0	0	0	0
0	0	0	0	0	0
0	0	0	0	0	0
0	0	0	0	0	0
0	0	0	0	0	0
0	0	0	0	0	0
0	0	0	0	0	0
0	0	0	0	0	0
0	0	0	0	0	0
0	0	0	0	0	0
0	0	0	0	0	0
0	0	0	0	0	0
0	0	0	0	0	0
0	0	0	0	0	0
0	0	0	0	0	0
0	0	0	0	0	0
0	0	0	0	0	0
0	0	0	0	0	0
0	0	0	0	0	0
0	0	0	0	0	0
0	0	0	0	0	0
0	0	0	0	1	0
1	0	0	0	0	0
0	0	0	0	0	0
0	0	0	0	0	0
0	0	0	0	0	0
0	0	0	0	0	0
0	0	0	0	0	0
1	0	0	0	0	0
0	0	0	0	0	0
0	0	0	0	0	0
0	0	0	0	0	0
0	0	0	0	0	0
0	0	0	0	0	0
0	0	0	0	0	0
0	0	0	0	0	0
0	0	0	0	0	0
0	0	0	0	0	0
0	0	0	0	0	0
0	0	0	0	0	0
0	0	0	0	0	0
0	0	0	0	0	0
0	0	0	0	0	0
0	0	0	1	0	0
0	0	0	0	0	0
0	0	0	0	0	0
0	0	0	0	0	0
0	0	0	0	0	0
0	0	0	0	0	0
0	0	0	0	0	0
0	0	0	0	0	1
0	0	0	0	0	0
0	0	0	0	0	0
0	0	0	0	0	0
0	0	0	0	0	0
0	0	0	0	0	0
1	0	0	0	0	0
0	0	0	0	0	0
0	0	0	0	0	0
0	0	0	0	0	0
0	0	0	0	0	0
0	0	0	0	0	0
0	0	0	0	0	0
0	0	0	0	0	0
0	0	0	0	0	0
0	0	0	0	0	0
0	0	0	0	0	0

0	0	0	0	0	0
0	0	0	0	0	0
0	0	0	0	0	0
0	0	0	0	0	0
0	0	0	0	0	0
0	0	0	0	0	0
0	0	0	0	0	0
0	0	0	0	0	0
0	0	0	0	0	0
0	0	0	0	0	0
0	0	0	0	0	0
0	0	0	0	0	0
0	0	0	0	0	0
0	0	0	0	0	0
0	0	0	0	0	0
0	0	0	0	0	0
0	0	0	0	0	0
0	0	0	0	0	0
0	0	0	0	0	0
0	0	1	0	0	0
1	0	0	0	0	0
0	0	0	0	0	0
0	0	0	0	0	0
0	0	0	0	0	0
0	0	0	0	0	0
0	0	0	0	0	0
0	0	0	0	1	0
0	0	0	0	0	0
0	0	0	0	0	0
0	0	0	0	0	0
0	0	1	0	0	0
0	0	1	0	0	0
0	0	0	0	0	0
0	0	0	0	0	0
0	0	0	0	0	0
0	0	0	0	0	0
0	0	0	0	0	0
0	0	0	0	0	0
0	0	0	0	0	0
0	0	0	0	0	0
0	0	0	0	0	0
0	0	0	0	0	0
0	0	0	0	0	0
0	0	0	0	0	0
0	0	0	0	0	0
1	0	0	0	0	0
0	0	0	0	0	0
0	0	0	0	0	0
0	0	0	0	0	0
0	0	0	0	0	0
0	0	0	0	0	0
0	0	0	0	0	0
0	0	0	0	0	0
0	0	0	0	0	0
0	0	0	0	0	0
0	0	0	0	0	0
0	0	0	0	0	0
0	0	0	0	0	0
0	0	0	0	0	0
0	0	0	0	0	0
0	0	0	0	0	0
0	0	0	0	0	0
0	0	0	0	0	0
0	0	0	0	0	0
0	0	0	0	0	0
0	0	0	0	0	0
0	0	0	0	0	0
0	0	0	0	0	0
0	0	0	0	0	0
0	0	0	0	0	0
0	0	0	0	0	0
0	0	0	0	0	0
0	0	0	0	0	0
0	0	0	0	0	0
0	0	0	0	0	0
0	0	0	0	0	0
0	0	0	0	0	0

0	0	0	0	0	0
0	0	0	0	0	0
0	0	0	0	0	0
0	0	0	0	0	0
0	0	0	0	0	0
0	0	0	0	0	0
0	0	0	0	0	0
0	0	0	0	0	0
0	0	0	0	0	0
0	0	0	0	0	0
0	0	0	0	0	0
0	0	0	0	0	0
0	0	0	0	0	0
0	0	0	0	0	0
0	0	0	0	0	0
0	0	1	0	0	0
0	0	0	0	0	0
0	0	0	0	0	0
0	0	0	0	0	0
0	0	0	0	0	0
0	0	0	0	0	0
0	0	0	0	0	0
0	0	0	0	0	0
0	0	0	0	0	0
0	0	0	0	0	0
0	0	0	0	1	0
0	0	1	0	0	0
0	0	0	0	0	0
0	0	0	0	0	0
0	0	0	0	0	0
0	0	0	0	0	0
0	0	0	0	0	0
0	0	0	0	0	0
0	0	0	0	0	0

Verbrennung	Bisswunde	SonstigeVerletzungen	RQW	unbekannt	Neurologie	Psychiatrie
0	0	0	0	0	0	0
0	0	0	0	0	0	0
0	0	0	0	0	0	0
0	0	0	0	0	0	0
0	0	0	0	0	1	0
0	0	0	0	0	0	0
0	0	0	0	0	0	0
0	0	0	0	0	0	1
0	0	0	0	0	0	0
0	0	0	0	0	0	0
0	0	0	0	0	0	0
0	0	0	0	0	0	0
0	0	0	0	0	0	0
0	0	0	0	0	0	0
0	0	0	0	0	0	?
0	0	0	0	0	0	0
0	0	0	0	0	1	0
0	0	0	0	0	0	0
0	0	0	0	0	0	0
0	0	0	0	0	0	0
0	0	0	0	0	0	1
0	0	0	0	0	0	0
0	0	0	0	0	0	0
0	0	0	0	0	0	1
0	0	0	0	0	0	0
0	0	0	0	0	0	0
0	0	0	0	0	0	0
0	0	0	0	0	0	0
0	0	0	0	0	0	0
0	0	0	0	0	0	0
0	0	0	0	0	0	0
0	0	0	0	0	0	0
0	0	0	0	0	0	0
0	0	0	0	0	0	0
0	0	0	0	0	0	?
0	0	0	0	0	0	0
1	0	0	0	0	0	0
0	0	0	0	0	0	0
0	0	0	0	0	0	0
0	0	0	0	0	0	0
0	0	0	0	0	?	?
0	0	0	0	0	1	0
0	0	0	0	0	0	0
0	0	0	0	0	0	0
0	0	0	0	0	0	0
0	0	0	0	0	0	0
0	0	0	0	0	0	0
0	0	0	0	0	0	0
0	0	0	0	0	0	0
0	0	0	0	0	0	0
0	0	0	0	0	0	0
0	0	0	0	0	0	0
0	0	0	0	0	0	0
0	0	0	0	0	0	0
0	0	0	0	0	0	0
0	0	0	0	0	0	0
0	0	0	0	0	1	0
0	0	0	0	0	0	0
0	0	0	0	0	0	0
0	0	0	0	0	0	0
0	0	0	0	0	0	0
0	0	0	0	0	0	0
0	0	0	0	0	0	0
0	0	0	0	0	0	0
0	0	0	0	0	0	0
0	0	0	0	0	0	0
0	0	0	0	0	0	0
0	0	0	0	0	0	0
0	0	0	0	0	0	0
0	0	0	0	0	0	0
0	0	0	0	0	1	0
0	0	0	0	0	0	0
0	0	0	0	0	1	0
0	0	0	0	0	0	1
0	0	0	0	0	0	0
0	0	0	0	0	?	?

0	0	0	0	0	0	0
0	0	0	0	0	0	0
0	0	0	0	0	0	0
0	0	0	0	0	0	0
0	0	0	0	0	0	0
0	0	0	0	0	0	0
0	0	0	0	0	0	0
0	0	0	0	0	0	0
0	0	0	0	0	0	0
0	0	0	0	0	?	0
0	0	0	0	0	0	0
0	0	0	0	0	0	0
0	0	0	0	0	0	0
0	0	0	0	0	0	0
0	0	0	0	0	0	0
0	0	0	0	0	0	0
0	0	0	0	0	0	0
0	0	0	0	0	0	0
0	0	0	0	0	1	0
0	0	0	0	0	0	0
0	0	0	0	0	0	0
0	0	0	0	0	0	0
0	0	0	0	0	0	1
0	0	0	0	0	1	0
0	0	0	0	0	0	0
0	0	0	0	0	0	1
0	0	0	0	0	0	0
0	0	0	0	0	0	0
0	0	0	0	0	0	0
0	0	0	0	0	0	0
0	0	0	0	0	0	0
0	0	0	0	0	0	0
0	0	0	0	0	0	0
0	0	0	0	0	0	0
0	0	0	0	0	1	0
0	0	0	0	0	0	0
0	0	0	0	0	?	0
0	0	0	0	0	0	0
0	0	0	0	0	0	0
0	0	0	0	0	0	0
0	0	0	0	0	0	0
0	0	0	0	0	1	0
0	0	0	0	0	0	0
0	0	0	0	0	0	0
0	0	0	0	0	0	0
0	0	0	0	0	0	0
0	0	0	0	0	1	0
0	0	0	0	0	?	0
0	0	0	0	0	0	0
0	0	0	0	0	0	0
0	0	0	0	0	0	0
0	0	0	0	0	0	1
0	0	0	0	0	0	0
0	0	0	0	0	0	0
0	0	0	0	0	0	0
0	0	0	0	0	0	?
0	0	0	0	0	0	0
0	0	0	0	0	0	0
0	0	0	0	0	0	0
0	0	0	0	0	0	1
0	0	1	0	0	0	0
0	0	0	0	0	0	?
0	0	0	0	0	0	0
0	0	0	0	0	0	?
0	0	0	0	0	0	0
0	0	0	0	0	0	0
0	0	0	1	0	0	0
0	0	0	0	0	0	0
0	0	0	0	0	0	0
0	0	0	0	0	0	0
0	0	0	0	0	0	0
0	0	0	0	0	0	1
0	0	0	1	0	0	0
0	0	0	0	0	0	0
0	0	0	0	0	0	0
0	0	0	0	0	0	0
0	0	0	0	0	0	0

0	0	0	0	0	0	1
0	0	0	0	0	0	0
0	0	0	0	0	0	0
0	0	1	0	0	0	0
0	0	0	0	0	0	0
0	0	0	0	0	0	?
0	0	0	0	0	0	0
0	0	0	0	0	0	1
0	0	0	0	0	0	0
0	0	0	0	0	0	0
0	0	0	0	0	0	1
0	0	0	0	0	0	0
0	0	0	0	0	0	0
0	0	0	0	0	?	?
0	0	0	0	0	0	0
0	0	0	0	0	0	0
0	0	0	0	0	0	0
0	0	0	0	0	0	1
0	0	0	0	0	0	0
0	0	0	0	0	1	0
0	1	0	0	0	0	0
0	0	0	0	0	0	0
0	0	0	0	0	0	0
0	0	0	0	0	0	0
0	0	0	0	0	0	0
0	0	0	0	0	0	0
0	0	0	0	0	0	1
0	0	0	0	0	0	0
0	0	0	0	0	0	0
0	0	0	0	0	0	0
0	0	0	0	0	0	0
0	0	0	1	0	0	0
0	0	0	0	0	0	0
0	0	0	0	0	1	0
0	0	0	0	0	0	0
0	0	0	0	0	0	1
0	0	0	0	0	0	1
0	0	1	0	0	0	0
0	0	0	0	0	0	0
0	0	0	0	0	0	0
0	0	0	0	0	0	0
0	0	0	0	0	0	0
0	0	0	0	0	0	0
0	0	0	0	0	0	0
0	0	0	1	0	0	0
0	0	0	0	0	0	0
0	0	0	0	0	0	0
0	0	0	0	0	0	0
0	0	0	0	0	0	0
0	0	0	0	0	0	0
0	0	0	0	0	0	1
0	0	0	0	0	0	0
0	0	0	0	0	0	1
0	0	0	0	0	0	0
0	0	0	0	0	0	0
0	0	0	0	0	0	0
0	0	0	0	0	0	0
0	0	1	0	0	0	0
0	0	0	0	0	0	0
0	0	0	0	0	0	0
0	0	0	0	0	0	0
0	0	0	0	0	0	0
0	0	0	0	0	0	0
0	0	1	0	0	0	0
0	0	0	0	0	0	1
0	0	0	0	0	0	0
0	0	0	0	0	0	0
0	0	0	0	0	0	0
0	0	0	0	0	1	0
0	0	0	0	0	0	0
0	0	0	0	0	0	0
0	0	0	0	0	0	0
0	0	0	0	0	1	0
0	0	0	0	0	0	0
0	0	0	0	0	0	0
0	0	0	0	0	0	0
0	0	0	0	0	0	1

0	0	0	0	0	0	0
0	0	0	0	0	0	0
0	0	1	0	0	0	0
0	0	0	0	0	0	0
0	0	0	0	0	0	0
0	0	0	0	0	0	0
0	0	0	0	0	1	0
0	0	0	0	0	0	0
0	0	0	0	0	1	0
0	0	0	0	0	0	0
0	0	0	0	0	0	0
1	0	0	0	0	0	0
0	0	0	0	0	0	0
0	0	0	0	0	1	0
0	0	0	0	0	0	0
0	0	0	0	0	0	1
0	0	0	0	0	0	0
0	0	0	0	0	?	?
0	0	0	0	0	1	0
0	0	0	0	0	0	0
0	0	0	0	0	0	0
0	0	0	0	0	0	0
0	0	0	0	0	0	0
0	0	0	0	0	0	0
0	0	0	0	0	0	?
0	0	0	0	0	0	0
0	0	0	0	0	0	0
0	0	0	0	0	0	0
0	0	0	0	0	0	0
0	0	0	0	0	0	0
0	0	0	0	0	1	0
0	0	0	0	0	0	0
0	0	0	0	0	0	0
0	0	0	0	0	0	0
0	0	0	0	0	0	0
0	0	0	0	0	0	0
0	0	0	0	0	0	0
0	0	0	0	0	0	0
0	0	0	0	0	0	0
0	0	0	0	0	0	0
0	0	0	0	0	0	0
0	0	0	0	0	0	0
0	0	0	0	0	0	0
0	1	0	0	0	0	0
0	0	0	0	0	0	0
0	0	0	0	0	0	0
0	0	0	0	0	1	0
0	0	0	0	1	0	0
0	0	0	0	0	0	0
0	0	0	0	0	0	0
0	0	0	0	0	0	0
0	0	0	0	0	?	?
0	0	0	0	0	0	0
0	0	0	0	0	0	0
0	0	0	0	0	0	0
0	0	0	0	0	0	0
0	0	0	0	0	0	0
0	0	0	0	0	0	0
0	0	0	0	0	0	0
0	0	0	0	0	0	0
0	0	0	0	0	0	0
0	0	0	0	0	0	0
0	0	0	0	0	1	0
0	0	0	0	0	0	0
0	0	0	0	0	1	0
0	0	0	0	0	1	0
0	0	0	0	0	0	0
0	0	0	0	0	0	0
0	0	0	0	0	0	0
0	0	0	0	0	0	1
0	0	0	0	0	0	0
0	0	0	0	0	0	?
0	0	0	0	0	0	0
0	0	0	0	0	0	0
0	0	0	0	0	0	0
0	0	0	0	0	0	0
0	0	0	0	0	1	0

0	0	0	0	0	0	0
0	0	0	0	0	0	0
0	0	0	0	0	0	0
0	0	0	0	0	0	0
0	0	0	0	0	0	0
0	0	0	0	0	1	0
0	0	0	0	0	1	0
0	0	0	0	0	0	0
0	0	0	0	0	0	0
0	0	0	0	0	1	0
0	0	0	0	0	1	0
0	0	0	0	0	0	1
0	0	0	0	0	0	0
0	0	0	0	0	0	0
0	0	0	0	0	0	0
0	0	0	0	0	1	0
0	0	0	0	0	0	0
0	0	0	0	0	0	0
0	0	0	0	0	0	0
0	0	0	0	0	0	0
0	0	0	0	0	1	0
0	0	0	0	0	0	0
0	0	0	0	0	0	0
0	0	0	0	0	0	0
0	0	0	0	0	0	1
0	0	0	0	0	1	0
0	0	0	0	0	0	0
0	0	0	0	0	0	0
0	0	0	0	0	0	0
0	0	0	0	0	0	0
0	0	0	0	0	0	?
0	0	0	0	0	0	0
0	0	0	0	0	0	0
0	0	0	0	0	0	0
0	0	0	0	0	0	0
0	0	0	0	0	0	0
0	0	0	0	0	0	0
0	0	0	0	0	0	0
0	0	0	0	0	0	0
0	0	0	0	0	0	0
0	0	0	0	0	0	0
0	0	0	0	0	0	0
0	0	0	0	0	0	0
0	0	0	0	0	0	0
0	0	0	0	0	0	0
0	0	0	0	0	0	0
0	0	0	0	0	0	0
0	0	0	0	0	1	0
0	0	0	0	0	0	0
0	0	0	0	0	1	0
0	0	0	0	0	0	0
0	0	0	0	0	0	0
0	0	0	0	0	0	0
0	0	0	0	0	1	0
0	0	0	0	0	0	0
0	0	0	0	0	0	0
0	0	0	0	0	0	0
1	0	0	0	0	0	0
0	0	0	0	0	0	0
0	0	0	0	0	0	0
0	0	0	0	0	0	0
0	0	0	0	0	0	0
0	0	0	0	0	0	0
0	0	0	0	0	1	0
0	0	0	0	0	0	0
0	0	0	0	0	0	0
0	0	0	0	0	0	0
0	0	0	0	0	0	0
0	0	0	0	0	0	0
0	0	0	0	0	0	0
0	0	0	0	0	0	0
0	0	0	0	0	0	0
0	0	0	0	0	0	1
0	0	0	0	0	0	0
0	0	0	0	0	0	?
0	0	0	0	0	0	0
0	0	0	0	0	0	0

0	0	0	0	0	0	0
0	0	0	0	0	0	1
0	0	0	0	0	0	0
0	0	0	0	0	0	0
0	0	0	0	0	0	0
0	0	0	0	0	0	0
0	0	0	0	0	0	0
0	0	0	0	0	0	0
0	0	0	0	0	0	0
0	0	0	0	0	0	0
0	0	0	0	0	0	0
0	0	0	0	0	0	0
0	0	0	0	0	0	0
0	0	0	0	0	0	0
0	0	0	0	0	1	0
0	0	0	0	0	0	0
0	0	0	0	0	0	0
0	0	0	0	0	0	0
0	0	0	0	0	0	0
0	0	0	0	0	?	?
0	0	0	0	0	0	0
0	0	0	0	0	0	0
0	0	0	0	0	0	0
0	0	0	0	0	0	0
0	0	0	0	0	0	0
0	0	0	0	0	0	0
0	0	0	0	0	0	0
0	0	0	0	0	1	0
0	0	0	0	0	0	0
0	0	0	0	0	0	0
0	0	0	0	0	0	0
0	0	0	0	0	0	0
0	0	0	0	0	0	0
0	0	0	0	0	0	0
0	0	0	0	0	0	0
0	0	0	0	0	0	0
0	0	0	0	0	0	0
0	0	0	0	0	0	0
0	1	0	0	0	0	0
0	0	0	0	0	?	0
0	0	0	0	0	0	0
0	0	0	0	0	0	0
0	0	0	0	0	0	0
0	0	0	0	0	0	1
0	0	0	0	0	0	0
0	0	0	0	0	0	0
0	0	0	0	0	0	0
0	0	0	0	0	0	0
0	0	0	0	0	0	0
0	0	0	0	0	0	0
0	0	0	0	0	0	0
0	0	0	0	0	0	0
0	0	0	0	0	0	0
0	0	0	0	0	0	0
0	0	0	0	0	0	0
0	0	0	0	0	?	?
0	0	0	0	0	0	0
0	0	0	0	0	0	1
0	0	0	0	0	0	0
0	0	0	0	0	0	0
0	0	0	0	0	1	0
0	0	0	0	0	0	0
0	0	0	0	0	0	0
0	0	0	0	0	0	0
0	0	0	0	0	0	0
0	0	0	0	0	0	0
0	0	0	0	0	1	0
0	0	0	0	0	0	0
0	0	0	0	0	0	0
0	0	0	0	0	0	0
0	0	0	0	0	0	0
0	0	0	0	0	0	0
0	0	0	0	0	0	0
0	0	0	0	0	0	0
0	0	0	0	0	0	0
0	0	0	0	0	0	0
0	0	0	0	0	0	0

0	0	0	0	0	0	0
0	0	0	0	0	0	0
0	0	0	0	0	0	0
0	0	0	0	0	0	0
0	0	0	0	0	1	0
0	0	0	0	0	0	0
0	0	0	0	0	0	0
0	0	0	0	0	0	0
0	0	0	0	0	0	0
0	0	0	0	0	0	0
0	0	0	0	0	0	0
0	0	0	0	0	0	0
0	0	0	0	0	0	0
0	0	0	0	0	0	0
0	0	0	0	0	0	0
0	0	0	0	0	0	0
0	0	0	0	0	0	0
1	0	0	0	0	0	0
0	0	0	0	0	0	0
0	0	0	0	0	0	0
0	0	0	0	0	0	0
0	0	0	0	0	0	0
0	0	0	0	0	0	0
0	0	0	0	0	0	0
0	0	0	0	0	0	0
0	0	0	0	0	0	1
0	0	0	0	0	0	0
0	0	0	0	0	0	0
0	0	0	0	0	0	?
0	0	0	0	0	0	0
0	0	0	0	0	0	0
0	0	0	0	0	0	0
0	0	0	0	0	0	0
0	0	0	0	0	1	0
0	0	0	0	0	0	0
0	0	0	0	0	0	0
0	0	0	0	0	0	0
0	0	0	0	0	0	0
0	0	0	0	0	0	0
0	0	0	0	0	0	0
0	0	0	0	0	0	0
0	0	0	0	0	0	1
0	0	0	0	0	0	1
0	0	0	0	0	0	1
0	0	0	0	0	0	0
0	0	0	0	0	0	0
0	0	0	0	0	0	0
0	0	0	0	0	0	0
0	0	0	0	0	0	1
0	0	0	0	0	0	0
0	0	0	0	0	0	0
0	0	0	0	0	0	0
0	0	0	0	0	0	?
0	0	0	0	0	0	0
0	0	0	0	0	0	0
0	0	0	0	0	0	0
0	0	0	0	0	0	0
0	0	0	0	0	?	?
0	0	0	0	0	0	0
0	0	0	0	0	0	0
0	0	0	0	0	0	0
0	0	0	0	0	0	0
0	0	0	0	0	0	0
0	0	0	0	0	0	0
0	0	0	0	0	0	0
0	0	0	0	0	0	1
0	0	0	0	0	0	0
0	0	0	0	0	0	0
0	0	0	0	0	0	0
0	0	0	0	0	0	0
0	0	0	0	0	0	0
0	0	0	0	0	0	0
0	0	0	0	0	0	0
0	0	0	0	0	0	0
0	0	0	0	0	0	1
0	0	0	0	0	0	0
0	0	0	0	0	0	0

0	0	0	0	0	0	0
0	0	0	0	0	0	0
0	0	0	0	0	1	0
0	0	0	0	0	0	1
0	0	0	0	0	0	0
0	0	1	0	0	0	0
0	0	0	0	0	0	0
0	0	0	0	0	0	0
0	0	0	0	0	0	0
0	0	0	0	0	0	0
0	0	0	0	0	0	0
0	0	0	0	0	1	0
0	0	0	0	0	0	0
0	0	0	0	0	0	0
0	0	0	0	0	0	0
0	0	0	0	0	0	0
0	0	0	0	0	0	0
0	0	0	0	0	0	0
0	0	0	0	0	0	0
0	0	0	0	0	0	0
0	0	0	0	0	0	0
0	0	0	0	0	0	0
0	0	0	0	0	0	0
0	0	0	0	0	0	0
0	0	0	0	0	0	0
0	0	1	0	0	0	0
0	0	0	0	0	0	0
0	0	0	0	0	0	0
0	0	0	0	0	0	0
0	0	0	0	0	0	0
0	0	0	0	0	0	0
0	0	0	0	0	1	0
0	0	0	0	0	0	0
0	0	0	0	0	0	1
0	0	0	0	0	0	0
0	0	0	0	0	0	0
0	0	0	0	0	1	0
0	0	0	0	0	0	0
0	0	0	0	0	0	0
0	0	0	0	0	0	0
0	0	0	0	0	0	0
0	0	0	0	0	0	0
0	0	0	0	0	0	0
0	0	0	0	0	0	0
0	0	0	0	0	0	0
0	0	0	0	0	0	0
0	0	0	0	0	0	0
0	0	0	0	0	0	0
0	0	0	0	0	0	0
0	0	0	0	0	0	0
0	0	0	0	0	0	0
0	0	0	0	0	0	0
0	0	0	0	0	0	0
0	0	0	0	0	0	0
0	0	1	0	0	0	0
0	0	0	0	0	0	1
0	0	0	0	0	0	0
0	0	0	0	0	0	0
0	0	0	0	0	0	0
0	0	0	0	0	0	0
0	0	0	0	0	0	0
0	0	0	0	0	0	0
0	0	0	0	0	0	0
0	0	0	0	0	0	0
0	0	0	0	0	0	0
0	0	0	0	0	0	0
0	0	0	0	0	0	0
0	0	0	0	0	0	0
0	0	0	0	0	0	0
0	0	0	0	0	0	0
0	0	0	0	0	0	1
0	0	0	0	0	1	0
0	0	0	0	0	0	0
0	0	0	0	0	0	0
0	0	0	0	0	0	0
0	0	0	0	0	0	0
0	0	0	0	0	0	0

0	0	0	0	0	0	0
0	0	0	0	0	0	0
0	0	0	0	0	0	0
0	0	1	0	0	0	0
0	0	0	0	0	0	0
0	0	0	0	0	0	0
0	0	0	0	0	0	0
0	0	0	0	0	0	0
0	0	0	0	0	0	0
0	0	0	0	0	0	0
0	0	1	0	0	0	0
0	0	0	0	0	0	0
0	0	0	0	0	1	0
0	0	0	0	0	0	0
0	0	0	0	0	0	0
0	0	0	0	0	0	0
0	0	0	0	0	0	0
0	0	0	1	0	0	0
0	0	0	0	0	0	0
0	0	1	0	0	0	0
0	0	0	0	0	0	0
0	0	0	0	0	0	0
0	0	0	0	0	0	0
0	0	0	0	0	0	0
0	0	0	0	0	0	0
0	0	0	0	0	0	0
0	0	0	0	0	0	0
0	0	0	0	0	0	0
0	0	0	0	0	0	0
0	0	0	0	0	1	0
0	0	0	0	0	0	0
0	0	0	0	0	0	0
0	0	0	0	0	?	?
0	0	0	0	0	0	0
0	0	0	0	0	0	0
0	0	0	0	0	0	0
0	0	0	0	0	0	0
0	0	0	0	0	0	0
0	0	0	0	0	0	0
0	0	0	0	0	0	0
0	0	0	0	0	0	0
0	0	0	0	0	0	0
0	0	0	0	0	0	0
0	0	0	0	0	0	0
0	0	0	0	0	0	0
0	0	0	0	0	0	?
0	0	0	0	0	0	0
0	0	0	0	0	0	0
0	0	0	0	0	0	0
0	0	0	0	0	0	0
0	0	0	0	0	0	0
0	0	0	0	0	0	1
0	0	0	0	0	0	0
0	0	0	0	0	0	1
0	0	0	0	0	0	0
0	0	0	0	0	0	0
0	0	0	0	0	0	?
0	0	0	0	0	0	0
0	0	0	0	0	1	0
0	0	0	0	0	0	0
0	0	0	0	0	0	0
0	0	0	0	0	0	0
0	0	1	0	0	0	0
0	0	0	0	0	0	0
0	0	0	0	0	0	0
0	0	0	0	0	0	0
0	0	0	0	0	0	0
0	0	0	0	0	0	0
0	0	0	0	0	0	0
0	0	0	0	0	0	0
0	0	0	0	0	0	1
0	0	0	0	0	0	0
0	0	0	0	0	0	0
0	0	0	0	0	0	0
0	0	0	0	0	0	0
0	0	0	0	0	0	1
0	0	0	0	0	0	0

0	0	0	0	0	0	1
0	0	0	0	0	0	0
0	0	0	0	0	0	0
0	0	0	0	0	0	0
0	0	0	0	0	0	0
0	0	0	0	0	0	0
0	0	0	0	0	0	0
0	0	0	0	0	0	0
0	0	0	0	0	0	0
0	0	0	0	0	0	0
0	0	0	0	0	0	1
0	0	0	0	0	0	0
0	0	0	0	0	0	0
0	0	0	0	0	0	0
0	0	0	0	0	0	0
0	0	0	0	0	0	1
0	0	0	0	0	0	1
0	0	0	0	0	0	0
0	0	0	0	0	0	0
0	0	0	0	0	0	0
0	0	0	0	0	0	0
0	0	0	0	0	1	0
0	0	0	0	0	0	1
0	0	0	0	0	0	1
0	0	0	0	0	1	0
0	0	0	0	0	0	0
0	0	0	0	0	0	0
0	0	0	0	0	0	0
0	0	0	0	0	0	0
0	0	0	0	0	0	0
0	0	0	0	0	0	0
0	0	0	0	0	0	0
0	0	0	0	0	0	0
0	0	0	0	0	0	0
0	0	0	0	0	0	0
0	0	0	0	0	0	0
0	0	0	0	0	0	0
0	0	0	0	0	0	0
0	0	0	0	0	0	0
0	0	0	0	0	0	0
0	0	0	0	0	0	0
0	0	0	0	0	0	0
0	0	0	0	0	0	0
0	0	0	0	0	0	0
0	0	0	0	0	0	0
0	0	0	0	0	0	0
0	0	0	0	0	0	0
0	0	0	0	0	0	0
0	0	0	0	0	0	0
0	0	0	0	0	1	0
0	0	0	0	0	?	0
0	0	0	0	0	0	0
0	1	0	0	0	0	0
0	0	0	0	0	0	0
0	0	0	0	0	0	0
0	0	0	0	0	0	1
0	0	0	0	0	0	1
0	0	0	0	0	0	0
0	0	0	0	0	0	1
0	0	0	0	0	0	0
0	0	0	0	0	0	0
0	0	0	0	0	0	0
0	0	0	0	0	?	0
0	0	0	0	0	0	1
0	0	0	0	0	0	0
0	0	0	0	0	1	0
0	0	0	0	0	0	0
0	0	0	0	0	0	0
0	0	0	0	0	0	0
0	0	0	0	0	0	0
0	0	0	0	0	0	0
0	0	0	0	0	0	0
0	0	0	0	0	0	0
0	0	0	0	0	1	0
0	0	0	0	0	0	0
0	0	0	0	0	0	0
0	0	0	0	0	0	0

0	0	0	0	0	0	0
0	0	0	0	0	0	0
0	0	0	0	0	1	0
0	0	0	0	0	0	0
0	0	0	0	0	0	0
0	0	0	0	0	0	0
0	0	1	0	0	0	0
0	0	0	0	0	0	?
0	0	0	0	0	0	0
0	0	0	0	0	0	0
0	0	0	0	0	1	0
0	0	0	0	0	0	0
0	0	0	0	0	0	1
0	0	0	0	0	0	0
0	0	0	0	0	1	0
0	0	0	0	0	0	0
0	0	0	0	0	0	0
0	0	0	0	0	0	0
0	0	0	0	0	0	0
0	0	0	0	0	0	0
0	0	0	0	0	0	0
0	0	0	0	0	0	0
0	0	0	0	0	0	?
0	0	0	0	0	0	0
0	0	0	0	0	0	0
0	0	0	0	0	0	0
0	0	0	0	0	0	0
0	0	0	0	0	0	0
0	0	0	0	0	0	0
0	0	0	0	0	1	0
0	0	0	0	0	0	0
0	0	0	0	0	0	0
0	0	0	0	0	0	1
0	0	0	0	0	0	0
0	0	0	0	0	0	0
0	0	0	0	0	0	0
0	0	0	0	0	0	0
0	0	0	0	0	0	0
0	0	0	0	0	0	0
0	0	0	0	0	0	0
0	0	0	0	0	0	0
0	0	0	0	0	0	0
0	0	0	0	0	0	0
0	0	0	0	0	0	0
0	0	0	0	0	0	1
0	0	0	0	0	0	0
0	0	0	0	0	0	0
0	0	0	0	0	0	0
0	0	0	0	0	0	0
0	0	0	0	0	0	0
0	0	0	0	0	0	0
0	0	0	0	0	?	?
0	0	0	0	0	0	0
0	0	0	0	0	0	0
0	0	0	0	0	0	0
0	0	0	0	0	0	0
0	0	0	0	0	0	0
0	0	0	0	0	1	0
0	0	0	0	0	0	0
0	0	0	0	0	0	0
0	0	0	0	0	1	0
0	0	0	0	0	0	0
0	0	0	0	0	0	0
0	0	0	0	0	0	0
0	0	0	0	0	0	0
0	0	0	0	0	1	0
0	0	0	0	0	0	0
0	0	0	0	0	0	0
0	0	0	0	0	0	0
0	0	0	0	0	0	0
0	0	0	0	0	0	0
0	0	0	0	0	0	1
0	0	0	0	0	0	0
0	0	0	0	0	0	0
0	0	0	0	0	0	0
0	0	0	0	0	0	0

0	0	0	0	0	0	0
0	0	0	0	0	1	0
0	0	0	0	0	1	0
0	0	0	0	0	0	0
0	0	0	0	0	0	0
0	0	0	0	0	0	0
0	0	0	0	0	0	0
0	0	0	0	0	0	0
0	0	1	0	0	0	0
0	0	0	0	0	0	0
0	0	0	0	0	0	0
0	0	0	0	0	1	0
0	0	0	0	0	0	0
0	0	0	0	0	0	0
0	0	0	0	0	0	?
0	0	0	0	0	0	0
0	0	0	0	0	0	0
0	0	0	0	0	0	0
0	0	0	0	0	0	0
0	0	0	0	0	1	0
0	0	0	0	0	0	0
0	0	0	0	0	1	0
0	0	0	0	0	0	0
0	0	0	0	0	0	1
0	0	1	0	0	0	0
0	0	0	0	0	0	0
0	0	0	0	0	0	0
0	0	0	0	0	0	0
0	0	0	0	0	1	0
0	0	1	0	0	0	0
0	0	0	0	0	?	0
0	0	0	0	0	1	0
0	0	0	0	0	0	0
0	0	0	0	0	0	0

AngststörungPanikstörung	Depression	Anpassungsstörung	Suizidgedankenäußerungenversuch
0	0	0	0
0	0	0	0
0	0	0	0
0	0	0	0
0	0	0	0
0	0	0	0
0	0	0	0
0	0	0	0
0	0	0	0
0	0	0	0
0	0	0	0
0	0	0	0
0	0	0	0
0	0	0	0
0	0	0	?
0	0	0	0
0	0	0	0
0	0	0	0
0	0	0	0
0	0	0	0
1	0	0	0
0	0	0	0
0	0	0	0
0	0	0	1
0	0	0	0
0	0	0	0
0	0	0	0
0	0	0	0
0	0	0	0
0	0	0	0
0	0	0	0
0	0	0	0
0	0	0	0
0	0	0	0
0	0	0	0
0	0	0	0
0	0	0	0
0	0	0	0
0	0	0	0
0	0	0	0
0	0	0	0
0	0	0	0
0	0	0	0
0	0	0	0
0	0	0	0
0	0	0	0
0	0	0	0
0	0	0	0
0	0	0	0
0	0	0	0
0	0	0	0
0	0	0	0
0	0	0	0
0	0	0	0
0	0	0	0
0	0	0	0
0	0	0	0
0	0	0	0
0	0	0	0
0	0	0	0
0	0	0	0
0	0	0	0
0	0	0	0
0	0	0	0
0	0	0	0
0	0	0	0
0	0	0	0
0	0	0	0
0	0	0	0
0	0	0	0
0	0	0	0
0	0	0	0
0	0	0	0
0	0	0	1
0	0	0	0
0	0	0	0

0	0	0	0
0	0	0	0
0	0	0	0
0	0	0	0
0	0	0	0
0	0	0	0
0	0	0	0
0	0	0	0
0	0	0	0
0	0	0	0
0	0	0	0
0	0	0	0
0	0	0	0
0	0	0	0
0	0	0	0
0	0	0	0
0	0	0	0
0	0	0	0
0	0	0	0
0	0	0	0
0	0	0	0
0	0	0	0
0	0	0	0
0	0	0	0
0	0	0	0
0	0	0	0
0	0	0	0
0	0	0	0
0	0	0	0
0	0	0	0
0	0	0	0
0	0	0	0
0	0	0	0
0	0	0	0
0	0	0	0
0	0	0	0
0	0	0	0
0	0	0	0
0	0	0	0
0	0	0	0
0	0	0	0
0	0	0	0
0	0	0	0
0	0	0	0
0	0	0	0
0	0	0	0
0	0	0	0
0	0	0	0
0	0	0	0
0	0	0	0
0	0	0	0
1	0	0	0
0	0	0	0
0	0	0	0
0	0	0	0
0	0	0	0
0	0	0	0
0	0	0	0
0	0	0	0
0	0	1	1
0	0	0	0
0	0	0	0
0	0	0	0
0	1	1	0
0	0	0	0
0	0	0	0
0	0	0	0
0	0	0	0
0	0	0	0
0	0	0	0
0	0	0	0
1	0	0	0
0	0	0	0
0	0	0	0
0	0	0	0
0	0	0	0
0	0	0	0

0	1	0	1
0	0	0	0
0	0	0	0
0	0	0	0
0	0	0	0
0	0	0	0
0	0	0	0
0	0	0	0
0	0	0	0
0	0	0	0
0	0	0	1
0	0	0	0
0	0	0	0
0	0	0	0
0	0	0	0
0	0	0	0
0	0	0	0
0	1	0	0
0	0	0	0
0	0	0	0
0	0	0	0
0	0	0	0
0	0	0	0
0	0	0	0
0	0	0	0
0	0	0	0
0	0	0	1
0	0	0	0
0	0	0	0
0	0	0	0
0	0	0	0
0	0	0	0
0	0	0	0
0	0	0	0
0	0	0	0
0	0	0	0
0	1	0	0
0	0	0	0
0	0	0	0
0	0	0	0
0	0	0	0
0	0	0	0
0	0	0	0
0	0	0	0
0	0	0	0
0	0	0	0
0	0	0	0
0	0	0	0
0	0	0	0
0	0	0	0
0	1	0	1
0	0	0	0
0	0	0	0
0	0	0	0
0	0	0	0
0	0	0	0
0	0	0	0
0	0	0	0
0	0	0	0
0	0	0	0
0	0	0	0
0	0	0	0
0	0	0	0
0	0	0	0
0	0	0	0
0	0	0	0
0	0	0	0
0	0	0	0
0	0	0	0
0	0	0	0
0	0	0	0
0	0	0	0
0	0	0	0
0	0	0	0
0	0	0	0
0	0	0	0
0	0	0	0

0	0	0	0
0	0	0	0
0	0	0	0
0	0	0	0
0	0	0	0
0	0	0	0
0	0	0	0
0	0	0	0
0	0	0	0
0	0	0	0
0	0	0	0
0	0	0	0
0	0	0	0
0	0	0	0
0	0	0	0
0	0	0	0
0	0	0	0
0	0	0	0
0	0	0	0
0	0	0	0
0	0	0	0
0	0	0	0
0	0	0	0
0	0	0	0
0	0	0	0
0	0	0	0
0	0	0	0
0	0	0	0
0	0	0	0
0	0	0	0
0	0	0	0
0	0	0	0
0	0	0	0
0	0	0	0
0	0	0	0
0	0	0	0
0	0	0	0
0	0	0	0
0	0	0	0
0	0	0	0
0	0	0	0
0	0	0	0
0	0	0	0
0	0	0	0
0	0	0	0
0	0	0	0
0	0	0	0
0	0	0	0
0	0	0	0
0	0	0	0
0	0	0	0
0	0	0	0
0	0	0	0
0	0	0	0
0	0	0	0
0	0	0	0
0	0	0	0
0	0	0	0
0	0	0	0
0	0	0	0
0	0	0	0
0	0	0	0
0	0	0	0
0	0	0	0
0	0	0	0
0	0	0	0
0	0	0	0
0	0	0	0
0	0	0	0
0	0	0	1
0	0	0	0
0	0	0	0
0	0	0	0
0	0	0	0
0	0	0	0
0	0	0	0
0	0	0	0

0	0	0	0
0	0	0	0
0	0	0	0
0	0	0	0
0	0	0	0
0	0	0	0
0	0	0	0
0	0	0	0
0	0	0	0
0	0	0	0
0	0	0	0
0	1	0	1
0	0	0	0
0	0	0	0
0	0	0	0
0	0	0	0
0	0	0	0
0	0	0	0
0	0	0	0
0	0	0	0
0	0	0	0
0	0	0	0
0	0	0	0
0	0	0	0
0	1	1	0
0	0	0	0
0	0	0	0
0	0	0	0
0	0	0	0
0	0	0	0
0	0	0	0
0	0	0	0
0	0	0	0
0	0	0	0
0	0	0	0
0	0	0	0
0	0	0	0
0	0	0	0
0	0	0	0
0	0	0	0
0	0	0	0
0	0	0	0
0	0	0	0
0	0	0	0
0	0	0	0
0	0	0	0
0	0	0	0
0	0	0	0
0	0	0	0
0	0	0	0
0	0	0	0
0	0	0	0
0	0	0	0
0	0	0	0
0	0	0	0
0	0	0	0
0	0	0	0
0	0	0	0
0	0	0	0
0	0	0	0
0	0	0	0
0	0	0	0
0	0	0	0
0	0	0	0
0	0	0	0
0	0	0	0
0	0	0	0
0	0	0	0
0	0	0	0
0	0	0	0
0	0	0	0
0	0	0	0
1	0	0	0
0	0	0	0
0	0	0	0
0	0	0	0
0	0	0	0

0	0	0	0
0	0	0	0
0	0	0	0
0	0	0	0
0	0	0	0
0	0	0	0
0	0	0	0
0	0	0	0
0	0	0	0
0	0	0	0
0	0	0	0
0	0	0	0
0	0	0	0
0	0	0	0
0	0	0	0
0	0	0	0
0	0	0	0
0	0	0	0
0	0	0	0
0	0	0	0
0	0	0	0
0	0	0	0
0	0	0	0
0	0	0	0
0	0	0	0
0	0	0	0
0	0	0	0
0	0	0	0
0	0	0	0
0	0	0	0
0	0	0	0
0	0	0	0
0	0	0	0
0	0	0	0
0	0	0	0
0	0	0	0
0	0	0	0
0	0	0	0
0	0	0	0
0	0	0	0
0	0	0	0
0	0	0	0
0	0	0	0
0	0	0	0
0	0	0	0
0	0	0	0
0	0	0	0
0	0	0	0
0	0	0	0
0	0	0	0
0	0	0	0
0	0	0	0
0	0	0	0
0	0	0	0
0	0	0	0
0	0	0	0
0	0	0	0
0	1	0	1
0	0	0	0
0	0	0	0
0	0	0	0
0	0	0	0
0	0	0	0
0	0	0	0
0	0	0	0
0	0	0	0
0	0	0	0
0	0	0	0
0	0	0	0
0	0	0	0
0	0	0	0
0	0	0	0
0	0	0	0
0	0	0	0
0	0	0	0
0	0	0	0
0	0	0	0

0	0	0	0
0	0	0	0
0	0	0	0
0	0	0	0
0	0	0	0
0	0	0	0
0	0	0	0
0	0	0	0
0	0	0	0
0	0	0	0
0	0	0	0
0	0	0	0
0	0	0	0
0	0	0	0
0	0	0	0
0	0	0	0
0	0	0	0
0	0	0	0
0	0	0	0
0	0	0	0
0	0	0	0
0	0	0	0
0	0	0	0
0	0	0	0
0	0	0	0
0	1	1	0
0	0	0	0
0	0	0	0
0	0	0	?
0	0	0	0
0	0	0	0
0	0	0	0
0	0	0	0
0	0	0	0
0	0	0	0
0	0	0	0
0	0	0	0
0	0	0	0
0	0	0	0
0	0	0	0
0	0	0	0
0	0	0	0
0	0	0	0
0	1	0	1
0	0	0	0
0	0	0	0
0	0	0	0
0	0	0	0
0	1	0	1
0	0	0	0
0	0	0	0
0	0	0	0
0	0	1	1
0	0	0	0
0	0	0	0
0	0	0	0
0	0	0	0
0	0	0	0
0	0	0	0
0	0	0	0
0	0	0	0
0	0	0	0
0	0	0	0
0	0	0	0
0	0	0	0
0	0	0	0
0	0	0	0
0	0	0	0
0	0	0	0
0	0	0	0
0	0	0	0
0	0	0	0
0	0	0	0
0	0	0	0
0	1	0	0
0	0	0	0
0	0	0	0

0	0	0	0
0	0	0	0
0	0	0	0
0	0	0	0
0	0	0	0
0	0	0	0
0	0	0	0
0	0	0	0
0	0	0	0
0	0	0	0
0	0	0	0
0	0	0	0
0	0	0	0
0	0	0	0
0	0	0	0
0	0	0	0
0	0	0	0
0	0	0	0
0	0	0	0
0	0	0	0
0	0	0	0
0	0	0	0
0	0	0	0
0	0	0	0
0	0	0	0
0	0	0	0
0	0	0	0
0	0	0	0
0	0	0	0
0	0	0	0
0	0	0	0
0	0	0	0
0	0	0	0
0	0	0	1
0	0	0	0
0	0	0	0
0	0	0	0
0	0	0	0
0	0	0	0
0	0	0	0
0	0	0	0
0	0	0	0
0	0	0	0
0	0	0	0
0	0	0	0
0	0	0	0
0	0	0	0
0	0	0	0
0	0	0	0
0	0	0	0
0	0	0	0
0	0	0	0
0	0	0	0
0	0	0	0
0	0	0	0
0	0	0	0
0	0	0	0
0	0	0	0
0	0	0	0
0	0	0	0
0	0	0	0
0	0	0	0
0	0	0	0
0	0	0	0
0	0	0	0
0	0	0	0
0	0	0	0
0	0	0	0
0	0	0	0
0	0	0	0
0	1	1	0
0	0	0	0
0	0	0	0
0	0	0	0
0	0	0	0
0	0	0	0
0	0	0	0

0	0	0	0
0	0	0	0
0	0	0	0
0	0	0	0
0	0	0	0
0	0	0	0
0	0	0	0
0	0	0	0
0	0	0	0
0	0	0	0
0	0	0	0
0	0	0	0
0	0	0	0
0	0	0	0
0	0	0	0
0	0	0	0
0	0	0	0
0	0	0	0
0	0	0	0
0	0	0	0
0	0	0	0
0	0	0	0
0	0	0	0
0	0	0	0
0	0	0	0
0	0	0	0
0	0	0	0
0	0	0	0
0	0	0	0
0	0	0	0
0	0	0	0
0	0	0	0
0	0	0	0
0	0	0	0
0	0	0	0
0	0	0	0
0	0	0	0
0	0	0	0
0	0	0	0
0	0	0	0
0	0	0	0
0	0	0	0
0	0	0	0
0	0	0	0
0	0	0	0
0	0	0	0
0	0	0	0
0	0	0	0
0	0	0	0
0	0	0	0
0	0	0	0
0	0	0	0
0	0	0	0
0	0	0	0
0	0	0	0
0	0	0	0
0	0	0	0
0	0	0	0
0	0	0	0
0	0	0	0
0	0	0	0
0	0	0	0
0	0	0	0
0	0	0	0
0	0	0	0
0	0	0	0
0	0	0	0
0	0	0	0
0	0	0	0
0	0	0	0
0	0	0	0
0	0	0	0
0	0	0	0
0	0	0	0
0	0	0	0
0	1	0	0
0	0	0	0

0	1	1	0
0	0	0	0
0	0	0	0
0	0	0	0
0	0	0	0
0	0	0	0
0	0	0	0
0	0	0	0
0	0	0	0
0	0	0	0
0	0	0	0
0	0	0	0
0	0	0	0
0	0	0	0
0	0	0	0
0	1	0	0
0	0	0	0
0	0	0	0
0	0	0	0
0	0	0	0
0	0	0	0
0	0	0	0
0	1	0	0
0	0	0	0
0	0	0	0
0	0	0	0
0	0	0	0
0	0	0	0
0	0	0	0
0	0	0	0
0	0	0	0
0	0	0	0
0	0	0	0
0	0	0	0
0	0	0	0
0	0	0	0
0	0	0	0
0	0	0	0
0	0	0	0
0	0	0	0
0	0	0	0
0	0	0	0
0	0	0	0
0	0	0	0
0	0	0	0
0	0	0	0
0	0	0	0
0	0	0	0
0	0	0	0
0	0	0	0
0	0	0	0
0	0	0	0
0	0	0	0
0	0	0	0
0	0	0	0
0	1	0	1
1	1	0	0
0	0	0	0
0	1	1	0
0	0	0	0
0	0	0	0
0	0	0	0
0	0	0	0
0	0	0	1
0	0	0	0
0	0	0	0
0	0	0	0
0	0	0	0
0	0	0	0
0	0	0	0
0	0	0	0
0	0	0	0
0	0	0	0
0	0	0	0
0	0	0	0
0	0	0	0
0	0	0	0

0	0	0	0
0	0	0	0
0	0	0	0
0	0	0	0
0	0	0	0
0	0	0	0
0	0	0	0
0	0	0	?
0	0	0	0
0	0	0	0
0	0	0	0
0	0	0	0
1	0	0	0
0	0	0	0
0	0	0	0
0	0	0	0
0	0	0	0
0	0	0	0
0	0	0	0
0	0	0	0
0	0	0	0
0	0	0	0
0	0	0	0
0	0	0	0
0	0	0	0
0	0	0	0
0	0	0	1
0	0	0	0
0	0	0	0
0	0	0	0
0	0	0	0
0	0	0	0
0	0	0	1
0	0	0	0
0	0	0	0
0	0	0	0
0	0	0	0
0	0	0	0
0	0	0	0
0	0	0	0
0	0	0	0
0	0	0	0
0	0	0	0
0	0	0	0
0	0	1	0
0	0	0	0
0	0	0	0
0	0	0	0
0	0	0	0
0	0	0	0
0	0	0	0
0	0	0	0
0	0	0	0
0	0	0	0
0	0	0	0
0	0	0	0
0	0	0	0
0	0	0	0
0	0	0	0
0	0	0	0
0	0	0	0
0	0	0	0
0	0	0	0
0	0	0	0
0	0	0	0
0	0	0	0
0	0	0	0
0	0	0	0
0	0	0	0
0	0	0	0
0	0	0	0
0	0	0	0
0	1	0	1
0	0	0	0
0	0	0	0
0	0	0	0
0	0	0	0

0	0	0	0
0	0	0	0
0	0	0	0
0	0	0	0
0	0	0	0
0	0	0	0
0	0	0	0
0	0	0	0
0	0	0	0
0	0	0	0
0	0	0	0
0	0	0	0
0	0	0	0
0	0	0	0
0	0	0	0
0	0	0	0
0	0	0	0
0	0	0	0
0	0	0	0
0	0	0	0
0	0	0	0
0	0	0	0
0	0	0	0
0	1	0	1
0	0	0	0
0	0	0	0
0	0	0	0
0	0	0	0
0	0	0	0
0	0	0	0
0	0	0	0
0	0	0	0
0	0	0	0
0	0	0	0

Selbstverletzung_A	PsychosozialeBelastung	PTBS	Psychose	Persönlichkeitstörung	Entzug
0	0	0	0	0	0
0	0	0	0	0	0
0	0	0	0	0	0
0	0	0	0	0	0
0	0	0	0	0	0
0	0	0	0	0	0
0	0	0	0	0	0
0	1	0	0	0	0
0	0	0	0	0	0
0	0	0	0	0	0
0	0	0	0	0	0
0	0	0	0	0	0
0	0	0	0	0	0
0	0	0	0	0	0
0	0	0	0	0	0
0	0	0	0	0	0
0	0	0	0	0	0
0	0	0	0	0	0
0	0	0	0	0	0
0	0	0	0	0	0
0	0	0	1	0	0
0	0	0	0	0	0
0	0	0	0	0	0
0	1	0	0	0	0
0	0	0	0	0	0
0	0	0	0	0	0
0	0	0	0	0	0
0	0	0	0	0	0
0	0	0	0	0	0
0	0	0	0	0	0
0	0	0	0	0	0
0	0	0	0	0	0
0	0	0	0	0	0
0	0	0	0	0	0
0	0	0	0	0	0
0	0	0	0	0	0
0	0	0	0	0	0
0	0	0	0	0	0
0	0	0	0	0	0
0	0	0	0	0	0
0	0	0	0	0	0
0	0	0	0	0	0
0	0	0	0	0	0
0	0	0	0	0	0
0	0	0	0	0	0
0	0	0	0	0	0
0	0	0	0	0	0
0	0	0	0	0	0
0	0	0	0	0	0
0	0	0	0	0	0
0	0	0	0	0	0
0	0	0	0	0	0
0	0	0	0	0	0
0	0	0	0	0	0
0	0	0	0	0	0
0	0	0	0	0	0
0	0	0	0	0	0
0	0	0	0	0	0
0	0	0	0	0	0
0	0	0	0	0	0
0	0	0	0	0	0
0	0	0	0	0	0
0	0	0	0	0	0
0	0	0	0	0	0
0	0	0	0	0	0
0	0	0	0	0	0
0	0	0	0	0	0
0	0	0	0	0	0
0	0	0	0	0	0
0	0	0	0	0	0
0	0	0	0	0	0
0	0	0	0	0	0
0	0	0	0	0	0
0	1	0	0	0	0
0	0	0	0	0	0
0	0	0	0	0	0

0	0	0	0	0	0
0	0	0	0	0	0
0	0	0	0	0	0
0	0	0	0	0	0
0	0	0	0	0	0
0	0	0	0	0	0
0	0	0	0	0	0
0	0	0	0	0	0
0	0	0	0	0	0
0	0	0	0	0	0
0	0	0	0	0	0
0	0	0	0	0	0
0	0	0	0	0	0
0	0	0	0	0	0
0	0	0	0	0	0
0	0	0	0	0	0
0	0	0	0	0	0
0	0	0	0	0	0
0	0	0	0	0	0
0	0	0	0	0	0
0	0	0	0	0	0
0	0	0	0	0	0
0	0	0	0	0	0
0	0	0	0	0	0
0	0	0	0	0	0
0	1	0	0	0	0
0	0	0	0	0	0
0	0	0	0	0	0
0	0	0	0	0	0
0	0	0	0	0	0
0	0	0	0	0	0
0	0	0	0	0	0
0	0	0	0	0	0
0	0	0	0	0	0
0	0	0	0	0	0
0	0	0	0	0	0
0	0	0	0	0	0
0	0	0	0	0	0
0	0	0	0	0	0
0	0	0	0	0	0
0	0	0	0	0	0
0	0	0	0	0	0
0	0	0	0	0	0
0	0	0	0	0	0
0	0	0	0	0	0
0	0	0	0	0	0
0	0	0	0	0	0
0	0	0	0	0	0
0	0	0	0	0	0
0	0	0	0	0	0
0	0	0	0	0	0
0	0	0	0	0	0
0	0	0	0	0	0
0	0	0	0	0	0
0	0	0	0	0	0
0	0	0	0	0	0
0	0	0	0	0	0
0	0	0	0	0	0
0	0	0	0	0	0
0	0	0	0	0	0
0	0	0	0	0	0
0	0	0	0	0	0
0	0	0	0	0	0
0	0	0	0	0	0
0	0	0	0	0	0
0	0	0	0	0	0
0	0	0	0	0	0
0	0	0	0	0	0
0	0	0	0	0	0
0	0	0	0	0	0
0	0	0	0	0	0
0	1	0	0	0	0
0	0	0	0	0	0
0	0	0	0	0	0
0	0	0	0	0	0
0	0	0	0	0	0
0	0	0	0	0	0

0	0	0	0	0	0
0	0	0	0	0	0
0	0	0	0	0	0
0	0	0	0	0	0
0	0	0	0	0	0
0	0	0	0	0	0
0	0	0	0	0	0
0	0	0	1	0	0
0	0	0	0	0	0
0	0	0	0	0	0
0	1	0	1	0	0
0	0	0	0	0	0
0	0	0	0	0	0
0	0	0	0	0	0
0	0	0	0	0	0
0	0	0	0	0	0
0	0	0	0	0	0
0	0	0	0	0	0
0	0	0	0	0	0
0	0	0	0	0	0
0	0	0	0	0	0
0	0	0	0	0	0
0	0	0	0	0	0
0	0	0	0	0	0
0	0	0	0	0	0
0	0	0	0	0	0
0	1	0	1	0	0
0	0	0	0	0	0
0	0	0	0	0	0
0	0	0	0	0	0
0	0	0	0	0	0
0	0	0	0	0	0
0	0	0	0	0	0
0	0	0	0	0	0
0	0	0	0	0	0
0	0	1	0	0	0
0	1	1	1	0	0
0	0	0	0	0	0
0	0	0	0	0	0
0	0	0	0	0	0
0	0	0	0	0	0
0	0	0	0	0	0
0	0	0	0	0	0
0	0	0	0	0	0
0	0	0	0	0	0
0	0	0	0	0	0
0	0	0	0	0	0
0	0	0	0	0	0
0	0	0	0	0	0
0	0	0	0	0	0
0	0	0	1	0	0
0	0	0	0	0	0
0	0	0	1	0	0
0	0	0	0	0	0
0	0	0	0	0	0
0	0	0	0	0	0
0	0	0	0	0	0
0	0	0	0	0	0
0	0	0	0	0	0
0	0	0	0	0	0
0	0	0	0	0	0
0	0	0	0	0	0
0	0	0	0	0	0
0	0	0	0	0	0
0	0	0	1	0	0
0	0	0	0	0	0
0	0	0	0	0	0
0	0	0	0	0	0
0	0	0	0	0	0
0	0	0	0	0	0
0	0	0	0	0	0
0	0	0	0	0	0
0	0	0	0	0	0
0	0	0	0	0	0
0	0	0	0	0	0
0	0	0	0	0	0
0	1	0	0	0	0

0	0	0	0	0	0
0	0	0	0	0	0
0	0	0	0	0	0
0	0	0	0	0	0
0	0	0	0	0	0
0	0	0	0	0	0
0	0	0	0	0	0
0	0	0	0	0	0
0	0	0	0	0	0
0	0	0	0	0	0
0	0	0	0	0	0
0	0	0	0	0	0
0	0	0	0	0	0
0	0	0	0	0	0
0	0	0	0	0	0
0	0	0	0	1	0
0	0	0	0	0	0
0	0	0	0	0	0
0	0	0	0	0	0
0	0	0	0	0	0
0	0	0	0	0	0
0	0	0	0	0	0
0	0	0	0	0	0
0	0	0	0	0	0
0	0	0	0	0	0
0	0	0	0	0	0
0	0	0	0	0	0
0	0	0	0	0	0
0	0	0	0	0	0
0	0	0	0	0	0
0	0	0	0	0	0
0	0	0	0	0	0
0	0	0	0	0	0
0	0	0	0	0	0
0	0	0	0	0	0
0	0	0	0	0	0
0	0	0	0	0	0
0	0	0	0	0	0
0	0	0	0	0	0
0	0	0	0	0	0
0	0	0	0	0	0
0	0	0	0	0	0
0	0	0	0	0	0
0	0	0	0	0	0
0	0	0	0	0	0
0	0	0	0	0	0
0	0	0	0	0	0
0	0	0	0	0	0
0	0	0	0	0	0
0	0	0	0	0	0
0	0	0	0	0	0
0	0	0	0	0	0
0	0	0	0	0	0
0	0	0	0	0	0
0	0	0	0	0	0
0	0	0	0	0	0
0	0	0	0	0	0
0	0	0	0	0	0
0	0	0	0	0	0
0	0	0	0	0	0
0	0	0	0	0	0
0	0	0	0	0	0
0	0	0	0	0	0
0	0	0	0	0	0
0	0	0	0	0	0
0	0	0	0	0	0
0	0	0	0	0	0
0	0	0	0	0	0
0	0	0	0	0	0
0	1	?	0	0	0
0	0	0	0	0	0
0	0	0	0	0	0
0	0	0	0	0	0
0	0	0	0	0	0
0	0	0	0	0	0
0	0	0	0	0	0
0	0	0	0	0	0

0	0	0	0	0	0
0	0	0	0	0	0
0	0	0	0	0	0
0	0	0	0	0	0
0	0	0	0	0	0
0	0	0	0	0	0
0	0	0	0	0	0
0	0	0	0	0	0
0	0	0	0	0	0
0	0	0	0	0	0
0	0	0	0	0	0
0	1	0	0	0	0
0	0	0	0	0	0
0	0	0	0	0	0
0	0	0	0	0	0
0	0	0	0	0	0
0	0	0	0	0	0
0	0	0	0	0	0
0	0	0	0	0	0
0	0	0	0	0	0
0	0	0	0	0	0
0	0	0	0	0	0
0	0	0	0	0	0
0	0	0	0	0	0
0	0	0	0	0	0
0	0	0	0	0	0
0	0	0	0	0	0
0	0	0	0	0	0
0	0	0	0	0	0
0	0	0	0	0	0
0	0	0	0	0	0
0	0	0	0	0	0
0	0	0	0	0	0
0	0	0	0	0	0
0	0	0	0	0	0
0	0	0	0	0	0
0	0	0	0	0	0
0	0	0	0	0	0
0	0	0	0	0	0
0	0	0	0	0	0
0	0	0	0	0	0
0	0	0	0	0	0
0	0	0	0	0	0
0	0	0	0	0	0
0	0	0	0	0	0
0	0	0	0	0	0
0	0	0	0	0	0
0	0	0	0	0	0
0	0	0	0	0	0
0	0	0	0	0	0
0	0	0	0	0	0
0	0	0	0	0	0
0	0	0	0	0	0
0	0	0	0	0	0
0	0	0	0	0	0
0	0	0	0	0	0
0	0	0	0	0	0
0	0	0	0	0	0
0	0	0	0	0	0
0	0	0	0	0	0
0	0	0	0	0	0
0	0	0	0	0	0
0	0	0	0	0	0
0	0	0	0	0	0
0	0	0	0	0	0
0	0	0	0	0	0
0	0	0	0	0	0
0	0	0	0	0	0
0	0	0	0	0	0
0	0	0	0	0	0
0	0	0	0	0	0
0	0	0	0	0	0
0	1	0	0	0	0
0	0	0	0	0	0
0	0	0	0	0	0
0	0	0	0	0	0
0	0	0	0	0	0

0	0	0	0	0	0
0	0	0	1	0	0
0	0	0	0	0	0
0	0	0	0	0	0
0	0	0	0	0	0
0	0	0	0	0	0
0	0	0	0	0	0
0	0	0	0	0	0
0	0	0	0	0	0
0	0	0	0	0	0
0	0	0	0	0	0
0	0	0	0	0	0
0	0	0	0	0	0
0	0	0	0	0	0
0	0	0	0	0	0
0	0	0	0	0	0
0	0	0	0	0	0
0	0	0	0	0	0
0	0	0	0	0	0
0	0	0	0	0	0
0	0	0	0	0	0
0	0	0	0	0	0
0	0	0	0	0	0
0	0	0	0	0	0
0	0	0	0	0	0
0	0	0	0	0	0
0	0	0	0	0	0
0	0	0	0	0	0
0	0	0	0	0	0
0	0	0	0	0	0
0	0	0	0	0	0
0	0	0	0	0	0
0	0	0	0	0	0
0	0	0	0	0	0
0	0	0	0	0	0
0	0	0	0	0	0
0	0	0	0	0	0
0	0	0	0	0	0
0	0	0	0	0	0
0	0	0	0	0	0
0	0	0	0	0	0
0	0	0	0	0	0
0	0	0	0	0	0
1	0	0	1	0	0
0	0	0	0	0	0
0	0	0	0	0	0
0	0	0	0	0	0
0	0	0	0	0	0
0	0	0	0	0	0
0	0	0	0	0	0
0	0	0	0	0	0
0	0	0	0	0	0
0	0	0	0	0	0
0	0	0	0	0	0
0	0	0	0	0	0
0	0	0	0	0	0
0	0	0	0	0	0
0	0	0	0	0	0
0	0	0	0	0	0
0	0	0	0	0	0
0	0	0	0	0	0
0	0	0	0	0	0
0	0	0	0	0	0
0	0	0	0	0	0
0	0	0	0	0	0
0	0	0	0	0	0
0	0	0	0	0	0
0	0	0	0	0	0
0	0	0	0	0	0
0	0	0	0	0	0
0	0	0	0	0	0
0	0	0	0	0	0
0	0	0	0	0	0
0	0	0	0	0	0
0	0	0	0	0	0
0	0	0	0	0	0
0	0	0	0	0	0

0	0	0	0	0	0
0	0	0	0	0	0
0	0	0	0	0	0
0	0	0	0	0	0
0	0	0	0	0	0
0	0	0	0	0	0
0	0	0	0	0	0
0	0	0	0	0	0
0	0	0	0	0	0
0	0	0	0	0	0
0	0	0	0	0	0
0	0	0	0	0	0
0	0	0	0	0	0
0	0	0	0	0	0
0	0	0	0	0	0
0	0	0	0	0	0
0	0	0	0	0	0
0	0	0	0	0	0
0	0	0	0	0	0
0	0	0	0	0	0
0	0	0	0	0	0
0	0	0	0	0	0
0	0	0	0	0	0
0	0	0	0	0	0
0	0	0	0	0	0
0	0	0	0	0	0
0	0	0	0	0	0
0	0	0	0	0	0
0	0	0	0	0	0
0	0	0	0	0	0
0	0	0	0	0	0
0	0	0	0	0	0
0	0	0	0	0	0
0	0	0	0	0	0
0	0	0	0	0	0
0	0	0	0	0	0
0	0	0	0	0	0
0	0	0	0	0	0
0	0	0	0	0	0
0	0	0	0	0	0
0	0	0	0	0	0
0	1	0	0	1	0
0	1	0	0	0	0
0	1	0	0	0	0
0	0	0	0	0	0
0	0	0	0	0	0
0	0	0	0	0	0
0	0	0	0	0	0
0	1	0	0	0	0
0	0	0	0	0	0
0	0	0	0	0	0
0	0	0	0	0	0
0	0	0	0	0	0
0	0	0	0	0	0
0	0	0	0	0	0
0	0	0	0	0	0
0	0	0	0	0	0
0	?	?	0	0	0
0	0	0	0	0	0
0	0	0	0	0	0
0	0	0	0	0	0
0	0	0	0	0	0
0	0	0	0	0	0
0	0	0	0	0	0
0	0	0	0	0	0
1	0	0	1	0	0
0	0	0	0	0	0
0	0	0	0	0	0
0	0	0	0	0	0
0	0	0	0	0	0
0	0	0	0	0	0
0	0	0	0	0	0
0	0	0	0	0	0
0	0	0	0	0	0
0	0	0	0	0	0
0	0	0	0	0	0
0	0	0	0	0	0

0	0	0	0	0	0
0	0	0	0	0	0
0	0	0	0	0	0
0	1	0	0	0	0
0	0	0	0	0	0
0	0	0	0	0	0
0	0	0	0	0	0
0	0	0	0	0	0
0	0	0	0	0	0
0	0	0	0	0	0
0	0	0	0	0	0
0	0	0	0	0	0
0	0	0	0	0	0
0	0	0	0	0	0
0	0	0	0	0	0
0	0	0	0	0	0
0	0	0	0	0	0
0	0	0	0	0	0
0	0	0	0	0	0
0	0	0	0	0	0
0	0	0	0	0	0
0	0	0	0	0	0
0	0	0	0	0	0
0	0	0	0	0	0
0	0	0	0	0	0
0	0	0	0	0	0
0	0	0	0	0	0
0	0	0	0	0	0
0	0	0	0	0	0
0	0	0	0	0	0
0	0	0	0	0	0
0	0	0	0	0	0
0	0	0	0	0	0
0	0	0	0	1	0
0	0	0	0	0	0
0	0	0	0	0	0
0	0	0	0	0	0
0	0	0	0	0	0
0	0	0	0	0	0
0	0	0	0	0	0
0	0	0	0	0	0
0	0	0	0	0	0
0	0	0	0	0	0
0	0	0	0	0	0
0	0	0	0	0	0
0	0	0	0	0	0
0	0	0	0	0	0
0	0	0	0	0	0
0	0	0	0	0	0
0	0	0	0	0	0
0	0	0	0	0	0
0	0	0	0	0	0
0	0	0	0	0	0
0	0	0	0	0	0
0	0	0	0	0	0
1	0	0	1	0	0
0	0	0	0	0	0
0	0	0	0	0	0
0	0	0	0	0	0
0	0	0	0	0	0
0	0	0	0	0	0
0	0	0	0	0	0
0	0	0	0	0	0
0	0	0	0	0	0
0	0	0	0	0	0
0	0	0	0	0	0
0	0	0	0	0	0
0	0	0	0	0	0
0	0	0	0	0	0
0	0	0	0	0	0
0	0	0	0	0	0
0	0	0	0	0	0
0	0	0	0	0	0
0	0	0	0	0	0
0	0	0	0	0	0
0	0	0	0	0	0
0	0	0	0	0	0

0	0	0	0	0	0
0	0	0	0	0	0
0	0	0	0	0	0
0	0	0	0	0	0
0	0	0	0	0	0
0	0	0	0	0	0
0	0	0	0	0	0
0	0	0	0	0	0
0	0	0	0	0	0
0	0	0	0	0	0
0	0	0	0	0	0
0	0	0	0	0	0
0	0	0	0	0	0
0	0	0	0	0	0
0	0	0	0	0	0
0	0	0	0	0	0
0	0	0	0	0	0
0	0	0	0	0	0
0	0	0	0	0	0
0	0	0	0	0	0
0	0	0	0	0	0
0	0	0	0	0	0
0	0	0	0	0	0
0	0	0	0	0	0
0	0	0	0	0	0
0	0	0	0	0	0
0	0	0	0	0	0
0	0	0	0	0	0
0	0	0	0	0	0
0	0	0	0	0	0
0	0	0	0	0	0
0	0	0	0	0	0
0	0	0	0	0	0
0	0	0	0	0	0
0	0	0	0	0	0
0	0	0	0	0	0
0	0	0	0	0	0
0	0	0	0	0	0
0	0	0	0	0	0
0	0	0	0	0	0
0	0	0	0	0	0
0	0	0	0	0	0
0	0	0	0	0	0
0	0	0	0	0	0
0	0	0	0	0	0
0	0	0	0	0	0
0	0	0	0	0	0
0	0	0	0	0	0
0	0	0	0	0	0
0	0	0	0	0	0
0	0	0	0	0	0
0	1	0	0	0	0
0	0	0	0	0	0
0	1	0	0	0	0
0	0	0	0	0	0
0	0	0	0	0	0
0	0	0	0	0	0
0	0	0	0	0	0
0	0	0	0	0	0
0	0	0	0	0	0
0	0	0	0	0	0
0	0	0	0	0	0
0	0	0	0	0	0
0	0	0	0	0	0
0	0	0	0	0	0
0	0	0	0	0	0
0	0	0	0	0	0
0	0	0	0	0	0
0	0	0	0	0	0
0	0	0	0	0	0
0	1	0	0	0	0
0	0	0	0	0	0
0	0	0	0	0	0
0	0	0	0	0	0
0	0	0	0	0	0
0	0	0	0	0	0
0	0	0	0	0	0

0	0	0	0	0	0
0	0	0	0	0	0
0	0	0	0	0	0
0	0	0	0	0	0
0	0	0	0	0	0
0	0	0	0	0	0
0	0	0	0	0	0
0	0	0	0	0	0
0	0	0	0	0	0
0	0	0	0	0	0
0	0	0	0	0	1
0	0	0	0	0	0
0	0	0	0	0	0
0	0	0	0	0	0
0	0	0	0	0	0
0	0	1	0	0	0
0	0	0	1	0	0
0	0	0	0	0	0
0	0	0	0	0	0
0	0	0	0	0	0
0	0	0	0	0	0
0	0	0	0	0	0
0	1	0	0	0	0
0	0	0	0	0	0
0	0	0	0	0	0
0	0	0	0	0	0
0	0	0	0	0	0
0	0	0	0	0	0
0	0	0	0	0	0
0	0	0	0	0	0
0	0	0	0	0	0
0	0	0	0	0	0
0	0	0	0	0	0
0	0	0	0	0	0
0	0	0	0	0	0
0	0	0	0	0	0
0	0	0	0	0	0
0	0	0	0	0	0
0	0	0	0	0	0
0	0	0	0	0	0
0	0	0	0	0	0
0	0	0	0	0	0
0	0	0	0	0	0
0	0	0	0	0	0
0	0	0	0	0	0
0	0	0	0	0	0
0	0	0	0	0	0
0	0	0	0	0	0
0	0	0	0	0	0
0	0	0	0	0	0
0	0	0	0	0	0
0	0	0	0	0	0
0	0	0	0	0	0
0	0	0	0	0	0
0	0	0	0	0	0
0	1	0	0	0	0
0	0	0	0	0	0
0	0	0	0	0	0
0	0	0	0	0	0
0	0	0	0	0	0
0	0	0	0	0	0
0	0	0	0	0	0
0	0	0	0	0	0
0	1	0	0	0	0
0	0	0	0	0	0
0	0	0	0	0	0
0	0	0	0	0	0
0	0	0	0	0	0
0	0	0	0	0	0
0	0	0	0	0	0
0	0	0	0	0	0
0	0	0	0	0	0
0	0	0	0	0	0
0	0	0	0	0	0
0	0	0	0	0	0
0	0	0	0	0	0
0	0	0	0	0	0

0	0	0	0	0	0
0	0	0	0	0	0
0	0	0	0	0	0
0	0	0	0	0	0
0	0	0	0	0	0
0	0	0	0	0	0
0	0	0	0	0	0
0	0	0	0	0	0
0	0	0	0	0	0
0	0	0	0	0	0
0	0	0	0	0	0
0	0	0	0	0	0
0	0	0	0	0	0
0	0	0	0	0	0
0	0	0	0	0	0
0	0	0	0	0	0
0	0	0	0	0	0
0	0	0	0	0	0
0	0	0	0	0	0
0	0	0	0	0	0
0	0	0	0	0	0
0	0	0	0	0	0
0	0	0	0	0	0
0	0	0	0	0	0
0	0	0	0	0	0
0	0	0	0	0	0
0	0	0	0	0	0
0	0	0	0	0	0
0	0	0	0	0	0
0	0	0	0	0	0
0	0	0	0	0	0
0	0	0	0	0	0
0	1	0	1	0	0
0	0	0	0	0	0
0	0	0	0	0	0
0	0	0	0	0	0
0	0	0	0	0	0
0	0	0	0	0	0
0	0	0	0	0	0
0	0	0	0	0	0
0	0	0	0	0	0
0	0	0	0	0	0
0	0	0	0	0	0
0	0	0	0	0	0
0	0	0	1	0	0
0	0	0	0	0	0
0	0	0	0	0	0
0	0	0	0	0	0
0	0	0	0	0	0
0	0	0	0	0	0
0	0	0	0	0	0
0	0	0	0	0	0
0	0	0	0	0	0
0	0	0	0	0	0
0	0	0	0	0	0
0	0	0	0	0	0
0	0	0	0	0	0
0	0	0	0	0	0
0	0	0	0	0	0
0	0	0	0	0	0
0	0	0	0	0	0
0	0	0	0	0	0
0	0	0	0	0	0
0	0	0	0	0	0
0	0	0	0	0	0
0	0	0	0	0	0
0	0	0	0	0	0
0	0	0	0	0	0
0	0	0	0	0	0
0	0	0	0	0	0
0	0	0	0	0	0
0	0	0	0	0	0
0	0	0	1	0	0
0	0	0	0	0	0
0	0	0	0	0	0
0	0	0	0	0	0
0	0	0	0	0	0

0	0	0	0	0	0
0	0	0	0	0	0
0	0	0	0	0	0
0	0	0	0	0	0
0	0	0	0	0	0
0	0	0	0	0	0
0	0	0	0	0	0
0	0	0	0	0	0
0	0	0	0	0	0
0	0	0	0	0	0
0	0	0	0	0	0
0	0	0	0	0	0
0	0	0	0	0	0
0	0	0	0	0	0
1	0	1	0	0	0
0	0	0	0	0	0
0	0	0	0	0	0
0	0	0	0	0	0
0	0	0	0	0	0
0	0	0	0	0	0
0	0	0	0	0	0
0	0	0	0	0	0
0	0	0	0	0	0
0	1	0	0	0	0
0	0	0	0	0	0
0	0	0	0	0	0
0	0	0	0	0	0
0	0	0	0	0	0
0	0	0	0	0	0
0	0	0	0	0	0
0	0	0	0	0	0
0	0	0	0	0	0
0	0	0	0	0	0
0	0	0	0	0	0

Somatisierungsstörung	Essstörung	Dermatologie	HNO	Augenheilkunde	Kinderheilkunde	Med	CHIR
0	0	0	0	0	0	.00	1.00
0	0	0	0	0	0	.00	1.00
0	0	0	0	0	0	1.00	.00
0	0	0	0	0	0	1.00	.00
0	0	0	0	0	0	1.00	.00
0	0	0	0	0	0	.00	1.00
0	0	0	0	1	0	.00	1.00
0	1	0	0	0	0	.00	.00
0	0	0	0	0	0	.00	1.00
0	0	0	0	0	0	.00	1.00
0	0	0	0	1	0	.00	1.00
0	0	0	0	0	0	.00	1.00
0	0	0	0	0	0	1.00	.00
0	0	0	0	0	0	.00	1.00
0	0	0	0	0	0	.00	.00
0	0	0	0	0	0	1.00	.00
0	0	0	0	0	0	.00	.00
0	0	0	0	0	0	.00	1.00
0	0	0	0	0	0	1.00	.00
0	0	0	0	0	0	1.00	.00
0	0	0	0	0	0	.00	.00
0	0	0	0	0	0	1.00	.00
0	0	0	0	0	0	.00	1.00
0	0	0	0	0	0	.00	.00
0	0	0	0	0	0	1.00	.00
0	0	0	0	0	0	.00	1.00
0	0	0	0	0	0	1.00	.00
0	0	0	0	0	0	1.00	.00
0	0	0	0	0	0	.00	1.00
0	0	0	0	0	0	.00	1.00
0	0	0	0	0	0	1.00	.00
0	0	0	0	0	0	1.00	.00
0	0	0	0	0	0	1.00	.00
0	0	0	0	0	0	.00	1.00
0	0	0	0	0	0	.00	.00
0	0	0	0	0	0	1.00	.00
0	0	0	0	0	0	.00	1.00
0	0	0	0	0	0	1.00	.00
0	0	0	0	0	0	.00	1.00
0	0	0	0	0	0	.00	1.00
0	0	0	0	0	0	1.00	.00
0	0	0	0	0	0	1.00	.00
0	0	0	0	0	0	1.00	.00
0	0	0	0	0	0	.00	1.00
0	0	0	0	0	0	.00	1.00
0	0	0	0	0	0	1.00	.00
0	0	0	0	0	0	1.00	.00
0	0	0	0	0	0	1.00	.00
0	0	0	0	0	0	1.00	.00
0	0	0	0	0	0	.00	.00
0	0	0	0	0	0	.00	1.00
0	0	0	0	0	0	.00	1.00
0	0	0	0	0	0	.00	1.00
0	0	0	0	0	0	.00	1.00
0	0	0	0	0	0	1.00	.00
0	0	0	0	0	0	1.00	.00
0	0	0	0	0	0	1.00	.00
0	0	?	0	0	0	.00	.00
0	0	0	0	0	0	.00	1.00
0	0	0	0	0	0	1.00	.00
0	0	0	0	0	0	1.00	.00
0	0	0	0	0	0	.00	1.00
0	0	0	0	0	0	.00	.00
0	0	0	0	0	0	1.00	.00
0	0	0	0	0	0	1.00	.00
0	0	0	0	0	0	.00	1.00
0	0	0	0	0	0	.00	1.00
0	0	0	0	0	0	.00	1.00
0	0	0	0	0	0	1.00	.00
0	0	0	0	0	0	.00	.00
0	0	0	0	0	0	1.00	.00
0	0	0	0	0	0	.00	1.00
0	0	0	0	0	0	.00	1.00
0	0	0	0	0	0	.00	.00
0	0	0	0	0	0	.00	.00
0	0	0	0	0	0	1.00	.00

0	0	0	0	0	0	.00	1.00
0	0	0	0	0	0	1.00	.00
0	0	0	0	0	0	1.00	.00
0	0	0	0	0	0	1.00	.00
0	0	0	0	0	0	.00	1.00
0	0	0	0	0	0	.00	1.00
0	0	0	0	0	0	1.00	.00
0	0	0	0	0	0	.00	1.00
0	0	0	0	0	0	.00	1.00
0	0	0	0	0	0	1.00	.00
0	0	0	0	0	0	.00	1.00
0	0	0	0	0	0	.00	.00
0	0	0	0	0	0	.00	1.00
0	0	0	0	0	0	1.00	.00
0	0	0	0	0	0	1.00	.00
0	0	0	0	0	0	.00	1.00
0	0	0	0	0	0	1.00	.00
0	0	0	0	0	0	.00	1.00
0	0	0	0	0	0	1.00	.00
0	0	0	0	0	0	1.00	.00
0	0	0	0	0	0	.00	1.00
0	0	0	0	0	0	.00	1.00
0	0	0	0	0	0	.00	.00
0	0	0	0	0	0	1.00	.00
0	0	0	0	0	0	1.00	.00
0	0	0	0	0	0	.00	.00
0	0	0	0	0	0	.00	.00
0	0	0	0	0	0	1.00	.00
0	0	0	0	0	0	1.00	.00
0	0	0	0	0	0	.00	1.00
0	0	0	0	0	0	1.00	.00
0	0	0	0	0	0	1.00	.00
0	0	0	0	0	0	1.00	.00
0	0	0	0	0	0	.00	1.00
0	0	0	0	0	0	1.00	.00
0	0	0	0	0	0	.00	1.00
0	0	0	0	0	0	.00	1.00
0	0	0	0	0	0	.00	1.00
0	0	0	0	0	0	.00	1.00
0	0	0	0	0	0	1.00	.00
0	0	0	0	0	0	.00	1.00
0	0	0	0	0	0	1.00	.00
0	0	0	1	0	0	.00	.00
0	0	0	0	0	0	.00	1.00
0	0	0	0	0	0	.00	1.00
0	0	0	0	0	0	.00	1.00
0	0	0	0	0	0	1.00	.00
0	0	0	?	0	0	1.00	.00
0	0	0	0	0	0	.00	1.00
0	0	0	0	0	0	.00	1.00
0	0	0	0	0	0	.00	1.00
0	0	0	0	0	0	.00	.00
0	0	0	0	0	0	.00	1.00
0	0	0	0	0	0	.00	1.00
0	0	0	0	0	0	1.00	.00
0	0	0	0	0	0	.00	.00
0	0	0	0	0	0	.00	.00
0	0	0	0	0	0	.00	1.00
0	0	0	0	0	0	.00	1.00
0	0	0	0	0	0	.00	.00
0	0	0	0	0	0	.00	1.00
0	0	0	0	0	0	.00	.00
0	0	0	0	0	0	1.00	.00
0	0	0	0	0	0	.00	.00
0	0	0	0	0	0	.00	1.00
0	0	0	0	0	0	1.00	.00
0	0	0	0	0	0	.00	1.00
0	0	0	0	0	0	.00	1.00
0	0	0	0	0	0	.00	1.00
0	0	0	0	0	0	.00	1.00
0	0	0	0	0	0	1.00	.00
0	0	0	0	0	0	.00	.00
0	0	0	0	0	0	.00	1.00
0	0	0	0	0	0	1.00	.00
0	0	0	0	0	0	.00	1.00
0	0	0	0	0	0	.00	1.00
0	0	0	0	0	0	.00	1.00

0	0	0	0	0	0	.00	.00
0	0	0	0	0	0	.00	1.00
0	0	0	0	0	0	.00	1.00
0	0	0	0	0	0	.00	1.00
0	0	0	0	0	0	1.00	.00
0	0	0	0	0	0	.00	.00
0	0	0	0	0	0	.00	1.00
0	0	0	0	0	0	.00	.00
0	0	0	0	0	0	.00	.00
0	0	0	0	0	0	.00	1.00
0	0	0	0	0	0	.00	.00
0	0	0	0	0	0	.00	1.00
0	0	0	0	0	0	.00	1.00
0	0	0	0	0	0	.00	.00
0	0	0	0	0	0	1.00	.00
0	0	0	0	0	0	.00	1.00
0	0	0	0	0	0	.00	.00
0	0	0	0	0	0	.00	.00
0	0	0	0	0	0	.00	1.00
0	0	0	0	0	0	.00	1.00
0	0	0	0	0	0	.00	1.00
0	0	0	0	0	0	.00	1.00
0	0	0	0	0	0	.00	1.00
0	0	0	0	0	0	1.00	.00
0	0	0	0	0	0	.00	.00
0	0	0	1	0	0	.00	.00
0	0	0	0	0	0	.00	.00
0	0	0	0	0	0	1.00	.00
0	0	0	0	0	0	1.00	.00
0	0	0	0	0	0	.00	1.00
0	0	?	0	0	0	.00	1.00
0	0	0	0	0	0	.00	1.00
0	0	0	0	0	0	1.00	.00
0	0	0	0	0	0	1.00	.00
0	0	0	0	0	0	.00	1.00
0	0	0	0	0	0	1.00	.00
0	0	0	0	0	0	.00	.00
0	0	0	0	0	0	.00	1.00
0	0	0	0	0	0	1.00	.00
0	0	0	0	0	0	.00	1.00
0	0	0	0	0	0	.00	1.00
0	0	0	0	0	0	.00	1.00
0	0	0	0	0	0	.00	1.00
0	0	0	0	0	0	.00	1.00
0	0	0	0	0	0	.00	1.00
0	0	0	0	0	0	.00	1.00
0	0	0	1	0	0	.00	.00
0	0	0	0	0	0	.00	.00
0	0	0	0	0	0	.00	.00
0	0	0	0	0	0	1.00	.00
0	0	0	0	0	0	.00	.00
0	0	0	0	0	0	1.00	.00
0	0	0	0	0	0	.00	.00
0	0	0	0	0	0	.00	1.00
0	0	0	0	0	0	.00	1.00
0	0	0	0	0	0	1.00	.00
0	0	0	0	0	0	.00	1.00
0	0	0	0	0	0	.00	1.00
0	0	0	0	0	0	.00	1.00
0	0	0	0	0	0	1.00	.00
0	0	0	0	0	0	.00	1.00
0	0	0	0	0	0	.00	1.00
0	0	0	0	0	0	.00	1.00
0	0	0	0	0	0	.00	1.00
0	0	0	0	0	0	.00	.00
0	0	0	0	0	0	.00	1.00
0	0	0	0	0	0	1.00	.00
0	0	0	0	0	0	1.00	.00
0	0	0	0	0	0	1.00	.00
0	0	0	0	0	0	.00	1.00
0	0	0	0	0	0	.00	.00
0	0	0	0	0	0	.00	1.00
0	0	0	0	0	0	1.00	.00
0	0	0	0	0	0	.00	1.00
0	0	0	0	0	0	.00	1.00
0	0	0	0	0	0	.00	1.00
0	0	0	0	0	0	.00	.00

0	0	?	0	0	0	.00	.00
0	0	0	0	0	0	.00	1.00
0	0	0	0	0	0	.00	1.00
0	0	0	0	0	0	1.00	.00
0	0	0	0	0	0	.00	1.00
0	0	0	0	0	0	.00	1.00
0	0	0	0	0	0	.00	1.00
0	0	0	0	0	0	.00	1.00
0	0	0	0	0	0	.00	1.00
0	0	0	0	0	0	1.00	.00
0	0	0	0	0	0	.00	1.00
0	0	0	0	0	0	.00	1.00
0	0	0	0	0	0	.00	1.00
0	0	0	0	0	0	1.00	.00
0	0	0	0	0	0	.00	1.00
0	0	0	0	0	0	.00	.00
0	0	0	0	0	0	.00	.00
0	0	0	0	0	0	1.00	.00
0	0	0	0	0	0	.00	1.00
0	0	0	0	0	0	.00	.00
0	0	0	0	0	0	.00	1.00
0	0	0	0	0	0	1.00	.00
0	0	0	0	0	0	1.00	.00
0	0	0	0	0	0	1.00	.00
0	0	0	0	0	0	1.00	.00
0	0	0	0	0	0	.00	1.00
0	0	0	1	0	0	.00	.00
0	0	0	0	0	0	1.00	.00
0	0	0	0	0	0	.00	1.00
0	0	0	0	0	0	.00	.00
0	0	0	0	0	0	.00	1.00
0	0	0	0	0	0	1.00	.00
0	0	0	0	0	0	.00	1.00
0	0	0	0	0	0	1.00	.00
0	0	0	0	0	0	1.00	.00
0	0	0	0	0	0	.00	.00
0	0	0	0	0	0	.00	1.00
0	0	0	0	0	0	1.00	.00
0	0	0	0	0	0	.00	1.00
0	0	0	0	0	0	.00	1.00
0	0	0	0	0	0	.00	1.00
0	0	0	0	0	0	.00	1.00
0	0	0	0	0	0	1.00	.00
0	0	0	0	0	0	.00	1.00
0	0	0	0	0	0	.00	1.00
0	0	0	0	0	0	1.00	.00
0	0	0	0	0	0	.00	.00
0	0	0	0	0	0	.00	1.00
0	0	0	0	0	0	1.00	.00
0	0	1	0	0	0	.00	.00
0	0	0	0	0	0	.00	1.00
0	0	0	0	0	0	.00	.00
0	0	0	0	0	0	.00	1.00
0	0	0	0	0	0	1.00	.00
0	0	0	1	0	0	.00	.00
0	0	0	0	0	0	.00	1.00
0	0	0	0	0	0	.00	.00
0	0	0	0	0	0	.00	1.00
0	0	0	1	0	0	.00	.00
0	0	0	0	0	0	.00	1.00
0	0	0	0	0	0	1.00	.00
0	0	0	0	0	0	.00	1.00
0	0	0	0	0	0	.00	1.00
0	0	0	0	0	0	1.00	.00
0	0	0	0	0	0	1.00	.00
0	0	0	0	0	0	1.00	.00
0	0	0	0	0	0	.00	1.00
0	0	0	0	0	0	1.00	.00
0	0	0	0	0	0	1.00	.00
0	0	0	0	0	0	.00	.00
0	0	0	0	0	0	.00	1.00
0	0	0	0	0	0	.00	.00
0	0	0	0	0	0	.00	1.00
0	0	0	0	0	0	1.00	.00
0	0	0	1	0	0	.00	.00
0	0	0	0	0	0	.00	1.00
0	0	0	0	0	0	.00	.00

0	0	0	0	0	0	1.00	.00
0	0	0	0	0	0	.00	1.00
0	0	0	0	0	0	.00	.00
0	0	0	0	0	0	1.00	.00
0	0	0	0	0	0	.00	1.00
0	0	0	0	0	0	1.00	.00
0	0	0	0	0	0	1.00	.00
0	0	0	0	0	0	.00	.00
0	0	?	0	0	0	1.00	.00
0	0	0	0	0	0	1.00	.00
0	0	0	0	0	0	1.00	.00
0	0	0	0	0	0	.00	.00
0	0	0	0	0	0	.00	1.00
0	0	0	0	0	0	.00	1.00
0	0	0	0	0	0	.00	1.00
0	0	0	0	0	0	1.00	.00
0	0	0	0	0	0	.00	1.00
0	0	0	0	0	0	1.00	.00
0	0	0	0	0	0	.00	1.00
0	0	0	1	0	0	.00	.00
0	0	0	0	0	0	1.00	.00
0	0	0	0	0	0	1.00	.00
0	0	0	1	0	0	.00	.00
0	0	0	0	0	0	.00	1.00
0	0	0	0	0	0	.00	.00
0	0	0	0	0	0	1.00	.00
0	0	0	0	0	0	.00	1.00
0	0	0	0	0	0	1.00	.00
0	0	0	1	0	0	.00	.00
0	0	0	0	0	0	1.00	.00
0	0	0	0	0	0	.00	.00
0	0	0	0	0	0	1.00	.00
0	0	0	0	0	0	1.00	.00
0	0	0	0	0	0	.00	.00
0	0	0	0	0	0	1.00	.00
0	0	0	0	0	0	1.00	.00
0	0	0	1	0	0	.00	.00
0	0	0	0	0	0	.00	1.00
0	0	0	0	0	0	1.00	.00
0	0	0	0	0	0	1.00	.00
0	0	0	0	0	0	.00	1.00
0	0	0	0	0	0	.00	1.00
0	0	0	0	0	0	.00	.00
0	0	0	0	0	0	.00	1.00
0	0	0	0	0	0	.00	1.00
0	0	0	0	0	0	.00	1.00
0	0	0	1	0	0	.00	.00
0	0	0	0	0	0	.00	.00
0	0	0	0	0	0	1.00	.00
0	0	0	0	0	0	1.00	.00
0	0	0	0	0	0	.00	1.00
0	0	0	0	0	0	.00	1.00
0	0	0	0	0	0	1.00	.00
0	0	0	0	0	0	1.00	.00
0	0	0	0	0	0	.00	1.00
0	0	0	0	0	0	1.00	.00
0	0	0	0	0	0	.00	1.00
0	0	0	0	0	0	.00	1.00
0	0	0	0	0	0	.00	.00
0	0	0	0	0	0	.00	1.00
0	0	0	0	0	0	.00	1.00
0	0	0	0	0	0	.00	1.00
0	0	0	0	0	0	1.00	.00
0	0	0	0	0	0	1.00	.00
0	0	0	0	0	0	.00	.00
0	0	0	0	0	0	.00	1.00
0	0	0	0	0	0	.00	1.00
0	0	0	0	0	0	.00	1.00
0	0	0	0	0	0	.00	1.00
0	0	0	0	0	0	.00	1.00
0	0	0	0	0	0	.00	.00
0	0	0	0	0	0	1.00	.00
0	0	0	0	0	0	.00	.00
0	0	0	0	0	0	.00	1.00
0	0	0	0	0	0	1.00	.00
0	0	0	0	0	0	.00	1.00
0	0	0	0	0	0	1.00	.00

0	0	0	0	0	0	1.00	.00
0	0	0	0	0	0	.00	.00
0	0	0	0	0	0	1.00	.00
0	0	0	0	0	0	.00	1.00
0	0	0	0	0	0	.00	1.00
0	0	0	0	0	0	.00	1.00
0	0	0	0	0	0	.00	1.00
0	0	0	0	0	0	1.00	.00
0	0	0	0	0	0	.00	1.00
0	0	0	0	0	0	.00	1.00
0	0	0	1	0	0	1.00	.00
0	0	0	0	0	0	1.00	.00
0	0	0	0	0	0	.00	.00
0	0	0	0	0	0	.00	1.00
0	0	0	0	0	0	1.00	.00
0	0	0	0	0	0	1.00	.00
0	0	0	0	0	0	.00	1.00
0	0	0	0	0	0	1.00	.00
0	0	0	1	0	0	.00	.00
0	0	0	0	0	0	.00	.00
0	0	0	0	0	0	1.00	.00
0	0	0	0	0	0	1.00	.00
0	0	0	0	0	0	1.00	.00
0	0	0	0	0	0	.00	.00
0	0	0	0	0	0	.00	1.00
0	0	0	0	0	0	.00	1.00
0	0	0	0	1	0	.00	1.00
0	0	0	0	0	0	.00	1.00
0	0	0	0	0	0	.00	1.00
0	0	0	0	0	0	.00	1.00
0	0	0	0	0	0	1.00	.00
0	0	0	0	0	0	.00	.00
0	0	0	0	0	0	.00	1.00
0	0	0	0	0	0	.00	.00
0	0	0	1	0	0	.00	.00
0	0	0	0	0	0	.00	1.00
0	0	0	0	0	0	1.00	.00
0	0	0	0	0	0	1.00	.00
0	0	0	0	0	0	.00	1.00
0	0	0	0	0	0	1.00	.00
0	0	0	0	0	0	.00	1.00
0	0	0	0	0	0	1.00	.00
0	0	0	0	0	0	.00	1.00
0	0	0	0	0	0	.00	.00
0	0	0	0	0	0	.00	.00
0	0	0	0	0	0	.00	1.00
0	0	0	0	0	0	1.00	.00
0	0	0	0	0	0	.00	1.00
0	0	1	0	0	0	.00	.00
0	0	0	0	0	0	1.00	.00
0	0	0	0	0	0	.00	1.00
0	0	0	1	0	0	.00	.00
0	0	0	0	0	0	.00	1.00
0	0	0	0	0	0	.00	1.00
0	0	0	0	0	0	.00	.00
0	0	0	0	0	0	.00	1.00
0	0	0	0	0	0	1.00	.00
0	0	0	0	0	0	.00	.00
0	0	0	0	0	0	1.00	.00
0	0	0	1	0	0	1.00	.00
0	0	0	0	0	0	.00	1.00
0	0	0	0	0	0	.00	.00
0	0	0	0	0	0	.00	1.00
0	0	0	0	0	0	1.00	.00
0	0	0	0	0	0	1.00	.00
0	0	0	0	0	0	.00	1.00
0	0	0	0	0	0	1.00	.00
0	0	0	0	0	0	1.00	.00
0	0	0	0	0	0	.00	1.00
0	0	0	1	0	0	.00	.00
0	0	0	0	0	0	.00	1.00
0	0	0	0	0	0	.00	1.00
0	0	0	0	0	0	1.00	.00
0	0	0	0	0	0	1.00	.00
0	0	0	0	0	0	.00	1.00
0	0	0	0	0	0	.00	.00
0	0	0	0	0	0	.00	.00

0	0	0	0	0	0	.00	1.00
0	0	0	0	0	0	.00	.00
0	0	0	0	0	0	1.00	.00
0	0	0	0	0	0	1.00	.00
0	0	0	0	0	0	1.00	.00
0	0	0	0	0	0	.00	1.00
0	0	0	0	0	0	1.00	.00
0	0	0	0	0	0	1.00	.00
0	0	0	0	0	0	1.00	.00
0	0	0	0	0	0	1.00	.00
0	0	0	0	0	0	.00	1.00
0	0	0	0	0	0	.00	1.00
0	0	0	0	0	0	1.00	.00
0	0	0	0	0	0	.00	1.00
0	0	0	0	0	0	.00	1.00
0	0	0	0	0	0	.00	1.00
0	0	0	0	0	0	.00	1.00
0	0	0	0	0	0	.00	1.00
0	0	0	0	0	0	1.00	.00
0	0	0	0	0	0	.00	.00
0	0	0	0	0	0	1.00	.00
0	0	0	1	0	0	1.00	.00
0	0	0	0	0	0	.00	1.00
0	0	0	0	0	0	.00	1.00
0	0	0	0	0	0	1.00	.00
0	0	0	0	0	0	.00	.00
0	0	0	0	0	0	.00	1.00
0	0	0	0	0	0	.00	.00
0	0	0	0	0	0	.00	.00
0	0	0	0	0	0	.00	1.00
0	0	0	0	0	0	.00	1.00
0	0	0	0	0	0	.00	1.00
0	0	0	0	0	0	.00	1.00
0	0	0	0	0	0	1.00	.00
0	0	0	0	0	0	1.00	.00
0	0	0	0	0	0	.00	1.00
0	0	0	0	0	0	1.00	.00
0	0	0	0	0	0	.00	1.00
0	0	0	0	0	0	1.00	.00
0	0	0	0	0	0	.00	.00
0	0	0	0	0	0	.00	1.00
0	0	0	0	0	0	.00	.00
0	0	0	0	0	0	1.00	.00
0	0	0	0	0	0	.00	.00
0	0	0	0	0	0	1.00	.00
0	0	0	0	0	0	.00	1.00
0	0	0	0	0	0	1.00	.00
0	0	0	0	0	0	1.00	.00
0	0	0	0	0	0	.00	.00
0	0	0	0	0	0	.00	1.00
0	0	0	0	0	0	.00	1.00
0	0	0	0	0	0	.00	.00
0	0	0	0	0	0	.00	.00
0	0	0	0	0	0	1.00	.00
0	0	0	0	0	0	.00	1.00
0	0	0	0	0	0	1.00	.00
0	0	0	0	0	0	1.00	.00
0	0	0	0	0	0	.00	.00
0	0	0	0	0	0	.00	1.00
0	0	0	0	0	0	.00	1.00
0	0	0	0	0	0	.00	1.00
0	0	0	0	0	0	.00	1.00
0	0	0	0	0	0	.00	1.00
0	0	0	0	0	0	.00	1.00
0	0	0	0	0	0	.00	1.00
0	0	0	0	0	0	.00	.00
0	0	0	0	0	0	1.00	.00
0	0	0	0	0	0	1.00	.00
0	0	0	0	0	0	.00	1.00
0	0	0	0	0	0	.00	1.00
0	0	0	0	0	0	1.00	.00
0	0	0	0	0	0	.00	1.00
0	0	0	0	0	0	.00	1.00
0	0	0	0	0	0	.00	1.00
0	0	0	0	0	0	1.00	.00
0	0	0	0	0	0	.00	1.00
0	0	0	0	0	0	1.00	.00

0	0	0	0	0	0	.00	1.00
0	0	0	0	0	0	.00	.00
0	0	0	0	0	0	1.00	.00
0	0	0	0	0	0	1.00	.00
0	0	0	0	0	0	.00	.00
0	0	0	0	0	0	.00	1.00
0	0	0	0	0	0	.00	1.00
0	0	0	0	0	0	1.00	.00
0	0	0	0	0	0	1.00	.00
0	0	0	0	0	0	1.00	.00
0	0	0	0	0	0	.00	.00
0	0	0	0	0	0	1.00	.00
0	0	0	0	0	0	1.00	.00
0	0	0	0	0	0	1.00	.00
0	0	0	0	0	0	.00	1.00
0	0	0	0	0	0	.00	1.00
0	0	0	0	0	0	1.00	.00
0	0	0	1	0	0	1.00	.00
0	0	0	0	0	0	.00	.00
0	0	0	0	0	0	1.00	.00
0	0	0	0	0	0	1.00	.00
0	0	0	0	0	0	1.00	.00
0	0	0	0	1	0	.00	1.00
0	0	0	0	0	0	1.00	.00
0	0	0	0	0	0	1.00	.00
0	0	0	0	0	0	.00	1.00
0	0	0	0	0	0	.00	1.00
0	0	0	0	0	0	.00	1.00
0	0	0	0	0	0	1.00	.00
0	0	0	0	0	0	1.00	.00
0	0	0	0	0	0	.00	1.00
0	0	0	0	0	0	1.00	.00
0	0	0	0	0	0	1.00	.00
0	0	0	0	0	0	.00	.00
0	0	0	0	0	0	.00	1.00
0	0	0	0	0	0	.00	1.00
0	0	0	0	0	0	1.00	.00
0	0	0	1	0	0	.00	.00
0	0	0	0	0	0	.00	1.00
0	0	0	0	0	0	.00	1.00
0	0	0	0	0	0	.00	1.00
0	0	0	0	0	0	.00	1.00
0	0	0	0	0	0	1.00	.00
0	0	0	0	0	0	.00	1.00
0	0	0	0	0	0	.00	1.00
0	0	0	0	0	0	1.00	.00
0	0	0	0	0	0	.00	1.00
0	0	0	0	0	0	.00	1.00
0	0	0	0	0	0	1.00	.00
0	0	0	0	0	0	1.00	.00
0	0	0	0	0	0	1.00	.00
0	0	0	0	0	0	.00	1.00
0	0	0	0	0	0	.00	1.00
0	0	0	0	0	0	1.00	.00
0	0	0	0	0	0	.00	1.00
0	0	0	0	0	0	.00	.00
0	0	0	0	0	0	1.00	.00
0	0	0	0	0	0	.00	1.00
0	0	0	0	0	0	.00	1.00
0	0	0	0	0	0	.00	1.00
0	0	?	0	0	0	.00	.00
0	0	0	0	0	0	.00	1.00
0	0	0	0	0	0	1.00	.00
0	0	0	0	0	0	1.00	.00
0	0	0	0	0	0	.00	1.00
0	0	0	0	0	0	1.00	.00
0	0	0	0	0	0	.00	1.00
0	0	0	0	0	0	.00	.00
0	0	0	0	0	0	.00	1.00
0	0	0	0	0	0	.00	1.00
0	0	0	0	0	0	.00	.00
0	0	0	0	0	0	1.00	.00
0	0	0	0	0	0	.00	1.00
0	0	0	0	0	0	.00	1.00
0	0	0	0	0	0	1.00	.00
0	0	0	0	0	0	1.00	.00
0	0	0	0	0	0	1.00	.00

0	0	0	0	0	0	.00	1.00
0	0	0	0	1	0	1.00	.00
0	0	0	0	0	0	.00	.00
0	0	0	0	0	0	.00	1.00
0	0	0	0	0	0	1.00	.00
0	0	0	0	0	0	.00	1.00
0	0	0	0	0	0	1.00	.00
0	0	0	0	0	0	.00	1.00
0	0	0	0	0	0	.00	1.00
0	0	0	0	0	0	.00	1.00
0	0	0	0	0	0	1.00	.00
0	0	0	0	1	0	.00	1.00
0	0	0	0	0	0	1.00	.00
0	0	0	0	0	0	.00	1.00
0	0	0	0	0	0	1.00	.00
0	0	0	0	0	0	.00	1.00
0	0	0	0	0	0	1.00	.00
0	0	0	0	0	0	.00	1.00
0	0	0	0	0	0	.00	.00
0	0	0	0	0	0	.00	1.00
0	0	0	0	0	0	.00	1.00
0	0	0	0	0	0	.00	1.00
0	0	0	0	0	0	.00	1.00
0	0	0	0	1	0	.00	1.00
0	0	0	0	0	0	1.00	.00
0	0	0	0	0	0	1.00	.00
0	0	0	1	0	0	.00	1.00
0	0	0	0	0	0	1.00	.00
0	0	0	0	0	0	1.00	.00
0	0	0	0	0	0	1.00	.00
0	0	0	0	0	0	.00	1.00
0	0	0	0	0	0	.00	1.00
0	0	0	0	0	0	.00	.00
0	0	0	0	0	0	1.00	.00
0	0	0	0	0	0	1.00	.00
0	0	0	0	0	0	.00	.00
0	0	0	0	0	0	1.00	.00
0	0	0	0	0	0	1.00	.00
0	0	0	0	0	0	1.00	.00
0	0	0	0	0	0	.00	1.00
0	0	0	0	0	0	1.00	.00
0	0	0	0	0	0	.00	1.00
0	0	0	0	0	0	1.00	.00
0	0	0	0	0	0	.00	1.00
0	0	0	0	0	0	.00	1.00
0	0	0	0	0	0	.00	.00
0	0	0	0	0	0	1.00	.00
0	0	0	0	0	0	.00	1.00
0	0	0	0	0	0	.00	1.00
0	0	0	0	0	0	1.00	.00
0	0	0	0	0	0	.00	1.00
0	1	0	0	0	0	.00	.00
0	0	0	0	0	0	.00	1.00
0	0	0	0	0	0	1.00	.00
0	0	0	0	0	0	.00	1.00
0	0	0	0	0	0	.00	1.00
0	0	0	0	0	0	.00	.00
0	0	0	0	0	0	.00	1.00
0	0	0	0	0	0	1.00	.00
0	0	0	0	0	0	.00	1.00
0	0	0	0	0	0	.00	1.00
0	0	0	0	0	0	.00	.00
0	0	0	0	0	0	.00	1.00
0	0	0	0	0	0	.00	1.00
0	0	0	1	0	0	.00	.00
0	0	0	0	0	0	.00	1.00
0	0	0	0	0	0	.00	1.00
0	0	0	0	0	0	.00	1.00
0	0	0	0	0	0	1.00	.00
0	0	0	0	1	0	1.00	.00
0	0	0	0	0	0	.00	.00
0	0	0	0	0	0	.00	1.00
0	0	0	0	0	0	.00	1.00
0	0	0	0	0	0	.00	1.00
0	0	0	0	0	0	.00	1.00
0	0	0	0	0	0	.00	.00
0	0	0	0	0	0	1.00	.00

0	0	0	0	0	0	.00	.00
0	0	0	0	0	0	.00	1.00
0	0	0	0	0	0	1.00	.00
0	0	0	0	1	0	.00	1.00
0	0	0	1	0	0	.00	.00
0	0	0	0	0	0	1.00	.00
0	0	0	0	0	0	1.00	.00
0	0	0	0	0	0	1.00	.00
0	0	0	0	0	0	1.00	.00
0	0	0	0	0	0	1.00	.00
0	0	0	0	0	0	.00	.00
0	0	0	0	0	0	.00	1.00
0	0	0	0	0	0	.00	1.00
0	0	0	0	0	0	.00	1.00
0	0	0	0	0	0	.00	1.00
0	0	0	0	0	0	.00	.00
0	0	0	0	0	0	.00	.00
0	0	0	0	0	0	.00	1.00
0	0	0	0	0	0	1.00	.00
0	0	1	0	0	0	.00	.00
0	0	0	0	0	0	.00	1.00
0	0	0	0	0	0	1.00	.00
0	0	0	0	0	0	.00	.00
1	0	0	0	0	0	.00	.00
0	0	0	0	0	0	1.00	.00
0	0	0	0	0	0	.00	1.00
0	0	0	0	0	0	1.00	.00
0	0	0	0	0	0	1.00	.00
0	0	0	0	0	0	1.00	.00
0	0	0	0	0	0	1.00	.00
0	0	0	0	0	0	.00	1.00
0	0	0	0	0	0	.00	.00
0	0	0	0	0	0	.00	1.00
0	0	0	0	0	0	1.00	.00
0	0	0	0	0	0	1.00	.00
0	0	0	0	0	0	1.00	.00
0	0	?	0	0	0	1.00	.00
0	0	0	0	0	0	1.00	.00
0	0	0	0	0	0	.00	1.00
0	0	0	?	0	0	1.00	.00
0	0	0	1	0	0	1.00	.00
0	0	0	0	0	0	.00	1.00
0	0	0	0	0	0	.00	1.00
0	0	0	0	0	0	.00	1.00
0	0	0	0	0	0	1.00	.00
0	0	0	0	0	0	.00	1.00
0	0	0	0	0	0	.00	1.00
0	0	0	0	0	0	1.00	.00
0	0	0	0	0	0	1.00	.00
0	0	0	0	0	0	1.00	.00
0	0	0	0	0	0	1.00	.00
0	0	0	0	0	0	1.00	.00
0	0	0	0	0	0	.00	1.00
0	0	0	0	0	0	.00	1.00
0	0	0	0	0	0	1.00	.00
0	0	0	0	0	0	.00	.00
0	0	0	0	0	0	.00	.00
0	0	0	0	0	0	.00	1.00
0	0	0	0	0	0	.00	.00
0	0	0	0	0	0	.00	1.00
0	0	0	0	0	0	.00	.00
0	0	0	0	0	0	.00	1.00
0	0	0	0	0	0	.00	1.00
0	0	0	0	0	0	.00	.00
0	0	0	0	0	0	1.00	.00
0	0	0	0	0	0	1.00	.00
0	0	0	0	0	0	.00	1.00
0	0	0	0	0	0	.00	1.00
0	0	0	0	0	0	.00	.00
0	0	0	0	0	0	1.00	.00
0	0	0	0	0	0	.00	.00
0	0	0	0	0	0	1.00	.00
0	0	0	0	0	0	1.00	.00
0	0	0	0	0	0	1.00	.00
0	0	0	0	0	0	.00	1.00
0	0	0	0	0	0	.00	.00
0	0	0	0	0	0	1.00	.00

0	0	0	0	0	0	.00	1.00
0	0	0	0	0	0	1.00	.00
0	0	0	0	0	0	1.00	.00
0	0	0	0	0	0	1.00	.00
0	0	0	0	0	0	.00	.00
0	0	0	0	0	0	1.00	.00
0	0	0	0	0	0	.00	1.00
0	0	0	0	0	0	.00	.00
0	0	0	0	0	0	.00	1.00
0	0	0	0	0	0	.00	1.00
0	0	0	0	0	0	1.00	.00
0	0	0	0	0	0	1.00	.00
0	0	0	0	0	0	.00	.00
0	0	0	0	0	0	.00	1.00
0	0	0	0	0	0	1.00	.00
0	0	0	0	0	0	.00	1.00
0	0	0	0	0	0	1.00	.00
0	0	0	0	0	0	.00	1.00
0	0	0	0	0	0	1.00	.00
0	0	0	0	0	0	.00	1.00
0	0	0	0	0	0	.00	1.00
0	0	0	0	1	0	.00	1.00
0	0	0	0	0	0	.00	.00
0	0	0	0	0	0	1.00	.00
0	0	0	0	0	0	.00	.00
0	0	0	0	0	0	1.00	.00
0	0	0	0	0	0	.00	.00
0	0	0	0	0	0	.00	1.00
0	0	0	0	0	0	.00	.00
0	0	0	0	0	0	.00	.00
0	0	0	0	0	0	.00	1.00
0	0	0	0	0	0	.00	1.00
0	0	0	0	0	0	.00	.00
0	0	1	0	0	0	.00	.00
0	0	0	0	0	0	.00	1.00
0	0	0	0	0	0	1.00	.00
0	0	0	0	0	0	1.00	.00
0	0	0	0	0	0	1.00	.00
0	0	1	0	0	0	.00	.00
0	0	0	0	0	0	.00	1.00
0	0	0	0	0	0	1.00	.00
0	0	0	1	0	0	.00	.00
0	0	0	0	0	0	.00	1.00
0	0	0	0	0	0	.00	1.00
0	0	0	0	0	0	.00	.00
0	0	0	0	0	0	.00	1.00
0	0	0	0	0	0	1.00	.00
0	0	0	0	0	0	1.00	.00
0	0	0	0	0	0	1.00	.00
0	0	0	0	0	0	.00	1.00
0	0	0	0	1	0	.00	1.00
0	0	0	0	0	0	.00	.00
0	0	0	1	0	0	.00	.00
0	0	0	0	0	0	1.00	.00
0	0	0	0	0	0	.00	1.00
0	0	0	0	0	0	1.00	.00
0	0	0	0	0	0	1.00	.00
0	0	0	0	0	0	1.00	.00
0	0	0	1	0	0	1.00	.00
0	0	0	0	0	0	1.00	.00
0	0	0	0	0	0	1.00	.00
0	0	0	0	0	0	.00	1.00
0	0	0	0	0	0	1.00	.00
0	0	0	0	0	0	1.00	.00
0	0	0	0	0	0	.00	1.00
0	0	0	0	0	0	1.00	.00
0	0	0	0	0	0	.00	1.00
0	0	0	0	0	0	1.00	.00
0	0	0	0	0	0	.00	1.00
0	0	0	0	0	0	1.00	.00
0	0	0	1	0	0	.00	.00
0	0	0	0	0	0	1.00	.00
0	0	0	0	0	0	.00	.00
0	0	1	0	0	0	.00	.00
0	0	0	0	1	0	.00	.00
0	0	0	0	1	0	.00	1.00
0	0	0	0	0	0	.00	.00

0	0	0	0	0	0	.00	1.00
0	0	0	0	0	0	.00	1.00
0	0	0	0	0	0	.00	.00
0	0	0	0	0	0	1.00	.00
0	0	0	0	0	0	1.00	.00
0	0	0	0	0	0	1.00	.00
0	0	0	0	0	0	.00	1.00
0	0	0	0	1	0	.00	1.00
0	0	0	0	0	0	.00	1.00
0	0	0	0	0	0	.00	1.00
0	0	0	0	0	0	1.00	.00
0	0	0	0	0	0	1.00	.00
0	0	0	0	0	0	.00	.00
0	0	0	1	0	0	.00	1.00
0	0	0	0	0	0	.00	.00
0	0	0	0	0	0	.00	1.00
0	0	0	0	0	0	1.00	.00
0	0	0	0	0	0	.00	1.00
0	0	0	0	0	0	.00	1.00
0	0	0	0	0	0	.00	.00
0	0	0	0	0	0	1.00	.00
0	0	0	0	0	0	1.00	.00
0	0	0	0	0	0	1.00	.00
0	0	0	0	0	0	.00	.00
0	0	0	0	0	0	.00	.00
0	0	0	0	0	0	.00	.00
0	0	0	0	0	0	.00	1.00
0	0	0	0	0	0	.00	.00
0	0	0	0	0	0	1.00	.00
0	0	0	0	0	0	.00	1.00
0	0	0	0	0	0	.00	1.00
0	0	0	0	0	0	.00	.00
0	0	0	0	0	0	.00	.00
0	0	0	0	0	0	.00	.00

PSYCH	OTHER	Chronic_CoMorb
.00	.00	.00
.00	.00	.00
.00	.00	.00
.00	.00	.00
.00	.00	.00
.00	.00	.00
.00	.00	.00
1.00	.00	.00
.00	.00	.00
.00	.00	.00
.00	.00	.00
.00	.00	.00
.00	.00	.00
.00	.00	.00
1.00	.00	.00
.00	.00	.00
1.00	.00	.00
.00	.00	.00
.00	.00	1.00
.00	.00	.00
1.00	.00	.00
.00	.00	.00
.00	.00	.00
1.00	.00	.00
.00	.00	.00
.00	.00	.00
.00	.00	1.00
.00	.00	.00
.00	.00	.00
.00	.00	.00
.00	.00	1.00
.00	.00	1.00
.00	.00	1.00
.00	.00	.00
1.00	.00	.00
.00	.00	.00
.00	.00	.00
.00	.00	1.00
.00	.00	.00
.00	.00	.00
.00	.00	.00
.00	.00	.00
.00	.00	.00
.00	.00	1.00
.00	.00	.00
.00	.00	.00
.00	.00	.00
.00	.00	.00
.00	.00	.00
1.00	.00	1.00
.00	.00	.00
.00	.00	1.00
.00	.00	.00
.00	.00	.00
.00	.00	.00
.00	.00	.00
.00	.00	.00
.00	1.00	.00
.00	.00	.00
.00	.00	.00
.00	.00	.00
.00	.00	.00
1.00	.00	.00
.00	.00	.00
.00	.00	.00
.00	.00	.00
.00	.00	.00
.00	.00	.00
.00	.00	.00
1.00	.00	.00
.00	.00	.00
.00	.00	.00
.00	.00	.00
1.00	.00	.00
1.00	.00	1.00
.00	.00	.00

.00	.00	.00
.00	.00	.00
.00	.00	.00
.00	.00	.00
.00	.00	.00
.00	.00	.00
.00	.00	.00
.00	.00	.00
.00	.00	.00
.00	.00	.00
.00	.00	.00
1.00	.00	.00
.00	.00	.00
.00	.00	.00
.00	.00	.00
.00	.00	1.00
.00	.00	.00
.00	.00	.00
.00	.00	.00
.00	.00	.00
.00	.00	.00
.00	.00	.00
1.00	.00	.00
.00	.00	.00
.00	.00	1.00
1.00	.00	.00
.00	1.00	.00
.00	.00	.00
.00	.00	.00
.00	.00	.00
.00	.00	1.00
.00	.00	.00
.00	.00	1.00
.00	.00	.00
.00	.00	.00
.00	.00	.00
.00	.00	.00
.00	.00	.00
.00	.00	.00
.00	.00	.00
.00	.00	.00
.00	.00	.00
.00	1.00	.00
.00	.00	.00
.00	.00	.00
.00	.00	.00
.00	.00	.00
.00	.00	.00
.00	.00	.00
.00	.00	.00
.00	.00	
1.00	.00	.00
.00	.00	.00
.00	.00	.00
.00	.00	.00
1.00	.00	.00
.00	1.00	1.00
.00	.00	.00
.00	.00	.00
1.00	.00	.00
.00	.00	.00
1.00	.00	.00
.00	.00	.00
1.00	.00	.00
.00	.00	.00
.00	.00	1.00
.00	.00	.00
.00	.00	.00
.00	.00	.00
.00	.00	.00
.00	.00	.00
1.00	.00	.00
.00	.00	.00
.00	.00	.00
.00	.00	.00
.00	.00	.00
.00	.00	.00

1.00	.00	.00
.00	.00	1.00
.00	.00	.00
.00	.00	.00
.00	.00	1.00
1.00	.00	.00
.00	.00	.00
1.00	.00	.00
1.00	.00	.00
.00	.00	.00
1.00	.00	.00
.00	.00	.00
.00	.00	.00
1.00	.00	.00
.00	.00	.00
.00	.00	.00
1.00	.00	1.00
1.00	.00	.00
.00	.00	.00
.00	.00	.00
.00	.00	.00
.00	.00	.00
.00	.00	.00
.00	.00	.00
.00	1.00	.00
.00	1.00	.00
1.00	.00	.00
.00	.00	.00
.00	.00	
.00	.00	.00
.00	.00	.00
.00	.00	.00
.00	.00	.00
.00	.00	.00
.00	.00	.00
.00	.00	.00
1.00	.00	.00
.00	.00	.00
.00	.00	.00
.00	.00	.00
.00	.00	.00
.00	.00	.00
.00	.00	.00
.00	.00	.00
.00	.00	.00
.00	.00	1.00
.00	1.00	.00
.00	1.00	.00
1.00	.00	1.00
.00	.00	.00
1.00	.00	.00
.00	.00	1.00
1.00	.00	.00
.00	.00	.00
.00	.00	.00
.00	.00	.00
.00	.00	.00
.00	.00	.00
.00	.00	.00
.00	.00	.00
.00	.00	.00
.00	.00	.00
.00	.00	.00
.00	.00	.00
1.00	.00	.00
.00	.00	.00
.00	.00	.00
.00	.00	.00
.00	.00	.00
.00	.00	.00
1.00	.00	
.00	.00	.00
.00	.00	.00
.00	.00	.00
.00	.00	.00
.00	.00	.00
1.00	.00	.00

.00	1.00	.00
.00	.00	.00
.00	.00	.00
.00	.00	.00
.00	.00	.00
.00	.00	.00
.00	.00	.00
.00	.00	.00
.00	.00	.00
.00	.00	.00
.00	.00	.00
.00	.00	.00
.00	.00	.00
.00	.00	.00
.00	.00	.00
1.00	.00	.00
1.00	.00	.00
.00	.00	.00
.00	.00	.00
1.00	.00	.00
.00	.00	.00
.00	.00	1.00
.00	.00	1.00
.00	.00	.00
.00	.00	1.00
.00	.00	.00
.00	1.00	.00
.00	.00	.00
.00	.00	.00
.00	1.00	.00
.00	.00	.00
.00	.00	.00
.00	.00	.00
.00	.00	.00
.00	.00	1.00
1.00	.00	.00
.00	.00	.00
.00	.00	.00
.00	.00	.00
.00	.00	.00
.00	.00	.00
.00	.00	.00
.00	.00	.00
.00	.00	.00
.00	.00	.00
.00	.00	.00
1.00	.00	.00
.00	.00	.00
.00	.00	1.00
.00	1.00	.00
.00	.00	.00
1.00	.00	.00
.00	.00	.00
.00	.00	.00
.00	1.00	.00
.00	.00	.00
1.00	.00	.00
.00	.00	.00
.00	1.00	.00
.00	.00	.00
.00	.00	1.00
.00	.00	1.00
.00	.00	.00
.00	.00	.00
.00	.00	.00
.00	.00	.00
.00	.00	.00
.00	.00	.00
.00	.00	.00
1.00	.00	.00
.00	.00	.00
1.00	.00	.00
.00	.00	.00
.00	.00	.00
.00	1.00	.00
.00	.00	1.00
1.00	.00	.00

.00	.00	.00
.00	.00	.00
1.00	.00	.00
.00	.00	.00
.00	.00	.00
.00	.00	.00
.00	.00	.00
1.00	.00	.00
.00	.00	.00
.00	.00	.00
.00	.00	.00
1.00	.00	.00
.00	.00	.00
.00	.00	.00
.00	.00	.00
.00	.00	.00
.00	.00	.00
.00	.00	.00
.00	.00	.00
.00	1.00	.00
.00	.00	.00
.00	.00	.00
.00	1.00	.00
.00	.00	.00
1.00	.00	.00
.00	.00	.00
.00	.00	.00
.00	.00	.00
.00	1.00	.00
.00	.00	.00
1.00	.00	
.00	.00	1.00
.00	.00	.00
1.00	.00	.00
.00	.00	.00
.00	.00	.00
.00	1.00	.00
.00	.00	.00
.00	.00	.00
.00	.00	.00
.00	.00	.00
.00	.00	.00
1.00	.00	.00
.00	.00	.00
.00	.00	.00
.00	.00	.00
.00	1.00	.00
1.00	.00	.00
.00	.00	.00
.00	.00	.00
.00	.00	.00
.00	.00	.00
.00	.00	.00
.00	.00	.00
.00	.00	.00
.00	.00	.00
.00	.00	.00
.00	.00	.00
1.00	.00	.00
.00	.00	.00
.00	.00	.00
.00	.00	.00
.00	.00	.00
.00	.00	.00
1.00	.00	1.00
.00	.00	.00
.00	.00	.00
.00	.00	.00
.00	.00	.00
.00	.00	.00
1.00	.00	.00
.00	.00	.00
1.00	.00	.00
.00	.00	.00
.00	.00	1.00
.00	.00	.00
.00	.00	.00

.00	.00	.00
1.00	.00	.00
.00	.00	.00
.00	.00	.00
.00	.00	.00
.00	.00	.00
.00	.00	.00
.00	.00	1.00
.00	.00	.00
.00	.00	.00
.00	.00	.00
.00	.00	.00
1.00	.00	.00
.00	.00	.00
.00	.00	.00
.00	.00	.00
.00	.00	.00
.00	.00	.00
.00	1.00	.00
1.00	.00	.00
.00	.00	.00
.00	.00	.00
.00	.00	.00
1.00	.00	.00
.00	.00	.00
.00	.00	1.00
.00	.00	.00
.00	.00	.00
.00	.00	.00
.00	.00	.00
.00	.00	.00
1.00	.00	1.00
.00	.00	.00
1.00	.00	.00
.00	1.00	.00
.00	.00	.00
.00	.00	1.00
.00	.00	.00
.00	.00	.00
.00	.00	.00
.00	.00	.00
.00	.00	1.00
.00	.00	.00
1.00	.00	.00
1.00	.00	.00
.00	.00	.00
.00	.00	.00
.00	.00	.00
.00	1.00	.00
.00	.00	.00
.00	.00	.00
.00	1.00	.00
.00	.00	.00
.00	.00	.00
1.00	.00	.00
.00	.00	.00
.00	.00	.00
1.00	.00	.00
.00	.00	.00
.00	.00	.00
.00	.00	.00
.00	1.00	.00
.00	.00	1.00
.00	.00	.00
.00	.00	1.00
.00	.00	.00
.00	.00	.00
.00	.00	.00
.00	.00	.00
.00	1.00	.00
.00	.00	.00
.00	.00	.00
.00	.00	1.00
.00	.00	.00
.00	.00	.00
1.00	.00	1.00
1.00	.00	.00

.00	.00	.00
1.00	.00	1.00
.00	.00	.00
.00	.00	.00
.00	.00	.00
.00	.00	.00
.00	.00	.00
.00	.00	1.00
.00	.00	.00
.00	.00	.00
.00	.00	.00
.00	.00	.00
.00	.00	.00
.00	.00	.00
.00	.00	.00
.00	.00	.00
.00	.00	.00
.00	.00	.00
.00	.00	.00
1.00	.00	1.00
.00	.00	1.00
.00	.00	.00
.00	.00	.00
.00	.00	.00
.00	.00	.00
1.00	.00	.00
.00	.00	1.00
.00	1.00	.00
1.00	.00	.00
.00	.00	.00
.00	.00	.00
.00	.00	.00
.00	.00	.00
.00	.00	.00
.00	.00	.00
.00	.00	.00
.00	.00	1.00
.00	.00	.00
.00	.00	.00
1.00	.00	.00
.00	.00	.00
1.00	.00	.00
.00	.00	.00
1.00	.00	.00
.00	.00	.00
.00	.00	.00
.00	.00	1.00
.00	.00	.00
1.00	.00	.00
.00	.00	.00
.00	.00	.00
1.00	.00	.00
1.00	.00	.00
.00	.00	.00
.00	.00	.00
.00	.00	.00
.00	.00	.00
1.00	.00	.00
.00	.00	.00
.00	.00	.00
.00	.00	.00
.00	.00	1.00
.00	.00	.00
.00	.00	.00
.00	.00	.00
.00	1.00	.00
.00	.00	.00
.00	.00	.00
.00	.00	.00
.00	.00	.00
.00	.00	.00
.00	.00	.00
.00	.00	.00
.00	.00	1.00
.00	.00	.00
.00	.00	.00
.00	.00	.00

.00	.00	.00
.00	1.00	.00
.00	.00	.00
.00	.00	.00
1.00	.00	1.00
.00	.00	.00
.00	.00	.00
.00	.00	1.00
.00	.00	.00
.00	.00	.00
1.00	.00	.00
.00	.00	.00
.00	.00	.00
.00	.00	.00
.00	.00	.00
.00	.00	.00
.00	.00	.00
.00	.00	.00
1.00	.00	.00
.00	.00	.00
.00	.00	.00
.00	.00	.00
.00	.00	.00
.00	.00	.00
.00	.00	1.00
.00	.00	.00
.00	.00	.00
.00	.00	.00
.00	.00	.00
.00	.00	.00
.00	.00	.00
.00	.00	.00
.00	.00	.00
1.00	.00	.00
.00	.00	1.00
.00	.00	.00
.00	.00	.00
.00	1.00	.00
.00	.00	.00
.00	.00	.00
.00	.00	.00
.00	.00	.00
.00	.00	1.00
.00	.00	.00
.00	.00	1.00
.00	.00	.00
.00	.00	.00
.00	.00	.00
.00	.00	.00
.00	.00	.00
.00	.00	.00
.00	.00	.00
.00	.00	.00
.00	.00	.00
.00	.00	.00
1.00	.00	1.00
.00	.00	.00
.00	.00	.00
.00	.00	.00
.00	.00	.00
.00	1.00	.00
.00	.00	.00
.00	.00	.00
.00	.00	1.00
.00	.00	.00
.00	.00	1.00
.00	.00	.00
1.00	.00	.00
.00	.00	.00
.00	.00	.00
1.00	.00	.00
.00	.00	1.00
.00	.00	1.00
.00	.00	.00
.00	.00	.00
.00	.00	.00
.00	.00	1.00

.00	.00	1.00
.00	.00	.00
1.00	.00	.00
.00	.00	.00
.00	.00	.00
.00	.00	.00
.00	.00	.00
.00	.00	.00
.00	.00	.00
.00	.00	.00
.00	.00	.00
.00	.00	.00
.00	.00	.00
.00	.00	.00
.00	.00	.00
.00	.00	.00
.00	.00	.00
.00	.00	.00
1.00	.00	.00
.00	.00	.00
.00	.00	.00
.00	.00	.00
.00	.00	1.00
.00	.00	.00
.00	.00	.00
.00	.00	.00
.00	.00	.00
.00	.00	.00
.00	.00	.00
.00	.00	.00
.00	.00	.00
.00	.00	.00
1.00	.00	.00
.00	.00	.00
.00	.00	1.00
1.00	.00	.00
.00	.00	.00
.00	.00	.00
.00	.00	.00
.00	.00	.00
.00	.00	1.00
.00	.00	.00
.00	.00	.00
.00	.00	.00
.00	.00	.00
1.00	.00	.00
.00	.00	1.00
.00	.00	.00
.00	.00	1.00
.00	.00	
.00	.00	.00
1.00	.00	1.00
.00	.00	.00
.00	.00	.00
.00	.00	.00
.00	.00	.00
1.00	.00	.00
.00	.00	.00
.00	.00	.00
.00	.00	.00
.00	.00	.00
.00	1.00	.00
.00	.00	.00
.00	.00	.00
.00	1.00	.00
.00	.00	.00
.00	.00	.00
.00	.00	.00
.00	.00	.00
.00	.00	.00
1.00	.00	.00
.00	.00	.00
.00	.00	.00
.00	.00	.00
.00	.00	.00
1.00	.00	.00
.00	.00	.00

1.00	.00	.00
.00	.00	.00
.00	.00	.00
.00	.00	.00
.00	1.00	.00
.00	.00	1.00
.00	.00	.00
.00	.00	.00
.00	.00	.00
.00	.00	.00
1.00	.00	.00
.00	.00	.00
.00	.00	.00
.00	.00	1.00
.00	.00	.00
1.00	.00	.00
1.00	.00	.00
.00	.00	.00
.00	.00	.00
.00	1.00	.00
.00	.00	.00
.00	.00	.00
1.00	.00	.00
1.00	.00	.00
.00	.00	.00
.00	.00	.00
.00	.00	.00
.00	.00	.00
.00	.00	.00
.00	.00	.00
.00	.00	.00
1.00	.00	.00
.00	.00	.00
.00	.00	.00
.00	.00	.00
.00	.00	.00
.00	.00	.00
.00	.00	1.00
.00	.00	.00
.00	.00	.00
.00	.00	.00
.00	.00	.00
.00	.00	.00
.00	.00	.00
.00	.00	.00
.00	.00	.00
.00	.00	.00
.00	.00	.00
.00	.00	.00
.00	.00	.00
.00	.00	.00
.00	.00	.00
.00	.00	.00
.00	.00	.00
.00	.00	.00
1.00	.00	.00
1.00	.00	.00
.00	.00	.00
1.00	.00	.00
.00	.00	.00
1.00	.00	.00
.00	.00	1.00
.00	.00	.00
1.00	.00	.00
.00	.00	.00
.00	.00	.00
.00	.00	.00
.00	.00	.00
1.00	.00	.00
.00	.00	1.00
1.00	.00	.00
.00	.00	.00
.00	.00	.00
.00	.00	.00
.00	.00	.00
1.00	.00	.00
.00	.00	1.00

.00	.00	.00
.00	.00	1.00
.00	.00	.00
.00	.00	.00
.00	1.00	.00
.00	.00	1.00
.00	.00	.00
1.00	.00	.00
.00	.00	.00
.00	.00	.00
.00	.00	.00
.00	.00	.00
1.00	.00	.00
.00	.00	.00
.00	.00	.00
.00	.00	.00
.00	.00	.00
.00	.00	.00
.00	.00	.00
.00	.00	.00
.00	.00	.00
.00	.00	.00
1.00	.00	.00
.00	.00	.00
1.00	.00	.00
.00	.00	.00
1.00	.00	1.00
.00	.00	.00
1.00	.00	.00
1.00	.00	.00
.00	.00	.00
.00	.00	.00
1.00	.00	.00
.00	1.00	.00
.00	.00	.00
.00	.00	.00
.00	.00	.00
.00	.00	1.00
.00	1.00	.00
.00	.00	.00
.00	.00	.00
.00	1.00	.00
.00	.00	1.00
.00	.00	.00
1.00	.00	.00
.00	.00	.00
.00	.00	.00
.00	.00	1.00
.00	.00	.00
.00	.00	.00
.00	.00	.00
1.00	.00	.00
.00	1.00	.00
.00	.00	.00
.00	.00	.00
.00	.00	.00
.00	.00	1.00
.00	.00	.00
.00	.00	.00
.00	.00	.00
.00	.00	.00
.00	.00	.00
.00	.00	.00
.00	.00	.00
.00	.00	.00
.00	.00	.00
.00	.00	.00
.00	.00	.00
.00	.00	.00
.00	.00	.00
.00	1.00	.00
.00	.00	.00
1.00	.00	.00
1.00	.00	.00
1.00	.00	.00
.00	.00	.00
1.00	.00	.00

.00	.00	1.00
.00	.00	.00
1.00	.00	.00
.00	.00	1.00
.00	.00	.00
.00	.00	.00
.00	.00	1.00
.00	.00	.00
.00	.00	.00
.00	.00	.00
.00	.00	1.00
.00	.00	.00
1.00	.00	.00
.00	.00	.00
1.00	.00	.00
.00	.00	.00
.00	.00	.00
.00	.00	.00
.00	.00	.00
1.00	.00	.00
.00	.00	.00
.00	.00	.00
.00	.00	.00
1.00	.00	.00
1.00	.00	.00
1.00	.00	1.00
.00	.00	.00
1.00	.00	1.00
.00	.00	.00
.00	.00	.00
.00	.00	.00
1.00	.00	.00
1.00	.00	.00
1.00	.00	.00
